# Risk variants and polygenic architecture of disruptive behavior disorders in the context of attention-deficit/hyperactivity disorder

**DOI:** 10.1038/s41467-020-20443-2

**Published:** 2021-01-25

**Authors:** Ditte Demontis, Raymond K. Walters, Veera M. Rajagopal, Irwin D. Waldman, Jakob Grove, Thomas D. Als, Søren Dalsgaard, Marta Ribasas, Jonas Bybjerg-Grauholm, Maria Bækvad-Hansen, Thomas Werge, Merete Nordentoft, Ole Mors, Preben Bo Mortensen, ADHD Working Group of the Psychiatric Genomics Consortium (PGC), Ole A. Andreassen, Maria Jesús Arranz, Tobias Banaschewski, Claiton Bau, Mark Bellgrove, Joseph Biederman, Isabell Brikell, Jan K. Buitelaar, Christie L. Burton, Miguel Casas, Jennifer Crosbie, Alysa E. Doyle, Richard P. Ebstein, Josephine Elia, Corfield C. Elizabeth, Eugenio Grevet, Natalie Grizenko, Alexandra Havdahl, Ziarih Hawi, Johannes Hebebrand, Amaia Hervas, Sarah Hohmann, Jan Haavik, Ridha Joober, Lindsey Kent, Jonna Kuntsi, Kate Langley, Henrik Larsson, Klaus-Peter Lesch, Patrick W. L. Leung, Calwing Liao, Sandra K. Loo, Joanna Martin, Nicholas G. Martin, Sarah E. Medland, Ana Miranda, Nina Roth Mota, Robert D. Oades, Josep Antoni Ramos-Quiroga, Andreas Reif, Marcella Rietschel, Herbert Roeyers, Luis Augusto Rohde, Aribert Rothenberger, Paula Rovira, Cristina Sánchez-Mora, Russell James Schachar, Sarojini Sengupta, Maria Soler Artigas, Hans-Christoph Steinhausen, Anita Thapar, Stephanie H. Witt, Li Yang, Tetyana Zayats, Yanli Zhang-James, Bru Cormand, David M. Hougaard, Benjamin M. Neale, Barbara Franke, Stephen V. Faraone, Anders D. Børglum

**Affiliations:** 1 The Lundbeck Foundation Initiative for Integrative Psychiatric Research, iPSYCH, Aarhus, Denmark; 2 Center for Genomics and Personalized Medicine, Aarhus, Denmark; 3 Department of Biomedicine - Human Genetics, Aarhus University, Aarhus, Denmark; 4 Analytic and Translational Genetics Unit, Department of Medicine, Massachusetts General Hospital and Harvard Medical School, Boston, MA USA; 5 Stanley Center for Psychiatric Research, Broad Institute of MIT and Harvard, Cambridge, MA USA; 6 Department of Psychology, Emory University, Atlanta, GA USA; 7 Bioinformatics Research Centre, Aarhus University, Aarhus, Denmark; 8 National Centre for Register-Based Research, Aarhus University, Aarhus, Denmark; 9 Psychiatric Genetics Unit, Group of Psychiatry, Mental Health and Addiction, Vall d’Hebron Research Institute (VHIR), Universitat Autònoma de Barcelona, Barcelona, Catalonia Spain; 10 Department of Psychiatry, Hospital Universitari Vall d’Hebron, Barcelona, Catalonia Spain; 11 Biomedical Network Research Center on Mental Health (CIBERSAM), Instituto de Salud Carlos III, Madrid, Spain; 12 Departament de Genètica, Microbiologia i Estadística, Facultat de Biologia, Universitat de Barcelona, Barcelona, Catalonia Spain; 13 Center for Neonatal Screening, Department for Congenital Disorders, Statens Serum Institut, Copenhagen, Denmark; 14 GLOBE Institute, Center for GeoGenetics, University of Copenhagen, Copenhagen, Denmark; 15 Institute of Biological Psychiatry, MHC Sct. Hans, Mental Health Services Copenhagen, Roskilde, Denmark; 16 Department of Clinical Medicine, University of Copenhagen, Copenhagen, Denmark; 17 Copenhagen University Hospital, Mental Health Centre Copenhagen Mental Health Services in the Capital Region of Denmark, Hellerup, Denmark; 18 Department of Clinical Medicine, Faculty of Health and Medical Sciences, University of Copenhagen, Copenhagen, Denmark; 19 Psychosis Research Unit, Aarhus University Hospital, Aarhus, Denmark; 20 Centre for Integrated Register-Based Research, Aarhus University, Aarhus, Denmark; 21 Centro de Investigación Biomédica en Red de Enfermedades Raras (CIBERER), Instituto de Salud Carlos III, Madrid, Spain; 22 Institut de Biomedicina de la Universitat de Barcelona (IBUB), Barcelona, Catalonia Spain; 23 Institut de Recerca Sant Joan de Déu (IRSJD), Esplugues de Llobregat, Barcelona, Catalonia Spain; 24 Program in Medical and Population Genetics, Broad Institute of MIT and Harvard, Cambridge, MA USA; 25 Department of Human Genetics, Donders Institute for Brain, Cognition and Behaviour, Radboud University Medical Center, Nijmegen, The Netherlands; 26 Department of Psychiatry, Donders Institute for Brain, Cognition and Behaviour, Radboud University Medical Center, Nijmegen, The Netherlands; 27 Departments of Psychiatry and of Neuroscience and Physiology, SUNY Upstate Medical University, Syracuse, NY USA; 28 NORMENT Centre, Division of Mental Health and Addiction, Oslo University Hospital and University of Oslo, Oslo, Norway; 29 KG Jebsen Centre for Neurodevelopment, Division of Mental Health and Addiction, Oslo University Hospital and University of Oslo, Oslo, Norway; 30 Fundacio Docencia i Recerca Mutua Terrassa, University Hospital Mutua Terrassa, Barcelona, Spain; 31 Department of Child and Adolescent Psychiatry, Central Institute of Mental Health, Medical Faculty Mannheim and University of Heidelberg, Mannheim, Germany; 32 Department of Genetics, Instituto de Biociências, Universidade Federal do Rio Grande do Sul, Porto Alegre, Brazil; 33 Adulthood ADHD Outpatient Program (ProDAH), Clinical Research Center, Hospital de Clínicas de Porto Alegre, Porto Alegre, Brazil; 34 Developmental Psychiatry Program, Experimental Research Center, Hospital de Clínicas de Porto Alegre, Porto Alegre, Brazil; 35 School of Psychological Sciences and Turner Institute for Brain and Mental Health, Monash University, Melbourne, Australia; 36 Department of Psychiatry, Massachusetts General Hospital, Boston, MA USA; 37 Department of Psychiatry, Harvard Medical School, Boston, MA USA; 38 Department of Medical Epidemiology and Biostatistics, Karolinska Instituttet, Stockholm, Sweden; 39 Department of Cognitive Neuroscience, Donders Institute for Brain, Cognition and Behavior, Radboud University Medical Center, Nijmegen, The Netherlands; 40 Karakter Child and Adolescent Psychiatry University Center, Nijmegen, The Netherlands; 41 Psychiatry, Neurosciences and Mental Health, The Hospital for Sick Children, University of Toronto, Toronto, ON Canada; 42 Department of Psychiatry and Forensic Medicine, Universitat Autònoma de Barcelona, Barcelona, Catalonia Spain; 43 Department of Psychiatry, University Hospital Vall d’Hebron, Barcelona, Catalonia Spain; 44 Center for Genomic Medicine and Department of Psychiatry, Massachusetts General Hospital and Harvard Medical School, Boston, MA USA; 45 China Center for Behavior Economics and Finance, Southwestern University of Finance and Economics, (SWUFE), Chengdu, Sichuan China; 46 Department of Pediatrics, Nemours A.I. duPont Hospital for Children, Wilmington, DE USA; 47 Department of Psychiatry, Sidney Kimmel Medical College, Thomas Jefferson University, Philadelphia, PA USA; 48 Department of Mental Disorders, Norwegian Institute of Public Health, Oslo, Norway; 49 Department of Psychiatry, Faculty of Medicine, Universidade Federal do Rio Grande do Sul, Porto Alegre, Brazil; 50 Department of Psychiatry and Douglas Hospital Research Centre, Douglas Mental Health Univerity Institute, McGill University, Montreal, QC Canada; 51 Department of Child and Adolescent Psychiatry, Psychosomatics and Psychotherapy, University Hospital Essen, University of Duisburg-Essen, Essen, Germany; 52 Child and Adolescent Service, University Hospital Mutua Terrassa &Institute of global comprehensive attention to neurodevelopment (IGAIN), Barcelona, Spain; 53 Centre for Neuropsychiatric Disorders, Department of Biomedicine, University of Bergen, Bergen, Norway; 54 Bergen Centre of Brain Plasticity, Division of Psychiatry, Haukeland University Hospital, Bergen, Norway; 55 University of St Andrews, St. Andrews, UK; 56 Social, Genetic and Developmental Psychiatry Centre, Institute of Psychiatry, Psychology and Neuroscience, King’s College London, London, UK; 57 MRC Centre for Neuropsychiatric Genetics & Genomics, School of Medicine, Cardiff University, Cardiff, UK; 58 School of Psychology, Cardiff University, Cardiff, UK; 59 School of Medical Sciences, Örebro University, Örebro, Sweden; 60 Division of Molecular Psychiatry, Center of Mental Health, University of Wuerzburg, Wuerzburg, Germany; 61 Department of Neuroscience, School for Mental Health and Neuroscience (MHENS), Maastricht University, Maastricht, The Netherlands; 62 Laboratory of Psychiatric Neurobiology, Institute of Molecular Medicine, I.M. Sechenov First Moscow State Medical University, Moscow, Russia; 63 Department of Psychology, The Chinese University of Hong Kong, Hong Kong, China; 64 Department of Human Genetics, McGill University, Montreal, QC Canada; 65 Semel Institute for Neuroscience and Human Behavior, UCLA David Geffen School of Medicine, University of California, Los Angeles, CA USA; 66 QIMR Berghofer Medical Research Institute, Brisbane, QLD Australia; 67 Department of Developmental and Educational Psychology, University of Valencia, Valencia, Spain; 68 Department of Human Genetics, Radboud University Medical Center, Nijmegen, The Netherlands; 69 ADHD Outpatient Clinic, Hospital de Clínicas de Porto Alegre, Porto Alegre, Brazil; 70 Clinic for Child and Adolescent Psychiatry and Psychotherapy, University of Duisburg-Essen, Essen, Germany; 71 Department of Psychiatry, Psychosomatic Medicine and Psychotherapy, University Hospital Frankfurt, Frankfurt am Main, Germany; 72 Department of Genetic Epidemiology in Psychiatry, Central Institute of Mental Health, Medical Faculty Mannheim and University of Heidelberg, Mannheim, Germany; 73 Department of Experimental Clinical and Health Psychology, Ghent University, Ghent, Belgium; 74 Department of Psychiatry, Universidade Federal do Rio Grande do Sul, Porto Alegre, Brazil; 75 ADHD Outpatient & Development Psychiatry Program, Hospital de Clínicas de Porto Alegre, Porto Alegre, Brazil; 76 Child and Adolescent Psychiatry, University Medical Center Göttingen, Göttingen, Germany; 77 Department of Child and Adolescent Psychiatry, University of Southern Denmark, Odense, Denmark; 78 Clinical Psychology and Epidemiology, Institute of Psychology, University of Basel, Basel, Switzerland; 79 Department of Child and Adolescent Psychiatry, University of Zurich, Zurich, Switzerland; 80 Child and Adolescent Mental Health Centre, Peking University Sixth Hospital/Institute of Mental Health, Beijing, China; 81 PROMENTA, Oslo University, Oslo, Norway; 82 Department of Psychiatry, SUNY Upstate Medical University, Syracuse, NY USA

**Keywords:** Genome-wide association studies, ADHD

## Abstract

Attention-Deficit/Hyperactivity Disorder (ADHD) is a childhood psychiatric disorder often comorbid with disruptive behavior disorders (DBDs). Here, we report a GWAS meta-analysis of ADHD comorbid with DBDs (ADHD + DBDs) including 3802 cases and 31,305 controls. We identify three genome-wide significant loci on chromosomes 1, 7, and 11. A meta-analysis including a Chinese cohort supports that the locus on chromosome 11 is a strong risk locus for ADHD + DBDs across European and Chinese ancestries (rs7118422, P = 3.15×10^−10^, OR = 1.17). We find a higher SNP heritability for ADHD + DBDs (h^2^_SNP_ = 0.34) when compared to ADHD without DBDs (h^2^_SNP_ = 0.20), high genetic correlations between ADHD + DBDs and aggressive (r_g_ = 0.81) and anti-social behaviors (r_g_ = 0.82), and an increased burden (polygenic score) of variants associated with ADHD and aggression in ADHD + DBDs compared to ADHD without DBDs. Our results suggest an increased load of common risk variants in ADHD + DBDs compared to ADHD without DBDs, which in part can be explained by variants associated with aggressive behavior.

## Abstract (web summary)

ADHD is often found to be comorbid with disruptive behavior disorders, but the genetic loci underlying this comorbidity are unknown. Here, the authors have performed a GWAS meta-analysis of ADHD with disruptive behavior disorders, finding three genome-wide significant loci in Europeans, and replicating one in a Chinese cohort.

## Introduction

Attention-Deficit/Hyperactivity Disorder (ADHD) is a common childhood onset behavioral disorder affecting around 5% of children and 2.5% of adults^1^. Comorbidity with other psychiatric disorders is common among children with ADHD, and disruptive behavior disorders (DBDs) are the most frequently co-occurring conditions^2^. DBDs comprise oppositional defiant disorder and conduct disorder. Both have a childhood onset and are characterized by persistent patterns of oppositional, defiant, disobedient and disruptive behavior and antisocial rule-breaking, and aggressive behaviors such as being destructive, physically cruel towards others, and rule violations^3^. DBDs have a prevalence of 3–10%^4,5^ among children and, are around twice as frequent in males compared to females^6^ and DBDs are associated with a 3-fold increased risk of premature death^7^, with higher mortality rates than in ADHD^8^. Different comorbidity rates of ADHD with DBDs have been reported, some studies have found that approximately 30–40% of children with ADHD have comorbid DBDs (ADHD + DBDs)^9–11^, while a study of 1.92 million individuals from Denmark found that 17% of those with ADHD were also diagnosed with DBDs^8^. Among those with DBDs in the same Danish cohort, more than half (57%) had a comorbid diagnosis of ADHD^7^. ADHD in combination with diagnosed DBDs or excessive aggressive and disruptive behaviors increases the risk for several detrimental outcomes in individuals with ADHD including increased risks for substance use disorders^12–14^, in-patient psychiatric admission^15^, transgression^16,17^, risky behavior^18^, and premature death compared to those diagnosed with ADHD only^8^.

Both genetic and environmental factors influence the risk for ADHD and DBDs with twin heritability estimates of 0.74^19^ and 0.40–0.70^20–22^, respectively. Twin studies have also suggested that ADHD + DBDs is a more severe and genetically loaded subtype of ADHD than ADHD without comorbid DBDs^23^. Siblings of individuals with ADHD + DBDs have a higher recurrence risk to develop ADHD + DBDs compared to siblings of individuals having ADHD without DBDs (ADHDwoDBDs)^24,25^. Individuals with ADHD + DBDs also have an increased polygenic burden of common ADHD risk variants compared to individuals with only ADHD, further supporting the hypothesis that ADHD + DBDs reflect a higher load of genetic risk^26^. However, it seems unlikely that ADHD risk variants alone can fully account for the underlying genetic risk that mediates aggressive and disruptive behaviors in individuals with ADHD + DBDs. Family studies have found that ADHD and DBDs have distinct genetic architectures with moderate to high genetic overlap in the range of 0.34–0.74^27–29^. The existence of genetic risk factors specific to the aggressive and disruptive component of ADHD + DBDs finds support from a twin study, where DBDs had an estimated heritability of 0.33–0.64 after controlling for ADHD^30^.

Several genome-wide association studies (GWASs) have focused on diagnosed DBDs^31,32^ or aggressive and anti-social behaviors^33,34^, with only limited success in identifying genome-wide significant loci and no conclusive, replicated findings^31,33,34^. Only two genome-wide studies have focused specifically on ADHD + DBDs. One small genome-wide linkage study examined DBDs in individuals with ADHD^35^ and another, while not assessing diagnosed DBDs, examined aggressive behaviors in individuals with ADHD^36^. Neither studies reported genome-wide significant loci.

In the current study we perform a large GWAS meta-analysis of ADHD + DBDs using a Danish nation-wide cohort from iPSYCH and samples from the Psychiatric Genomics Consortium (PGC). We identify three genome-wide significant loci for ADHD + DBDs, located on chromosomes 1, 7, and 11, and show evidence of transancestral association for the locus on chromosome 11 in a Chinese cohort and, we find high polygenic overlap of ADHD + DBDs with childhood aggression and antisocial behavior in the general population, higher than found for ADHDwoDBDs.

## Results

### GWAS meta-analysis of ADHD + DBDs

**Fig. 1:**
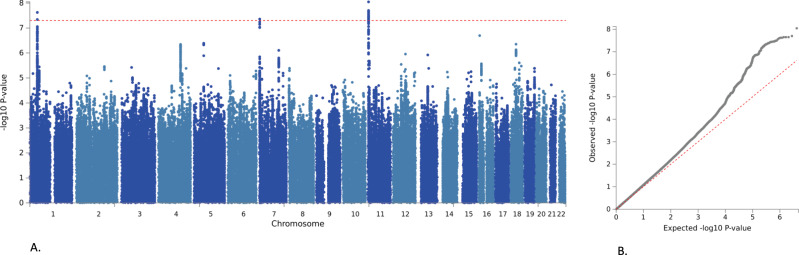
Manhattan plot and quantile–quantile plot of results from the GWAS meta-analysis of ADHD + DBDs. **A** Results from GWAS meta-analysis of iPSYCH and PGC cohorts in total including 3802 cases and 31,305 controls. The *x*-axis represents autosomal chromosomes colored in light and dark blue. The *y*-axis represents two-sided *P*-values from meta-analysis using an inverse-variance weighted fixed effects model. The red horizontal line represents the threshold for genome-wide significant association (*P* = 5 × 10^−8^). **B** Quantile–quantile (q–q) plot with expected −log10 *P*-values on the *x*-axis and −log10 *P*-values from the GWAS meta-analysis on the *y*-axis. The dotted line indicates the distribution under the null hypothesis.

The meta-analysis included data from the Danish iPSYCH cohort (2155 cases, 22,664 controls) and six European ancestry PGC cohorts (1647 cases, 8641 controls). All cases were diagnosed with both ADHD and DBDs or had a diagnosis of hyperkinetic conduct disorder, which according to the ICD10 criteria implies that both disorders are present. Selection of controls was population-based and they were not diagnosed with either ADHD or DBDs. Results were in total based on 3802 cases and 31,305 controls and included 8,285,688 variants after filtering. Three loci passed the threshold for genome-wide significance (*P* = 5 × 10^−8^); these were located on chromosome 1 (index variant rs549845, *P* = 2.38 × 10^−8^, OR = 1.16), 7 (index variant rs11982272, *P* = 4.38 × 10^−8^, OR = 0.83), and 11 (index variant, rs7118422, *P* = 8.97 × 10^−9^, OR = 1.16) (Table 1, Fig. 1a and Supplementary Fig. 1A–C). The directions of association of the index variants in the three loci were consistent across all cohorts (Supplementary Fig. 2A–C).

**Table 1:** Results for the genome-wide significant index variants in the three loci associated with ADHD + DBDs.

						iPSYCH+PGC				Chinese				iPSYCH+PGC + Chinese		
SNP	Chr	Gene	A1	A2		FRQ	OR	SE	*P*	FRQ	OR	SE	*P*	OR	SE	*P*
rs7118422	11	*STIM1*	T	C		0.51	1.164	0.026	8.97 × 10^−9^	0.48	1.276	0.089	0.006	1.173	0.025	3.15 × 10^−10^
rs549845	1	*PTPRF*	G	A		0.30	1.166	0.027	2.38 × 10^−8^	0.31	0.943	0.095	0.541	1.147	0.026	2.179 × 10^−7^
rs11982272	7	*MAD1L1*	T	C		0.78	1.199	0.033	4.39 × 10^−8^	0.86	0.901	0.132	0.132	1.179	0.032	3.013 × 10^−7^

Results from the GWAS meta-analysis of iPSYCH+PGC (European ancestry, 3802 cases and 31,305 controls). Results from GWAS of the Chinese cohort (406 cases and 917 controls) and the transancestry GWAS meta-analysis (4208 cases and 32,222 controls) for identification of cross-ethnicity risk variants in Europeans and Chinese (iPSYCH + PGC + Chinese). The location (chromosome (chr)), gene location of index variant (Gene), alleles (A1 and A2), frequency of A1(FRQ), odds ratio (OR) of the effect with respect to A1, and association *P*-values from inverse-variance weighted fixed effects model of the index variants are given.

### Homogeneity of effects in the PGC and iPSYCH cohorts and intercept evaluation

To evaluate the consistency of the genetic architecture underlying ADHD + DBDs in iPSYCH and PGC cohorts, we estimated the genetic correlation between the two using LD score regression^37,38^. The genetic correlation between the iPSYCH cohort and the meta-analyzed PGC cohorts was high (*r*_g_ = 0.934, SE = 0.14, *P* = 3.26 × 10^−11^) supporting consistency of the ADHD + DBDs phenotypes analyzed in the cohorts. In addition, no variants demonstrated significant heterogeneity between studies (Supplementary Figs. 3 and 4).

LD score regression analysis indicated that the observed deviation of the genome-wide test statistics from the null distribution (lambda = 1.11, Fig. 1b) was mainly caused by polygenicity. The intercept ratio estimate suggests that the majority of the inflation of the mean *χ*^2^ statistic of the GWAS meta-analysis is attributable to polygenic effects (ratio = 0.12, SE = 0.0662) rather than confounding factors. The estimated remaining contribution of confounding factors was small and non-significant (intercept = 1.015; SE = 0.008; *P* = 0.064).

### Transancestry GWAS meta-analysis across European and Han Chinese ancestry

**Fig. 2:**
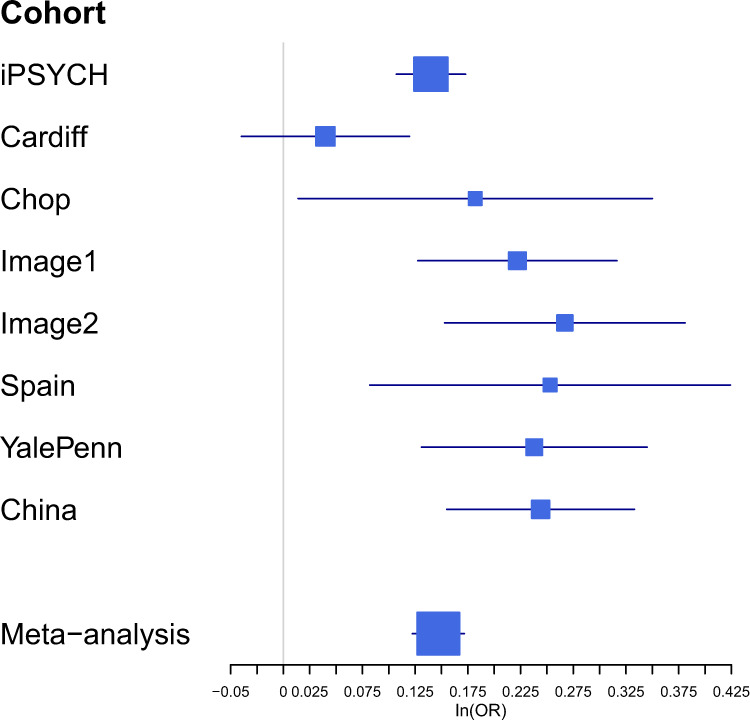
Forest plot for the index variant in the genome-wide significant locus for ADHD + DBDs on chromosome 11. Forest plot for the index variant (rs7118422) in the genome-wide significant locus on chromosome 11. Visualization of the effect size estimates (natural logarithm of the odds ratio (ln(OR)) in each included cohort, estimated from logistic regression, and in the trans-ancestry GWAS meta-analysis of European and Chinese cohorts (4208 cases and 32,222 controls) using an inverse-variance weighted fixed effects model. In addition, standard errors of the ln(OR) estimates. For information on sample sizes, allele frequencies and *P*-values for rs7118422 in the single cohorts see Supplementary Data 1.

To replicate and generalize the findings to other ethnicities, a GWAS of ADHD + DBDs was performed in a Han Chinese cohort (referred to as the Chinese cohort (406 cases, 917 controls; Supplementary Fig. 5). Of the three loci identified in the main analysis, the locus on chromosome 11 was nominally significant in the Chinese cohort (*P* = 0.006, Supplementary Fig. 8C). A fixed effects meta-analysis including the Chinese, European iPSYCH and PGC cohorts was performed in total including 4208 cases and 32,222 controls (no variants demonstrated significant heterogeneity across European and Chinese ancestries, Supplementary Figs. 6 and 7). For the locus on chromosome 11 the association *P*-value became stronger in the trans-ancestry GWAS meta-analysis (*P* = 3.15 × 10^−10^, OR = 1.17) (Fig. 2 and Supplementary Data 1, Supplementary Fig. 9), suggesting the locus is a risk locus for ADHD + DBDs across ethnicities. The results incorporating the Chinese cohort did not support replication of the other two loci (Table 1).

### Secondary GWASs

For subsequent evaluation of how much of the signal in the GWAS meta-analysis of ADHD + DBDs was driven by the oppositional/aggressive component of the comorbid phenotype, two additional GWASs were conducted using iPSYCH samples. To adjust for the effect of ADHD, we performed a case-only GWAS comparing 1959 individuals with ADHD + DBDs against 13,539 individuals having ADHDwoDBDs, referred to as the “ADHD + DBDs vs. ADHDwoDBDs GWAS”. Additionally, a GWAS of 13,583 cases having ADHDwoDBDs and 22,314 population-based controls, referred to as the “ADHDwoDBDs GWAS”, was performed (the case–control numbers differ between GWASs due to deviation in the numbers of related individuals and genetic outliers removed in the analyses).

The summary statistics from the two secondary GWASs were used to evaluate the direction of association of the top loci (281 loci, *P* < 1 × 10^−4^) from the GWAS meta-analysis of ADHD + DBDs. A consistent direction of association was observed for 221 loci (out of the 281 loci) in the ADHDwoDBDs GWAS (sign test *P* < 2.2 × 10^−16^), while all the 281 loci demonstrated consistent direction of association in the ADHD + DBDs vs. ADHDwoDBDs GWAS. The proportion of variants having a consistent direction of association in the ADHD + DBDs vs. ADHDwoDBDs GWAS was significantly larger than the proportion in the ADHDwoDBDs GWAS (*P* = 7.7 × 10^−16^), which suggests that the associations in the GWAS meta-analysis of ADHD + DBDs reflects association with the comorbid phenotype beyond association with risk for ADHD alone.

All three genome-wide significant loci demonstrated higher effect sizes in ADHD + DBDs compared to ADHDwoDBDs (Supplementary Data 2). In particular, the effect sizes for the loci located on chromosomes 7 and 11 (OR_chr7_ = ORs 1.199; OR_chr11_ = 1.164) remained strong in the ADHD + DBDs vs. ADHDwoDBDs GWAS (OR_chr7_ 1.128; OR_chr11_ = 1.126) confirming a stronger effect of the risk allele for these two loci in ADHD + DBDs compared to ADHDwoDBDs (Supplementary Data 2). The difference was most striking for rs7118422 on chromosome 11 (ADHD + DBDs: OR = 1.164, *P* = 8.97 × 10^−9^), which showed no evidence for association with ADHDwoDBDs (OR = 1.022, *P* = 0.175) versus a suggestive evidence of association with ADHD + DBDs vs. ADHDwoDBDs (OR = 1.126, *P* = 7.07 × 10^−04^).

To help formalize the comparison of ADHD + DBDs with ADHDwoDBDs, we also used mtCOJO^39^ to estimate the joint effects of the significant loci from the GWAS of ADHD + DBDs conditional on effects mediated through the genetics of ADHDwoDBDs. Under this model (see “Methods” section) none of the three loci reached genome-wide significance for a direct effect on ADHD + DBDs, although the locus on chromosome 11 retained the most robust signal after correction for ADHDwoDBDs (OR_adjusted_ = 1.14; *P*_adjusted_ = 1.43 × 10^−06^, Supplementary Data 3).

### Gene-based association test

A gene-based association analysis was performed using MAGMA^40^. Six genes (*RRM1*, *STIM1, MAML3*, *ST3GAL3*, *KDM4A*, and *PTPRF*) were significantly associated with ADHD + DBDs (*P* < 2.7 × 10^−6^ correcting for 18,553 genes analyzed; Supplementary Fig. 10 and Supplementary Data 4). Three genes (*ST3GAL3*, *KDM4A*, and *PTPRF)* are located in the genome-wide significant locus on chromosome 1, and two genes (*RRM1*, *STIM1*) in the genome-wide significant locus on chromosome 11. One gene *(MAML3)* located on chromosome 4 had not been identified as a risk locus in the single variant analysis.

To evaluate if the gene-based association signals reflected the aggressive and disruptive component of the ADHD + DBDs phenotype rather than ADHD alone, we did a gene-set test of the most associated genes from the primary ADHD + DBDs GWAS meta-analysis (*P* < 10^−3^, 79 genes) using the results from the two secondary GWASs. The gene set was significantly associated with ADHDwoDBDs (beta = 0.312 (SE = 0.02), *P* = 9 × 10^−4^), but had a stronger association in the ADHD + DBD vs. ADHD only GWAS (beta = 1.1 (SE = 0.07), *P* = 9.28 × 10^−32^).

### Association of genetically regulated gene expression with ADHD + DBDs

Association of the genetically regulated gene expression with ADHD + DBDs was analyzed in 12 brain tissues from GTEx^41^ (version 6p) using MetaXcan^42^. Depending on the tissue, 2042–6094 genes were tested (Supplementary Data 5). Three genes were predicted to be differently expressed in ADHD + DBDs cases compared with controls after Bonferroni correction (correcting for the total number of tests performed (43,142); *P* < 1.16 × 10^−6^); *RRM1* (chromosome 11) was less expressed in cases, while *RAB3C* (chromosome 5) and *LEPRE1* (chromosome 1) showed a higher expression in cases when compared to controls (Supplementary Data 5). The genes on chromosome 1 and 11 were located in or near genome-wide significant loci, whereas the gene on chromosome 5 was novel.

### SNP heritability

SNP heritability (*h*^2^_SNP_) was estimated using a prevalence of ADHD + DBDs in the population of 1–2%, based on studies reporting that around 20–40% of individuals with ADHD have comorbid DBDs^8–11^. The estimated *h*^2^_SNP_ for ADHD + DBDs was 0.25 (SE = 0.03) using LD score regression^37^ with a prevalence estimate of 2% in the population (Supplementary Data 6A). When considering only the iPSYCH cohort, a higher SNP heritability was found for ADHD + DBDs (*h*^2^_SNP_ = 0.34; SE = 0.05) compared to ADHDwoDBDs (*h*^2^_SNP_ = 0.20; SE = 0.02). This pattern remained similar when using GCTA and was stable when assuming a lower prevalence of ADHD + DBDs (1%) and higher prevalence of ADHDwoDBDs (3%) (Supplementary Data 6A). The *h*^2^_SNP_ estimate was significantly higher for ADHD + DBDs compared to ADHDwoDBDs in GCTA analyses of independent iPSYCH samples, with *P*_difference_ = 5.36 × 10^−6^ when assuming a prevalence of 2% for ADHD + DBDs and 3% for ADHDwoDBDs (Supplementary Data 6B). Additionally, common variants explained a small fraction of the variation in the ADHD + DBDs phenotype compared to the ADHDwoDBDs phenotype (GCTA *h*^2^_SNP_ = 0.08; SE = 0.04; Supplementary Data 6A).

### Genetic correlation with aggression-related phenotypes

We estimated the genetic correlations of ADHD + DBDs with aggression-related phenotypes using GWAS results from analyses of aggressive behaviors in 18,988 children^33^ (EAGLE aggression) and antisocial behavior in 16,400 individuals^34^ (Broad Antisocial Behavior Consortium (BroadABC)) using LD score regression^37^. We found a high genetic correlation of ADHD + DBDs with aggression in children (EAGLE aggression, *r*_g_ = 0.81; SE = 0.24; *P* = 0.001) and antisocial behavior (BroadABC, *r*_g_ = 0.82; SE = 0.30; *P* = 0.007) (Supplementary Data 7). In contrast, ADHDwoDBDs (analysed solely in iPSYCH) was only significantly correlated with aggression in children (*r*_g_ = 0.74; SE = 0.18; *P* = 4.6 × 10^−5^). Analyzing only the iPSYCH cohort, ADHD + DBDs demonstrated a nominally higher positive genetic correlation than ADHDwoDBDs with aggression in children (*r*_g_ = 0.85; SE = 0.24; *P* = 5 × 10^−4^; and *r*_g_ = 0.74; SE = 0.18; *P* = 4.58 × 10^−5^, respectively) and antisocial behavior (*r*_g_ = 0.92; SE = 0.35; *P* = 9 × 10^−3^; and *r*_g_ = 0.56; SE = 0.22; *P* = 0.01, respectively). The differences in the genetic correlations, however, were not statistically significant when assessed using the jackknife method^43^. Finally, we estimated the genetic correlation of ADHD + DBDs vs ADHDwoDBDs (*r*_g_ = 0.99, SE = 0.07, *P* = 2.64 × 10^−45^).

### Polygenic score analysis of ADHD + DBDs compared to ADHDwoDBDs

Case-only polygenic score (PGS) analyses were done to evaluate whether ADHD + DBDs cases are enriched for variants associated with 22 relevant phenotypes related to personality, cognition, and psychiatric disorders compared with cases having ADHDwoDBDs. Seven phenotypes were significantly associated with ADHD + DBDs compared with ADHDwoDBDs after multiple testing correction (Supplementary Data 8). Significantly increased PGS for aggressive behavior^33^ (*Z* = 4.80, *P* = 1.51 × 10^−6^, OR = 1.13) and ADHD (*Z* = 5.42, *P* = 5.90 × 10^−8^, OR = 1.21) were observed for ADHD + DBDs. Additionally, PGS for increased cognitive performance was negatively associated with ADHD + DBDs compared to ADHDwoDBDs (educational attainment^44^, *Z* = −3.30, *P* = 8.0 × 10^−4^, OR = 0.92; college or university degree^45^ *Z* = −3.22, *P* = 1.0 × 10^−3^, OR = 0.93; human intelligence^46^ *Z* = −3.00, *P* = 2.00 × 10^−3^, OR = 0.93; verbal–numerical reasoning^45^ *Z* = −3.26, *P* = 1.00 × 10^−3^, OR = 0.92). Finally, PGS for having children at an older age was negatively associated with ADHD + DBDs compared to ADHDwoDBDs (*Z* = −4.40, *P* = 8.4 × 10^−6^, OR = 0.9). Only a small proportion of the variance in ADHD + DBDs among individuals with ADHD was explained by the PGSs, with the maximum Nagelkerke’s *R*^2^ = 0.36% for the ADHD PGS. The odds ratio for ADHD + DBDs was increasing across quintiles of the polygenic load of variants associated with aggression, and ADHD, and decreasing with higher load of variants associated with cognition and age at first birth (Supplementary Fig. 11A–G). The highest risk was observed for ADHD PGS, where the 20% of ADHD cases with the highest ADHD PGS had an OR = 2.48 for having comorbid DBDs relative to the 20% with the lowest ADHD PGS (Supplementary Figure 11A).

## Discussion

This study identifies genome-wide significant loci for ADHD + DBDs based on a meta-analysis of 3802 cases and 31,305 controls from the iPSYCH cohort and six cohorts from PGC. We identified three risk loci on chromosomes 1, 7, and 11 with odds ratios ranging from 1.16 to 1.20, in line with what was found in the recent GWAS meta-analysis of ADHD^47^. These risk loci demonstrated high consistency in the direction of association in the included cohorts, indicating that the associations likely have a biological cause rather than being spurious signals driven by one or few cohorts (Fig. 2 and Supplementary Fig. 2A–C). The high genetic correlation observed between the PGC cohorts and the iPSYCH cohort suggests that the genetic architecture underlying ADHD + DBDs were similar in the two samples. In the GWAS meta-analysis for trans-ancestry risk of the identified loci in a Chinese sample, only the locus on chromosome 11 replicated the findings in the European samples. This locus seems to be specifically associated with the aggressive and disruptive component of the ADHD + DBDs phenotype, since the effect disappeared in the ADHDwoDBDs GWAS. This was further supported in the GWAS comparing comorbid ADHD + DBD to ADHDwoDBDs where the locus remained strongly associated, although not genome-wide significant (Supplementary Data 2). Consistent with this, evidence for a direct effect of the locus on ADHD + DBDs remained after adjusting for the effect of ADHDwoDBDs in the mtCOJO analysis (Supplementary Data 3).

In contrast, the locus on chromosome 1, which was previously identified as a strong risk locus for ADHD^47^, seems to reflect an association with ADHD. This locus remained genome-wide significant in the GWAS of ADHDwoDBDs (Supplementary Data 2) and the association with ADHD + DBDs decreased considerably in the analyses adjusting for the effect of ADHDwoDBDs and in the ADHD + DBDs vs. ADHDwoDBDs GWAS (Supplementary Data 2 and 3).

The locus on chromosome 7 seems to be a shared risk locus between ADHD + DBDs and ADHDwoDBDs. The locus remained moderately associated in the GWASs adjusted for ADHD (Supplementary Data 2 and 3) as well as in the ADHDwoDBDs GWAS (Supplementary Data 2). The locus is located in *MAD1L1*, which encodes a protein involved in mitotic spindle-assembly checking before anaphase. The locus is novel with respect to ADHD and DBDs, but was found genome-wide significant in the recent large cross-disorder GWAS^48^ and has previously been associated with schizophrenia and bipolar disorder^49–51^, suggesting that *MAD1L1* is a risk gene for several psychiatric disorders.

The locus most strongly associated with ADHD + DBDs on chromosome 11 is located in *STIM1* (Supplementary Fig. 1C), a gene not previously implicated in ADHD, DBDs, aggression-related phenotypes, or psychiatric disorders. *STIM1* encodes a transmembrane protein (STIM1) in the endoplasmatic reticulum (ER) that acts as a sensor of calcium. Upon calcium depletion from the ER, STIM1 is responsible for an influx of calcium ions from the extracellular space through store-operated calcium channels to refill ER stores^52–54^. Store-operated calcium entry may also be involved in neuronal calcium signaling^55^, and recent evidence indicates that STIM1 plays a role in synaptic plasticity affecting learning and memory^55,56^. These results are interesting in the light of the observed learning deficits associated with aggressive behaviors and accumulating evidence that suggests calcium signaling is involved in several psychiatric disorders^57–59^. Alternatively, analysis of genetically regulated gene expression suggested that the variants in the genome-wide significant locus might affect expression of *RRM1*, with a decreased *RRM1* expression being associated with ADHD + DBDs. *RRM1* is oriented in a tail-to-head configuration with *STIM1*, which lies 1.6 kb apart, and encodes a subunit of a reductase involved in the biosynthesis of deoxyribonucleotides from the corresponding ribonucleotides necessary for DNA replication. To our knowledge, this gene has not previously been associated with psychiatric disorders.

In the gene-based analysis six genes were exome-wide significantly associated with ADHD + DBDs, including two implicated by variants in or near the genome-wide significant locus on chromosome 11 (*RRM1* and *STIM1*) and with three (*ST3GAL3*, *KDM4A*, and *PTPRF*) out of the remaining four located in or near the genome-wide significant locus on chromosome 1. The top-associated genes (79 genes) seem to mainly reflect association with the aggressive and disruptive component of the ADHD + DBDs phenotype. The geneset was significantly associated with ADHDwoDBDs but even more strongly associated in the ADHD + DBDs vs. ADHDwoDBDs GWAS (where the effect of ADHD is corrected out). Likewise, the most strongly associated single markers (with *P* < 1 × 10^−4^) in the GWAS meta-analysis of ADHD + DBDs showed high consistency in the direction of association in the GWAS of ADHDwoDBDs, but even higher consistency in the ADHD + DBDs vs. ADHDwoDBDs GWAS, reinforcing the notion that the associations mainly reflect the aggressive and disruptive component of the phenotype.

When evaluating the polygenic architecture of ADHD + DBDs, a higher SNP heritability was found for ADHD + DBDs (*h*^2^_SNP_ = 0.34; SE = 0.05) compared to ADHDwoDBDs (*h*^2^_SNP_ = 0.2; SE = 0.02) (Supplementary Data 6A and 6B). These estimates are consistent with the recently reported SNP heritability of ADHD (*h*^2^_SNP_ = 0.22; SE = 0.01)^47^, which included individuals with and without comorbid DBDs. Conditional on an ADHD diagnosis, the aggressive and disruptive behavioral component of the ADHD + DBDs phenotype also has a genetic component involving common variants (*h*^2^_SNP_ = 0.08; SE = 0.04). PGS analyses suggested the higher SNP heritability of ADHD + DBDs compared to ADHDwoDBDs is partly explained by a higher burden of common ADHD risk variants (Supplementary Data 8). The significantly higher burden of ADHD risk variants among individuals with ADHD + DBDs was especially evident when examining individuals belonging to the 20% of ADHD cases with the highest ADHD genetic risk load, who had an odds ratio of 2.48 for having comorbid DBDs (Supplementary Fig. 11A). This is also a replication of previous findings of a higher load of ADHD risk variants in individuals with ADHD and comorbid conduct disorder compared to those having only ADHD^26^.

Going beyond ADHD risk burden, the common variant component of ADHD + DBDs could also include variants mainly associated with aggression. This idea is supported by our finding of increased PGS for aggression in ADHD + DBDs compared to ADHDwoDBDs (Supplementary Fig. 11B). This conclusion is reinforced by the genetic correlation results, where we found somewhat higher genetic correlation of ADHD + DBDs with both aggressive behavior in children^33^ and antisocial behavior^34^ (Supplementary Data 7) compared to those found for ADHDwoDBDs. Additionally, these results imply that the genetic architecture underlying the aggressive and disruptive behavioral component of the ADHD + DBDs phenotype overlaps strongly with that affecting aggressive and antisocial behavior in the general population. Thus, aggressive and antisocial behaviors seem to have a continuous distribution in the population, with individuals having ADHD + DBDs representing an extreme. This is in line with what has been observed for other complex phenotypes, such as diagnosed ADHD representing the upper tail of impulsive and inattention behaviors^47^, and diagnosed autism spectrum disorder representing the upper tail for social communication difficulties and rigidity^60,61^.

Aggressive behavior is stable across age intervals during childhood^62^, and twin studies have suggested genetics play an important role in this stability^62,63^. Moreover, early aggression might be predictive of later serious antisocial behavior^64^ resulting in increased risk of a diagnosis of antisocial personality disorder^65^. Our results suggest that common genetic variants play an important role in childhood aggression, which has also been reported previously^33^, and that the subsequent risk for antisocial behavior in individuals with ADHD + DBDs to some extent has an underlying genetic cause involving common variants. However, our results do not reveal whether the increased polygenic load of variants associated with aggression observed in ADHD + DBDs is caused by variants specific to DBDs or due to a general increased load of variants that are also shared with ADHD.

It should also be noted that the SNP heritabilities of childhood aggression^33^ and antisocial behavior^34^ are at the low end (0.05 and 0.06, respectively), and probably biased downwards by heterogeneity in the cohorts analyzed^33^. The studies do therefore not capture the full impact of common variants in aggressive behavior, and the observed high genetic correlation of the two phenotypes with ADHD + DBDs involves variants that only explain a small proportion of variance in aggression.

We have shown that individuals with ADHD + DBDs have an increased load of variants associated with worse cognition compared to individuals having ADHDwoDBDs (Supplementary Data 8 and Supplementary Fig. 11C–F). This could reflect the increased genetic load of ADHD risk variants in ADHD + DBDs, since ADHD has a strong negative genetic correlation with cognition-related phenotypes^47^. However, it might also involve variants associated with antisocial behavior, which also has a negative genetic correlation with educational performance^34^. This latter idea is consistent with epidemiological studies linking aggression to decreased educational attainment^66–68^. Finally, we found a significantly higher load of variants associated with younger age at birth of first child in ADHD + DBDs compared to ADHDwoDBDs, in line with the observed positive genetic correlations of ADHD and antisocial behavior with having children earlier^34,47^ and evolutionary theories suggesting that aggression has played a role when competing for access to mates^69^.

In summary, we identified three genome-wide significant loci for ADHD + DBDs. The locus on chromosome 11 was associated most strongly with the comorbid phenotype, and seems to be a cross-ancestry risk locus in Europeans and Chinese. Our results suggest that the aggressive and disruptive behavioral component of the ADHD + DBDs phenotype has a genetic risk component, which in part include common risk variants associated with ADHD, aggressive, and antisocial behavior. Individuals with ADHD + DBDs therefore represent a phenotype with an increased genetic risk load compared to ADHDwoDBDs, including at least one genome-wide significant locus specific to ADHD + DBDs. This study represents the first step towards a better understanding of the biological mechanism underlying ADHD + DBDs.

## Methods

### Samples—the iPSYCH cohort

The iPSYCH cohort is a population-based nation-wide cohort which includes 79,492 genotyped individuals (∼50,000 diagnosed with major psychiatric disorders and ∼30,000 controls). The cohort was selected, based on register information from a baseline birth cohort of all singletons born in Denmark between May 1st, 1981 and December 31, 2005 (*N* = 1,472,762) (see a detailed description in the ref. ^70^). A biological sample of the included individuals were obtained from the Newborn Screening Biobank at Statens Serum Institute, Denmark. DNA was extracted from dried blood spot samples and whole genome amplified in triplicates^71,72^. Genotyping and calling of genotypes were performed as described in our previous publications^47,70^.

For this study cases and controls were identified based on diagnoses given in 2016 or earlier in the Danish Psychiatric Central Research Register^73^. Cases with ADHD + DBDs had a diagnosis of hyperkinetic conduct disorder (F90.1) or an ADHD diagnosis (ICD-10 F90.0) occurring together with a diagnosis of ODD (ICD-10 F91.3) or conduct disorder (ICD-10 F91.0, F91.1, F91.2, F91.8, and F91.9). Distribution of cases with ADHD + DBDs over diagnosis codes is presented in Supplementary Data 9. ADHD cases without DBDs were defined as individuals having ADHD (ICD-10 F90.0) without any diagnosis of DBDs. Controls were randomly selected from the same nation-wide birth cohort and not diagnosed with ADHD or DBDs.

The study was approved by the Danish Data Protection Agency and the Scientific Ethics Committee in Denmark. All analyses of the iPSYCH cohort were performed at the secured national high performance-computing cluster, GenomeDK (https://genome.au.dk).

### Samples—cohorts from the Psychiatric Genomics Consortium

For the meta-analysis, seven ADHD cohorts (six cohorts of European ancestry and one of Chinese ancestry) provided by PGC with information about diagnoses of ADHD + DBDs were included. An overview of the cohorts including genotyping information and diagnosis criteria can be found in Supplementary Data 10. Detailed descriptions of the cohorts can be found elsewhere^74^. Details on approval authorities can be found in Supplementary Data 10.

### Quality control and imputation

Quality control, imputation, and primary GWASs of the iPSYCH and PGC cohorts (including the Chinese cohort) were done separately for each using the bioinformatics pipeline Ricopili^75^. Pre-imputation quality control allowed an inclusion of individuals with a call rate > 0.98 (>0.95 for iPSYCH) and genotypes with a call rate >0.98, difference in SNP missingness between cases and controls < 0.02, no strong deviation from Hardy–Weinberg equilibrium (*P* > 1 × 10^−6^ in controls or *P* > 1 × 10^−10^ in cases) and low individual heterozygosity rates (|*F*_het_ | < 0.2). Genotypes were phased and imputed using SHAPEIT^76^ and IMPUTE2^77^ and the 1000 Genomes Project phase 3 (1KGP3)^78^ as imputation reference panel (the East Asian reference genome was used for imputation of the Chinese sampels). Trio imputation was done with a case-pseudocontrol setup.

Relatedness and population stratification were evaluated using a set of high-quality genotyped markers (minor allele frequency (MAF) > 0.05, HWE *P* > 1 × 10^−4^ and SNP call rate >0.98) pruned for linkage disequilibrium (LD) resulting in ~30,000 pruned variants (variants located in long-range LD regions defined by Price et al.^79^ were excluded). Genetic relatedness was estimated using PLINK v1.9^80,81^ to identify first and second-degree relatives ($\hat \pi$ > 0.2) and one individual was excluded from each related pair (cases preferentially retained over controls). Genetic outliers were excluded based on principal component analyses (PCA) using EIGENSOFT^82,83^. For iPSYCH a genetic homogenous sample was defined based on a subsample of individuals being Danes for three generations as described in Demontis and Walters et al.^47^. For the PGC samples genetic outliers were removed based on visual inspection of the first six PCs. For all cohorts PCA was redone after exclusion of genetic outliers.

### GWAS meta-analysis and transancestry risk in European and Chinese ethnicities

Association analysis was done in PLINK^80^ using additive logistic regression and the imputed marker dosages, covariates from principal component analyses (after removal of genetic outliers) and other relevant covariates (Supplementary Data 10). Meta-analysis of the iPSYCH cohort (2155 cases, 22,664 controls) and the six PGC cohorts (1647 cases, 8641 controls) was done using an inverse standard error weighted fixed effects model and the software METAL^84^ and included in total 3802 cases and 31,305 controls.

For transancestry genetic risk variants in European and Chinese cohorts, a GWAS meta-analysis was done as described above including the iPSYCH cohort, the six European PGC cohorts and the cohort of Chinese ancestry. In total, 4208 cases and 32,222 controls were included. No individual genotypes were used for the meta-analysis.

In the two meta-analyses only variants with MAF > 0.01 and imputation INFO score > 0.8 were included. All variants that were not supported by an effective sample size of 70% in the meta-analysis output were filtered out.

### Homogeneity of effects in the PGC and iPSYCH cohorts and intercept evaluation

LD score regression^37,38^ was used to estimate the genetic correlation using summary statistics from GWAS of ADHD + DBDs in the iPSYCH cohort and meta-analysis of the six European PGC cohorts. Only variants with an imputation info score > 0.9 were included. The intercept was restricted to one as there was no sample overlap and no indication of population stratification.

The ratio (ratio = (intercept−1)/(mean *χ*^2^ − 1)) from LD score regression was used to evaluate the relative contribution of polygenic effects and confounding factors to the observed deviation from the null in the genome-wide distribution of the *χ*^2^ statistics of the GWAS meta-analysis of ADHD + DBDs.

### Secondary GWASs

In order to adjust for the effect of ADHD we did a case-only GWAS comparing 1,959 individuals having ADHD + DBDs against 13,539 individuals having ADHDwoDBDs, referred to as the “ADHD + DBDs vs. ADHDwoDBDs GWAS”. Additionally, a GWAS of 13,583 cases having ADHDwoDBDs and 22,314 population-based controls referred to as the “ADHDwoDBDs GWAS” was performed. Both GWASs were based only on iPSYCH samples and performed using additive logistic regression and the imputed marker dosages, covariates from principal component analyses (after removal of genetic outliers) and covariates indicating genotyping waves.

The summary statistics from the two secondary GWASs were used to evaluate direction of association of the top loci associated with ADHD + DBDs using a sign test based on LD distinct variants (*r*^2^ < 0.2, 281 variants) with association *P*-values less than 1 × 10^−4^ in the GWAS meta-analysis of ADHD + DBDs.

We also did an mtCOJO^39^ analysis to estimate the effect of the top loci for ADHD + DBDs conditional on genetic effects on ADHD alone. This was done using summary statistics from the GWAS meta-analysis of ADHD + DBDs and from the GWAS of ADHDwoDBDs. The analysis was run using mtCOJO^39^ implemented in GCTA^85^ using standard procedures. Following default settings, estimation of the effect of ADHDwoDBDs on ADHD + DBDs (as part of the indirect path contributing to marginal ADHD + DBDs associations) was performed using variants that were genome-wide significant in the GWAS of ADHDwoDBDs (*P* < 5 × 10^−8^), and not in linkage disequilibrium (*r*^2^ < 0.05; 7 index variants). No variants were removed due to evidence of pleiotropy (HEIDI-outlier threshold of *P* = 0.01).

### Gene-based association test

Gene-based association analysis was done using MAGMA 1.05^40^ and summary statistics from the GWAS meta-analysis. Variants were annotated to genes using the NCBI37.3 gene definitions and no window around genes was used. MAGMA summarizes association signals observed for variants located in a gene into a single *P*-value while correcting for LD in a reference genome. For this the European samples from the 1000 Genomes phase 3 were used.

The most associated genes in the GWAS meta-analysis of ADHD + DBDs (79 genes, *P* < 10^−3^ Supplementary Data 4) were evaluated in a gene-set test for association with ADHD + DBDs compared to ADHDwoDBDs and for association with ADHDwoDBDs. Gene-based *P*-values were generated using summary statistics from the two secondary GWASs (ADHD + DBDs vs. ADHD-only GWAS and ADHDwoDBDs GWAS) and subsequently gene-set tests were done using MAGMA 1.05^40^. MAGMA performs a competitive test to analyze if the gene set is more strongly associated with the phenotype than other genes, while correcting for a series of confounding effects such as gene length and size of the gene set.

### Association of the genetically regulated gene expression with ADHD + DBDs

Association of the genetically regulated gene expression with ADHD + DBDs was analyzed in 12 brain tissues from GTEx^86^ (version 6p) using MetaXcan^42^ imlemented in the R-package metaxcanr (https://github.com/drveera/metaxcanr). MetaXcan is an extension of PrediXcan^87^ that can be used to test for differences in gene expression using summary statistics. We used high-performance prediction models for MetaXcan based on variants located within 1 Mb +/− of transcription start site and trained using elastic net regression and 10-fold cross-validation^4^ downloaded from http://predictdb.org. MetaXcan also requires covariance matrices of the variants within each gene model for each tissue. Covariance matrices calculated from 503 individuals with European ancestry from the 1000 genomes project^88^ available with the prediction models at http://predictdb.org were used.

### SNP heritability

The SNP heritability (*h*^2^_SNP_) was estimated using LD score regression^37^ and the summary statistics from the GWAS meta-analysis of ADHD + DBDs. The heritability was estimated on the liability scale assuming a population prevalence of ADHD + DBDs of 2 and 1%.

In order to evaluate the extent to which common genetic variants contributes to the risk of ADHD + DBDs compared to having ADHDwoDBDs, the SNP heritability of for the two phenotypes were estimated only in iPSYCH samples. This was done using LD score regression and univariate GREML analyses in GCTA^85^. *h*^2^_SNP_ was estimated on the liability scale assuming a population prevalence of 2 and 1% for ADHD + DBDs and 3 and 4% for ADHDwoDBDs. The GCTA analyses were corrected for the same covariates as used in the GWASs.

In order to be able to test for difference in *h*^2^_SNP_ between ADHD + DBDs and ADHDwoDBDs we re-estimated *h*^2^_SNP_ using GCTA based on independent iPSYCH controls. For this analysis the iPSYCH controls were split randomly into two groups within each genotyping wave. One group was used as controls for estimating *h*^2^_SNP_ of ADHD + DBDs (2155 cases and 11,659 controls) and the other group was used to estimate *h*^2^_SNP_ of ADHDwoDBDs (13,583 cases and 11,250 controls). The analysis using independent controls was done using prevalances of 2 and 1% for ADHD + DBDs and 3 and 4% for ADHDwoDBDs. Test for difference in *h*^2^_SNP_ between ADHD + DBDs and ADHDwoDBDs was done using Eq. 1 below:

1 $${{Z}}_{{\mathrm{diff}}} = \left( {{{h}}^2_{{\mathrm{SNP}}\left( {{\mathrm{ADHD}} + {\mathrm{DBDs}}} \right)} - {\mathrm{h}}^2_{{\mathrm{SNP}}\left( {{\mathrm{ADHDwoDBDs}}} \right)}/{\mathrm{sqrt}}\left( {{\mathrm{SE}}^2_{\left( {{\mathrm{ADHD}} + {\mathrm{DBDs}}} \right)} + {\mathrm{SE}}^2_{\left( {{\mathrm{ADHDwoDBDs}}} \right)}} \right.} \right),$$

where *Z*_diff_ is the *Z*-score for the difference in *h*^2^_SNP_ and SE is the standard errors for the heritabilities. We calculated two-tailed *P*-values in R.

Additionally, we evaluated how much of the variance in the ADHD + DBDs phenotype could be explained by common genetic variation in the context of ADHD. For this we did a case-only approach including 1959 cases with ADHD + DBDs and 13,539 individuals with ADHDwoDBDs. This was only done using GCTA due to a low polygenic signal in the ADHD + DBDs vs. ADHDwoDBDs GWAS (mean *χ*^2^ = 1.06).

### Genetic correlation with aggression-related phenotypes

The genetic overlap of ADHD + DBDs with aggression-related phenotypes was evaluated by estimating genetic correlations using LD score regression^37^ and the summary statistics from the GWAS meta-analysis of ADHD + DBDs and results from two aggression related GWASs. One is a GWAS meta-analysis of scores of aggressive behaviors in 18,988 children^33^ (EAGLE aggression) obtained by questionnaires filled by their parents. Another is a GWAS meta-analysis of antisocial behavior conducted by the Broad Antisocial Behavior Consortium (BroadABC) including 16,400 individuals^34^. Both children and adults were accessed for a broad range of antisocial measures, including aggressive and non-aggressive domains. The BroadABC study has a minor overlap with the EAGLE aggression GWAS with respect to the included cohorts. We also estimated the genetic correlations of ADHDwoDBDs and ADHD+DBDs (only including iPSYCH individuals) with the two aggression-related phenotypes.

The genetic correlation between ADHD + DBDs and ADHDwoDBDs was calculated using LD score regression and summary statistics from the GWAS meta-analysis of ADHD + DBDs and the GWAS of ADHDwoDBDs, the latter based on iPSYCH data only. To supplement the LD score regression analysis we used iPSYCH genotypes and GCTA to estimate the genetic correlation between ADHD + DBDs (2155 cases and 11,659 controls) and ADHDwoDBDs (13,583 cases and 11,250 controls), showing results consistent with LD score regression (*r*_g_ = 0.97; SE = 0.06), and not statistically different from one.

Statistical difference between two *r*_g_ estimates was calculated using the block jackknife method^43^ implemented in the LD score regression software^37,89^. The variants across the genome were divided in 200 blocks and jackknife deleted values were calculated by excluding one block at a time. The computed jackknife deleted values were then used to calculate corresponding jackknife pseudo values. By using the mean and variance of the jackknife pseudovalues, *Z*-score and corresponding *P*-values were computed, testing the null hypothesis that the difference between the *r*_g_s is equal to zero.

### Polygenic score analysis

Case-only polygenic score (PGS) analyses were done using GWAS summary statistics from 22 GWASs related to cognition and education (six phenotypes), personality (nine phenotypes), psychiatric disorders (five phenotypes), reproduction/fitness (two phenotypes) (detailed list of phenotypes see Supplementary Data 3). Variants with imputation info score < 0.9, MAF < 0.01, missing values, ambiguous and multiallelic variants, indels and duplicated identifiers were removed. The remaining variants were LD-clumped using Plink^80^. PGS was estimated at different *P*-value thresholds in the 22 training datasets: *P* < 5 × 10^−8^, 1 × 10^−6^, 1 × 10^−4^, 1 × 10^−3^, 0.01, 0.05, 0.1, 0.2, 0.5, and 1.0. PGS in the target individuals (iPSYCH samples: 1959 ADHD + DBDs cases and 13,539 ADHDwoDBDs cases) were estimated multiplying the natural log of the odds ratio of each variant by the allele-dosage of each variant and whole-genome PGS was obtained by summing values over variants for each individual. The ADHD PGS was generated using the approach described in Demontis et al.^47^: in short ADHD cases and controls in the iPSYCH cohort were split into five independent samples, then one sample was left out in an ADHD GWAS meta-analysis of iPSYCH data and data from the Psychiatric Genetics Consortium. The results were then used as training for generating PGS in the sample that was left out. This “leave-one sample out” procedure was repeated until ADHD PGSs were generated for all individuals in the iPSYCH cohort. For each *P*-value threshold the variance in the ADHD + DBDs phenotype explained by PGS was estimated using Nagelkerke’s *R*^*2*^ (R package “BaylorEdPsych”) and conversion to Nagelkerke’s *R*^2^ on the liability scale was done using the method suggested by Lee et al.^90^ and a prevalence of 0.2 for comorbid DBDs among ADHD cases. Association of PGS with ADHD + DBDs compared to ADHDwoDBDs was estimated using logistic regression including the same covariates used in the GWAS.

Subsequently individuals were divided into quintiles based on their PGS. OR for ADHD + DBDs compared to ADHDwoDBDs was estimated within each quintile with reference to the lowest risk quintile (using the training data *P*-value threshold resulting in the highest Nagelkerke’s *R*^2^ in the target data).

### Reporting summary

Further information on research design is available in the Nature Research Reporting Summary linked to this article.

## Supplementary information

Supplementary Information

Peer Review

Reporting Summary

Description of Additional Supplementary Files

Supplementary Data 1-10

## Data Availability

All relevant iPSYCH data are available from the authors after approval by the iPSYCH Data Access Committee and can only be accessed on the secured Danish server as the data are protected by Danish legislation. Access to data provided by the Psychiatric Genomics Consortium can be granted through the Psychiatric Genomics Data Access Committee https://www.med.unc.edu/pgc/about-us/people/data-access-committee/”. For data access please contact: Ditte Demontis, email: ditte@biomed.au.dk, Anders D. Børglum, email: anders@biomed.au.dk. The summary statistics with the results from the GWAS meta-analysis of ADHD + DBDs are available on the iPSYCH website (https://ipsych.dk/en/research/downloads/).

## Code availability

MetaXcan analysis was performed using the R-package metaxcanr (https://github.com/drveera/metaxcanr), and GTEx v6p expression prediction models and covariance matrices downloaded from http://predictdb.org.
