# Author Correction: Risk variants and polygenic architecture of disruptive behavior disorders in the context of attention-deficit/hyperactivity disorder

**DOI:** 10.1038/s41467-021-21566-w

**Published:** 2021-02-15

**Authors:** Ditte Demontis, Raymond K. Walters, Veera M. Rajagopal, Irwin D. Waldman, Jakob Grove, Thomas D. Als, Søren Dalsgaard, Marta Ribasés, Jonas Bybjerg-Grauholm, Maria Bækvad-Hansen, Thomas Werge, Merete Nordentoft, Ole Mors, Preben Bo Mortensen, ADHD Working Group of the Psychiatric Genomics Consortium (PGC), Ole A. Andreassen, Maria Jesús Arranz, Tobias Banaschewski, Claiton Bau, Mark Bellgrove, Joseph Biederman, Isabell Brikell, Jan K. Buitelaar, Christie L. Burton, Miguel Casas, Jennifer Crosbie, Alysa E. Doyle, Richard P. Ebstein, Josephine Elia, Corfield C. Elizabeth, Eugenio Grevet, Natalie Grizenko, Alexandra Havdahl, Ziarih Hawi, Johannes Hebebrand, Amaia Hervas, Sarah Hohmann, Jan Haavik, Ridha Joober, Lindsey Kent, Jonna Kuntsi, Kate Langley, Henrik Larsson, Klaus-Peter Lesch, Patrick W. L. Leung, Calwing Liao, Sandra K. Loo, Joanna Martin, Nicholas G. Martin, Sarah E. Medland, Ana Miranda, Nina Roth Mota, Robert D. Oades, Josep Antoni Ramos-Quiroga, Andreas Reif, Marcella Rietschel, Herbert Roeyers, Luis Augusto Rohde, Aribert Rothenberger, Paula Rovira, Cristina Sánchez-Mora, Russell James Schachar, Sarojini Sengupta, Maria Soler Artigas, Hans-Christoph Steinhausen, Anita Thapar, Stephanie H. Witt, Li Yang, Tetyana Zayats, Yanli Zhang-James, Bru Cormand, David M. Hougaard, Benjamin M. Neale, Barbara Franke, Stephen V. Faraone, Anders D. Børglum

**Affiliations:** 1 The Lundbeck Foundation Initiative for Integrative Psychiatric Research, iPSYCH, Aarhus, Denmark; 2 Center for Genomics and Personalized Medicine, Aarhus, Denmark; 3 Department of Biomedicine - Human Genetics, Aarhus University, Aarhus, Denmark; 4 Analytic and Translational Genetics Unit, Department of Medicine, Massachusetts General Hospital and Harvard Medical School, Boston, MA USA; 5 Stanley Center for Psychiatric Research, Broad Institute of MIT and Harvard, Cambridge, MA USA; 6 Department of Psychology, Emory University, Atlanta, GA USA; 7 Bioinformatics Research Centre, Aarhus University, Aarhus, Denmark; 8 National Centre for Register-Based Research, Aarhus University, Aarhus, Denmark; 9 Psychiatric Genetics Unit, Group of Psychiatry, Mental Health and Addiction, Vall d’Hebron Research Institute (VHIR), Universitat Autònoma de Barcelona, Barcelona, Catalonia Spain; 10 Department of Psychiatry, Hospital Universitari Vall d’Hebron, Barcelona, Catalonia Spain; 11 Biomedical Network Research Center on Mental Health (CIBERSAM), Instituto de Salud Carlos III, Madrid, Spain; 12 Departament de Genètica, Microbiologia i Estadística, Facultat de Biologia, Universitat de Barcelona, Barcelona, Catalonia Spain; 13 Center for Neonatal Screening, Department for Congenital Disorders, Statens Serum Institut, Copenhagen, Denmark; 14 GLOBE Institute, Center for GeoGenetics, University of Copenhagen, Copenhagen, Denmark; 15 Institute of Biological Psychiatry, MHC Sct. Hans, Mental Health Services Copenhagen, Roskilde, Denmark; 16 Department of Clinical Medicine, University of Copenhagen, Copenhagen, Denmark; 17 Copenhagen University Hospital, Mental Health Centre Copenhagen Mental Health Services in the Capital Region of Denmark, Hellerup, Denmark; 18 Department of Clinical Medicine, Faculty of Health and Medical Sciences, University of Copenhagen, Copenhagen, Denmark; 19 Psychosis Research Unit, Aarhus University Hospital, Aarhus, Denmark; 20 Centre for Integrated Register-Based Research, Aarhus University, Aarhus, Denmark; 21 Centro de Investigación Biomédica en Red de Enfermedades Raras (CIBERER), Instituto de Salud Carlos III, Madrid, Spain; 22 Institut de Biomedicina de la Universitat de Barcelona (IBUB), Barcelona, Catalonia Spain; 23 Institut de Recerca Sant Joan de Déu (IRSJD), Esplugues de Llobregat, Barcelona, Catalonia Spain; 24 Program in Medical and Population Genetics, Broad Institute of MIT and Harvard, Cambridge, MA USA; 25 Department of Human Genetics, Donders Institute for Brain, Cognition and Behaviour, Radboud University Medical Center, Nijmegen, The Netherlands; 26 Department of Psychiatry, Donders Institute for Brain, Cognition and Behaviour, Radboud University Medical Center, Nijmegen, The Netherlands; 27 Departments of Psychiatry and of Neuroscience and Physiology, SUNY Upstate Medical University, Syracuse, NY USA; 28 NORMENT Centre, Division of Mental Health and Addiction, Oslo University Hospital and University of Oslo, Oslo, Norway; 29 KG Jebsen Centre for Neurodevelopment, Division of Mental Health and Addiction, Oslo University Hospital and University of Oslo, Oslo, Norway; 30 Fundacio Docencia i Recerca Mutua Terrassa, University Hospital Mutua Terrassa, Barcelona, Spain; 31 Department of Child and Adolescent Psychiatry, Central Institute of Mental Health, Medical Faculty Mannheim and University of Heidelberg, Mannheim, Germany; 32 Department of Genetics, Instituto de Biociências, Universidade Federal do Rio Grande do Sul, Porto Alegre, Brazil; 33 Adulthood ADHD Outpatient Program (ProDAH), Clinical Research Center, Hospital de Clínicas de Porto Alegre, Porto Alegre, Brazil; 34 Developmental Psychiatry Program, Experimental Research Center, Hospital de Clínicas de Porto Alegre, Porto Alegre, Brazil; 35 School of Psychological Sciences and Turner Institute for Brain and Mental Health, Monash University, Melbourne, Australia; 36 Department of Psychiatry, Massachusetts General Hospital, Boston, MA USA; 37 Department of Psychiatry, Harvard Medical School, Boston, MA USA; 38 Department of Medical Epidemiology and Biostatistics, Karolinska Instituttet, Stockholm, Sweden; 39 Department of Cognitive Neuroscience, Donders Institute for Brain, Cognition and Behavior, Radboud University Medical Center, Nijmegen, The Netherlands; 40 Karakter Child and Adolescent Psychiatry University Center, Nijmegen, The Netherlands; 41 Psychiatry, Neurosciences and Mental Health, The Hospital for Sick Children, University of Toronto, Toronto, ON Canada; 42 Department of Psychiatry and Forensic Medicine, Universitat Autònoma de Barcelona, Barcelona, Catalonia Spain; 43 Department of Psychiatry, University Hospital Vall d’Hebron, Barcelona, Catalonia Spain; 44 Center for Genomic Medicine and Department of Psychiatry, Massachusetts General Hospital and Harvard Medical School, Boston, MA USA; 45 China Center for Behavior Economics and Finance, Southwestern University of Finance and Economics, (SWUFE), Chengdu, Sichuan China; 46 Department of Pediatrics, Nemours A.I. duPont Hospital for Children, Wilmington, DE USA; 47 Department of Psychiatry, Sidney Kimmel Medical College, Thomas Jefferson University, Philadelphia, PA USA; 48 Department of Mental Disorders, Norwegian Institute of Public Health, Oslo, Norway; 49 Department of Psychiatry, Faculty of Medicine, Universidade Federal do Rio Grande do Sul, Porto Alegre, Brazil; 50 Department of Psychiatry and Douglas Hospital Research Centre, Douglas Mental Health Univerity Institute, McGill University, Montreal, QC Canada; 51 Department of Child and Adolescent Psychiatry, Psychosomatics and Psychotherapy, University Hospital Essen, University of Duisburg-Essen, Essen, Germany; 52 Child and Adolescent Service, University Hospital Mutua Terrassa &Institute of global comprehensive attention to neurodevelopment (IGAIN), Barcelona, Spain; 53 Centre for Neuropsychiatric Disorders, Department of Biomedicine, University of Bergen, Bergen, Norway; 54 Bergen Centre of Brain Plasticity, Division of Psychiatry, Haukeland University Hospital, Bergen, Norway; 55 University of St Andrews, St. Andrews, UK; 56 Social, Genetic and Developmental Psychiatry Centre, Institute of Psychiatry, Psychology and Neuroscience, King’s College London, London, UK; 57 MRC Centre for Neuropsychiatric Genetics & Genomics, School of Medicine, Cardiff University, Cardiff, UK; 58 School of Psychology, Cardiff University, Cardiff, UK; 59 School of Medical Sciences, Örebro University, Örebro, Sweden; 60 Division of Molecular Psychiatry, Center of Mental Health, University of Wuerzburg, Wuerzburg, Germany; 61 Department of Neuroscience, School for Mental Health and Neuroscience (MHENS), Maastricht University, Maastricht, The Netherlands; 62 Laboratory of Psychiatric Neurobiology, Institute of Molecular Medicine, I.M. Sechenov First Moscow State Medical University, Moscow, Russia; 63 Department of Psychology, The Chinese University of Hong Kong, Hong Kong, China; 64 Department of Human Genetics, McGill University, Montreal, QC Canada; 65 Semel Institute for Neuroscience and Human Behavior, UCLA David Geffen School of Medicine, University of California, Los Angeles, CA USA; 66 QIMR Berghofer Medical Research Institute, Brisbane, QLD Australia; 67 Department of Developmental and Educational Psychology, University of Valencia, Valencia, Spain; 68 Department of Human Genetics, Radboud University Medical Center, Nijmegen, The Netherlands; 69 ADHD Outpatient Clinic, Hospital de Clínicas de Porto Alegre, Porto Alegre, Brazil; 70 Clinic for Child and Adolescent Psychiatry and Psychotherapy, University of Duisburg-Essen, Essen, Germany; 71 Department of Psychiatry, Psychosomatic Medicine and Psychotherapy, University Hospital Frankfurt, Frankfurt am Main, Germany; 72 Department of Genetic Epidemiology in Psychiatry, Central Institute of Mental Health, Medical Faculty Mannheim and University of Heidelberg, Mannheim, Germany; 73 Department of Experimental Clinical and Health Psychology, Ghent University, Ghent, Belgium; 74 Department of Psychiatry, Universidade Federal do Rio Grande do Sul, Porto Alegre, Brazil; 75 ADHD Outpatient & Development Psychiatry Program, Hospital de Clínicas de Porto Alegre, Porto Alegre, Brazil; 76 Child and Adolescent Psychiatry, University Medical Center Göttingen, Göttingen, Germany; 77 Department of Child and Adolescent Psychiatry, University of Southern Denmark, Odense, Denmark; 78 Clinical Psychology and Epidemiology, Institute of Psychology, University of Basel, Basel, Switzerland; 79 Department of Child and Adolescent Psychiatry, University of Zurich, Zurich, Switzerland; 80 Child and Adolescent Mental Health Centre, Peking University Sixth Hospital/Institute of Mental Health, Beijing, China; 81 PROMENTA, Oslo University, Oslo, Norway; 82 Department of Psychiatry, SUNY Upstate Medical University, Syracuse, NY USA

**Keywords:** Genome-wide association studies, ADHD, Genome-wide association studies, ADHD

Correction to: *Nature Communications* 10.1038/s41467-020-20443-2, published online 25 January 2021.

The original version of this Article contained an error in the spelling of the author Marta Ribasés, which was incorrectly given as Marta Ribasas. This has now been corrected in both the PDF and HTML versions of the Article.

