# Supplementary material for: Risk variants and polygenic architecture of disruptive behavior disorders in the context of attention-deficit/hyperactivity disorder
Source: Nat Commun. 2021 Jan 25;12:576. doi: 10.1038/s41467-020-20443-2 (PMC7835232; doi:10.1038/s41467-020-20443-2)
Supplement: Supplementary file 1 — Supplementary Information [file 41467_2020_20443_MOESM1_ESM.pdf]

Supplementary Information

Risk variants and polygenic architecture of disruptive behavior disorders in the context of attention-deficit/hyperactivity disorder

Table of Contents

Supplementary Figure 1.A-C. Regional association plots of the genome-wide significant loci 3

Supplementary Figure 2.A-C. Forest plots of genome-wide significant index variants 4

Supplementary Figure 3. Test for heterogeneity across cohorts in the meta-analysis of ADHD+DBDs 5

Supplementary Figure 4. Q-Q plot from test for heterogeneity between European cohorts 6

Supplementary Figure 5. Q-Q plot from GWAS of the Chinese Cohort 7

Supplementary Figure 6. Test for heterogeneity across cohorts in the cross ancestry GWAS 7

Supplementary Figure 7. Q-Q plot from test for heterogeneity between European and Chinese cohorts 8

Supplementary Figure 8.A-C. Regional association in the Chinese cohort 9

Supplementary Figure 9. Manhattan plot of GWAS meta-analysis of European and Chinese cohorts 10

Supplementary Figure 10. Manhattan plot from gene-based MAGMA association analysis 11

Supplementary Figure 11.A-G. Quintile plots of odds ratio for ADHD+DBDs by PRS 12

Members of the Psychiatric Genomics Consortium - ADHD Working Group (including the iPSYCH-Broad Group) not overlapping with contributing consortium members 13

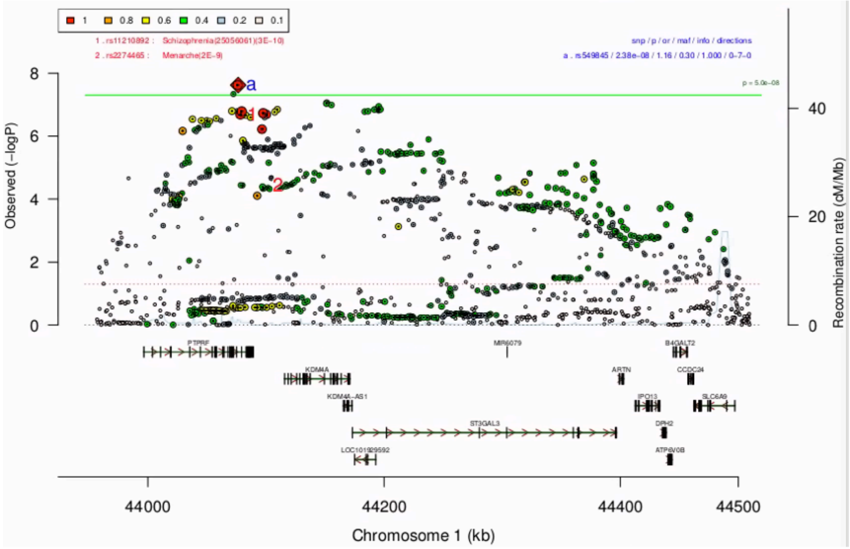
1 0.8 0.6 0.4 0.2 0.1
1 . rs11210892 : Schizophrenia(25056061)(3E-10)
2 . rs2274465 : Menarche(2E-9)
snp / p / or / maf / info / directions
a . rs549845 / 2.38e-08 / 1.16 / 0.30 / 1.000 / 0-7-0
p = 5.0e-08
Observed (−logP)
Recombination rate (cM/Mb)
PTPRF
KDM4A
KDM4A−AS1
ST3GAL3
MIR6079
ARTN
IPO13
DPH2
ATP6V0B
B4GALT2
CCDC24
SLC6A9
44000
44200
44400
44500
Chromosome 1 (kb)

A.

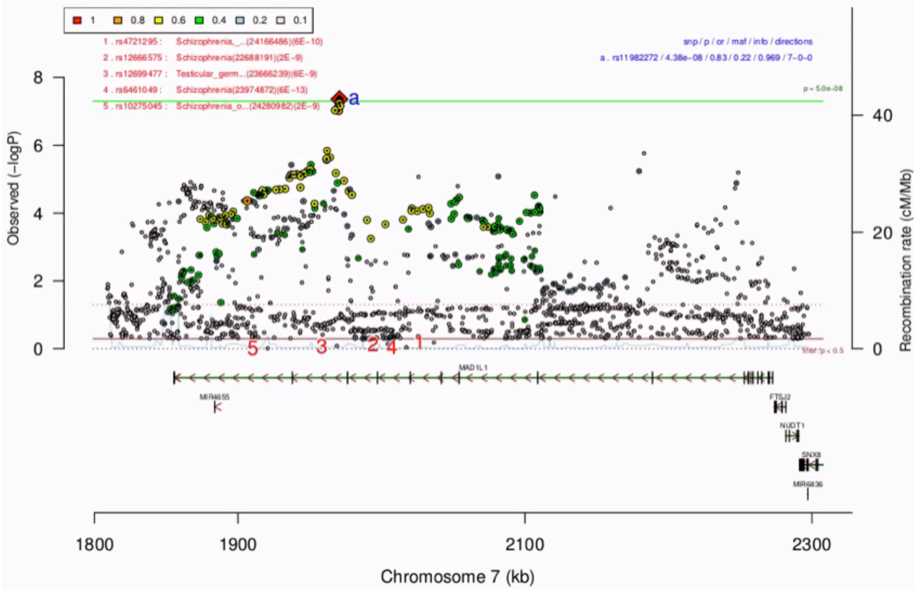
1 0.8 0.6 0.4 0.2 0.1
1 . rs4721295 : Schizophrenia,_...(24166486)(6E−10)
2 . rs12666575 : Schizophrenia(22688191)(2E−9)
3 . rs12699477 : Testicular_germ...(23666239)(6E−9)
4 . rs6461049 : Schizophrenia(23974872)(6E−13)
5 . rs10275045 : Schizophrenia_o...(24280982)(2E−9)
snp / p / or / maf / info / directions
a . rs11982272 / 4.38e−08 / 0.83 / 0.22 / 0.969 / 7−0−0
p = 5.0e-08
Observed (−logP)
Recombination rate (cM/Mb)
MAD1L1
MIR4655
FTSJ2
NUDT1
SNX8
MIR6836
1800
1900
2100
2300
Chromosome 7 (kb)

B.

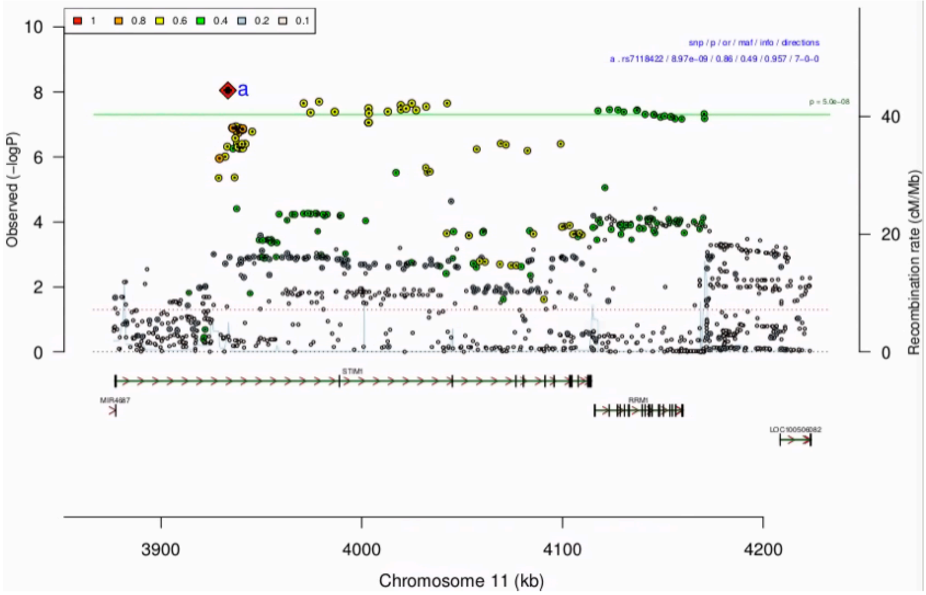

C.

Supplementary Figure 1.A-C. Regional association plots of the genome-wide significant loci

Regional association plots of the local association results from the three genome-wide significant loci identified in the GWAS meta-analysis of ADHD+DBDs (3,802 cases and 31,305 controls). The y-axis represents –log(P-values) of variant association; the P-values are two-sided from meta-analysis using an inverse-variance weighted fixed effects model. The horizontal green line represents the threshold for genome-wide significance (P =$5x10^{-8}$). Location and orientation of the genes in the region is indicated, LD estimates of surrounding SNPs with the index SNP (r^2 values estimated based on 1KGP3) is indicated by colour (colour bar in upper left corner indicates r^2 values). Additionally, the local estimation of recombination rate is indicated in light blue (legend on vertical axis at right). Detailed SNP info in upper right corner (blue letters): SNP name (rsid), P-value (p), odds ratio (or), minor allele frequency(maf), imputation INFO score (info), directions in the analyzed cohorts (risk increasing - decreasing - missing). **A.** Regional association of the variants located the genome-wide significant locus on chromosome 1 **B.** Regional association of the variants located the genome-wide significant locus on chromosome 7 **C.** Regional association of the variants located the genome-wide significant locus on chromosome 11.

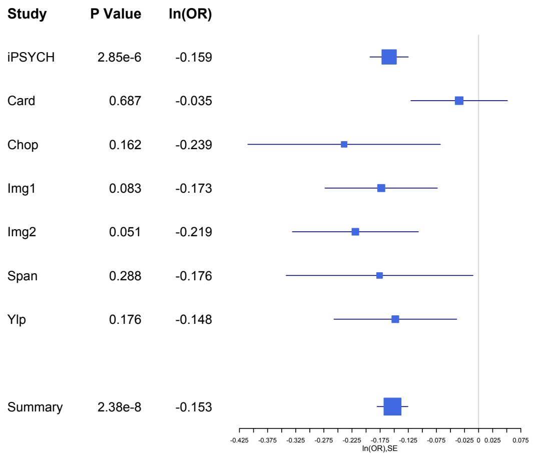

A.

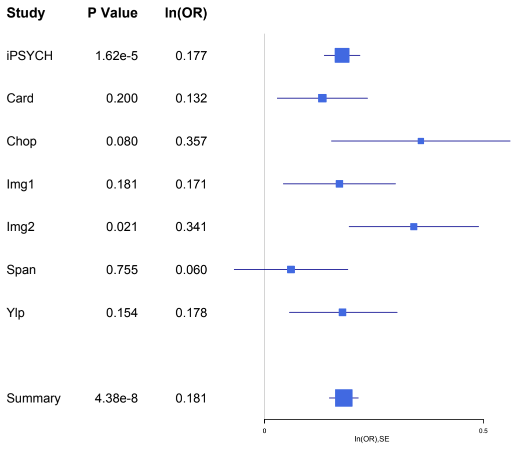

B.

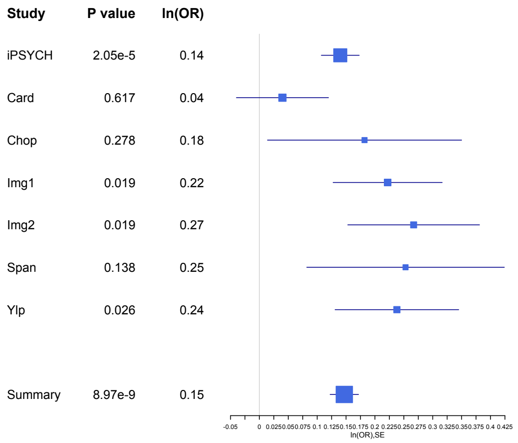

C.

Supplementary Figure 2.A-C. Forest plots of genome-wide significant index variants

Forest plots for the index variants in the three genome-wide significant loci identified in the GWAS meta-analysis of ADHD+DBDs (3,802 cases and 31,305 controls). The plots provides a visualization of the effect size estimates, with the center representing the natural logarithm of the odds ratio (ln(OR) in each included cohort, estimated from logistic regression and for the summary meta-analysis using an inverse-variance weighted fixed effects model. In addition, the standard error intervals for the effect size estimates represented by horizontal lines. **A.** Forest plot for the index variant rs549845 in the genome-wide significant locus on chromosome 1. **B.** Forest plot for the index variant rs11982272 in the genome-wide significant locus on chromosome 7. **C.** Forest plots for the index variant rs7118422 in the genome-wide significant locus on chromosome 11.

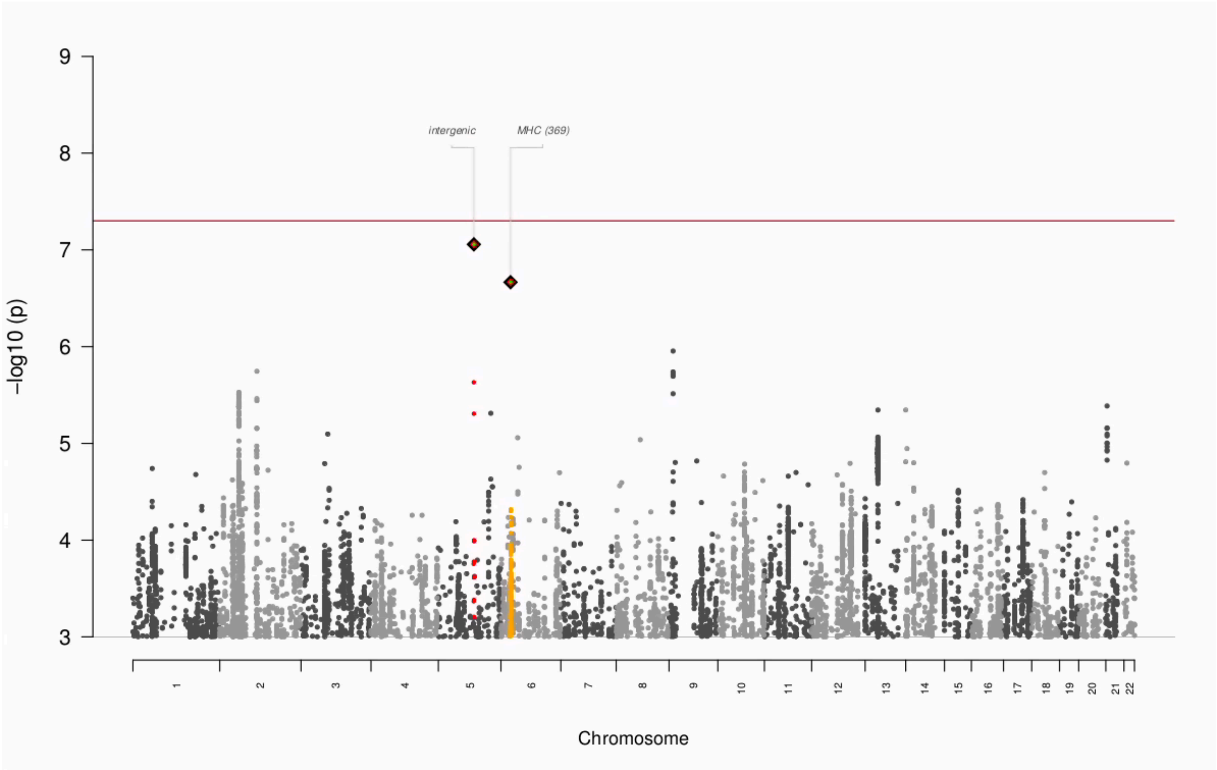

Supplementary Figure 3. Test for heterogeneity across cohorts in the meta-analysis of ADHD+DBDs

The y-axis represents –log(P-values) from omnibus test of heterogeneity across cohorts tested with Cochran's Q test and quantified with the I^2 heterogeneity index. See Supplementary Table 2 for sample sizes. Red horizontal line indicates genome-wide significance threshold ($P = 5x10^{-8}$).

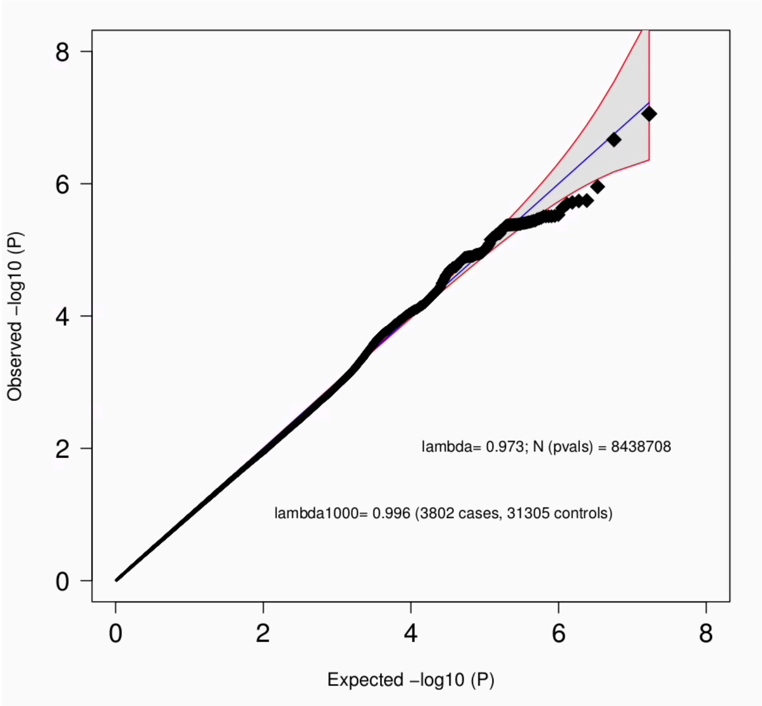

Supplementary Figure 4. Q-Q plot from test for heterogeneity between European cohorts

Quantile-quantile plot of P-values from the omnibus test of heterogeneity (I squared statistic (I^2)) between all cohorts in the GWAS meta-analysis of ADHD+DBDs. See Supplementary Table 2 for sample sizes of cohorts. Each dot represents the observed -log10 P-value, the blue line indicates the distribution under the null hypothesis and the shaded area indicates the 95% confidence interval.

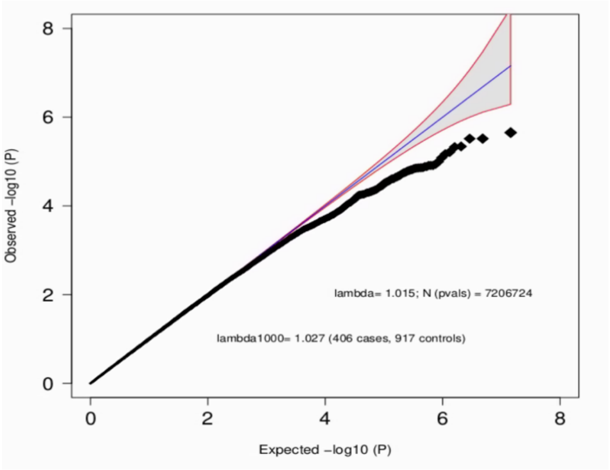

Supplementary Figure 5. Q-Q plot from GWAS of the Chinese Cohort

Quantile-quantile plot of the -log10 P-values from the GWAS of the Chinese cohort. Each dot represents the observed -log10 P-value, the blue line indicates the distribution under the null hypothesis and the shaded area indicates the 95% confidence interval.

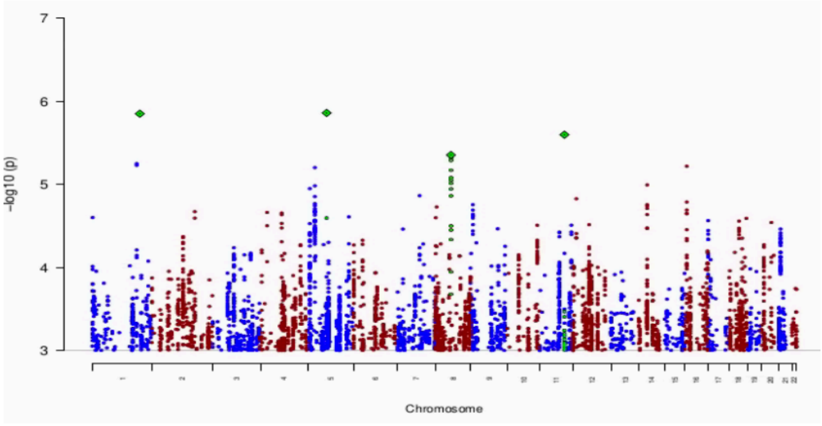

Supplementary Figure 6. Test for heterogeneity across cohorts in the cross ancestry GWAS

Manhattan plot from heterogeneity test of heterogeneity across the European iPSYCH and PGC cohorts and the Chinese cohort. The y-axis represents –log(P-values) from omnibus test of heterogeneity across cohorts tested with Cochran's Q test and quantified with the I^2 heterogeneity index. See Supplementary Table 9 for sample sizes.

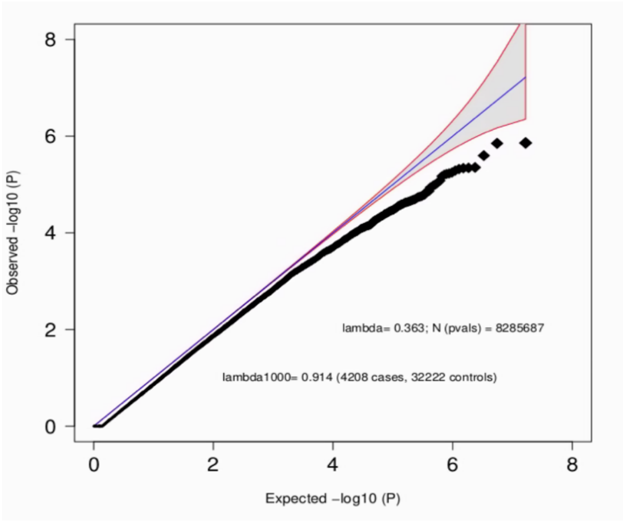

Supplementary Figure 7. Q-Q plot from test for heterogeneity between European and Chinese cohorts

Quantile-quantile plot of P-values from the omnibus test of heterogeneity (I squared statistic (I2)) between cohorts. See Supplementary Table 9 for sample sizes of cohorts. Each dot represents the observed -log10 P-value, the blue line indicates the distribution under the null hypothesis and the shaded area indicates the 95% confidence band.

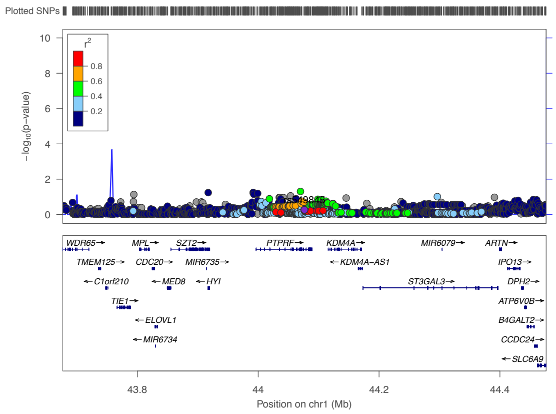

A.

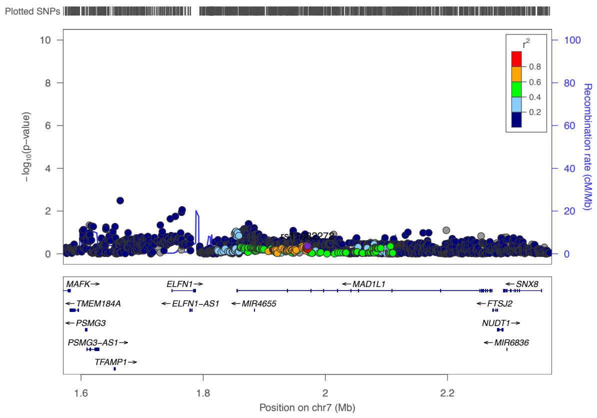

B.

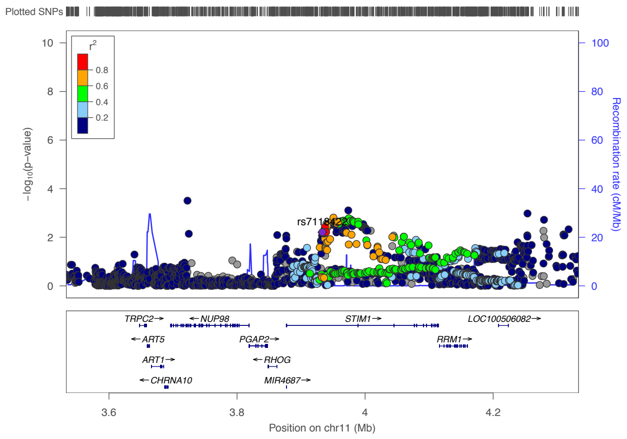

C.

Supplementary Figure 8.A-C. Regional association in the Chinese cohort

Regional association plots of the local association results for the Chinese cohort in the three genome-wide significant loci identified in the GWAS meta-analysis of ADHD+DBDs. The y-axis represents –log(P-values) of variant association; the P-values are two-sided from logistic regression. Location and orientation of the genes in the region is indicated, LD estimates of surrounding SNPs with the index SNP (r^2 values estimated based on 1KGP3) is indicated by color (color bar in upper left corner indicates r^2 values). Additionally, the local estimation of recombination rate is indicated in light blue (legend on vertical axis at right). **A**. Local association surrounding the index variant rs549845 on chromosome 1 **B.** Local association surrounding the index variant rs11982272 on chromosome 7 **C.** Local association surrounding the index variant rs7118422 on chromosome 11.

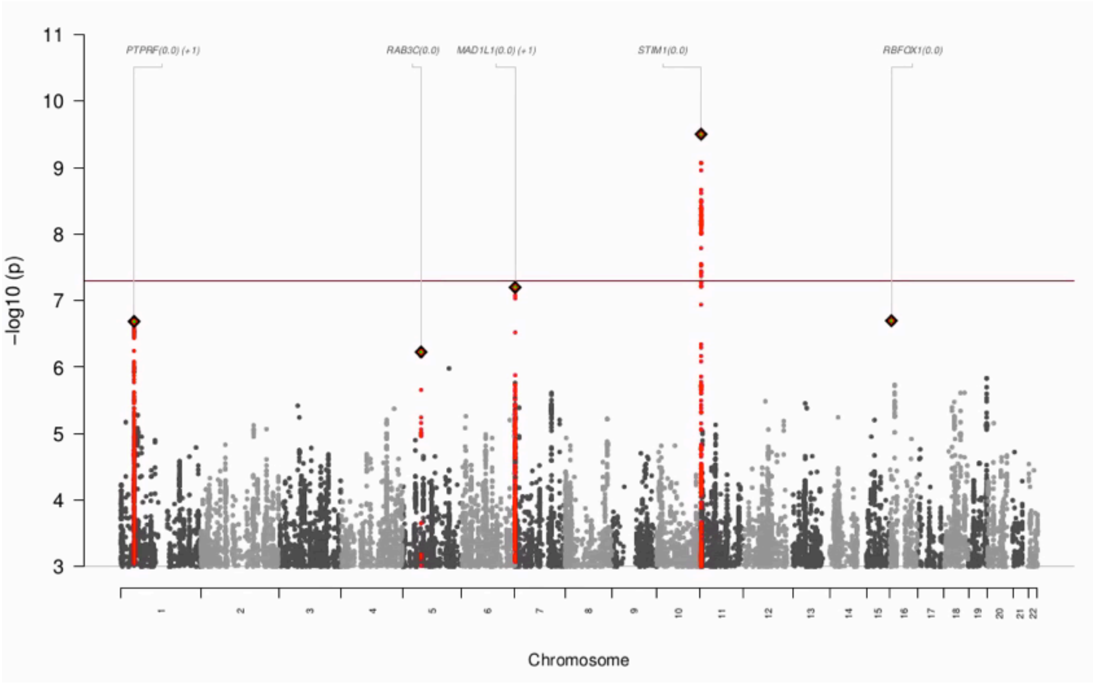

Supplementary Figure 9. Manhattan plot of GWAS meta-analysis of European and Chinese cohorts

Results from cross-ancestry GWAS meta-analysis of iPSYCH and PGC cohorts with European and Chinese ancestries. Two-sided P-values from meta-analysis using an inverse-variance weighted fixed effects model, and a sample size of 4,208 cases and 32,222 controls. The red horizontal line represents the threshold for genome-wide significant association ($P = 5x10^{-8}$).

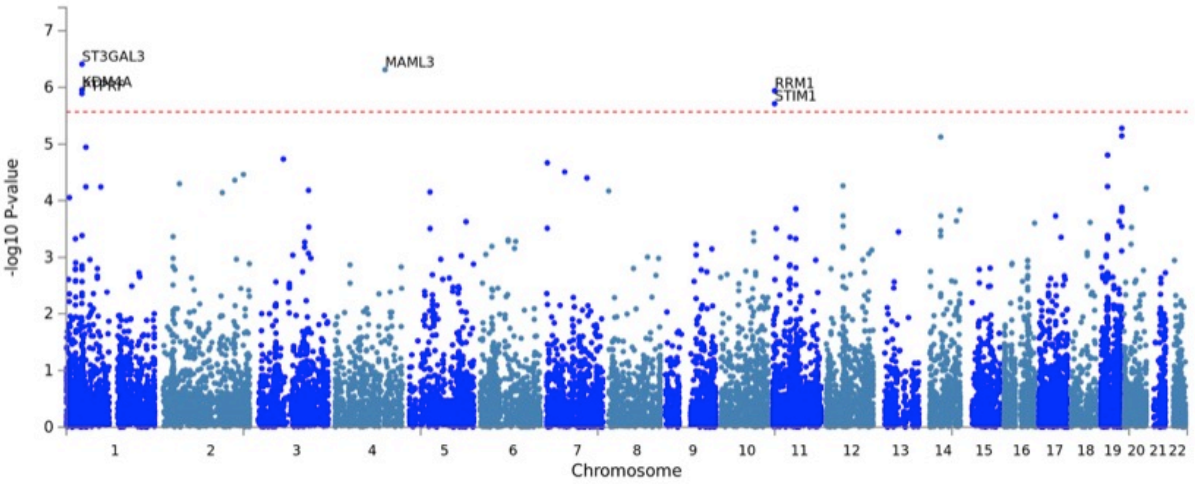

Supplementary Figure 10. Manhattan plot from gene-based MAGMA association analysis

The y-axis represents –log(P-values) of gene-based association with ADHD; P-values are two-sided from MAGMA analysis based on summary statistics from the GWAS meta-analysis of 3,802 cases and 31,305 controls. The horizontal red dotted line represents the threshold for exome-wide significance ($P = 2.7x10^{-6}$).

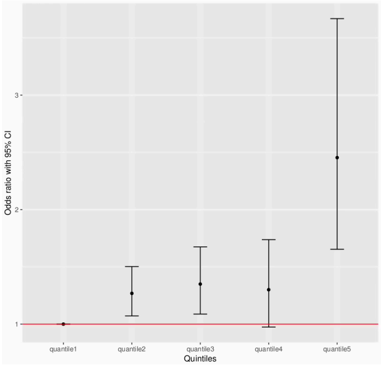

A.

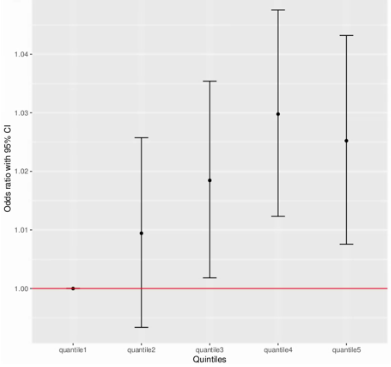

B.

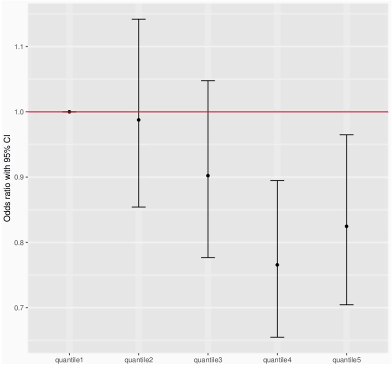

C.

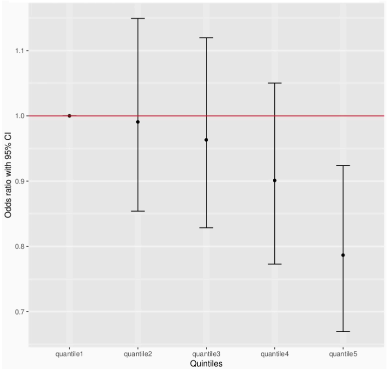

D.

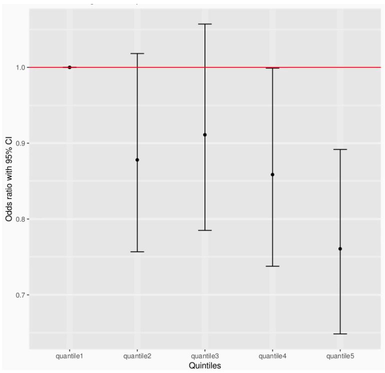

E.

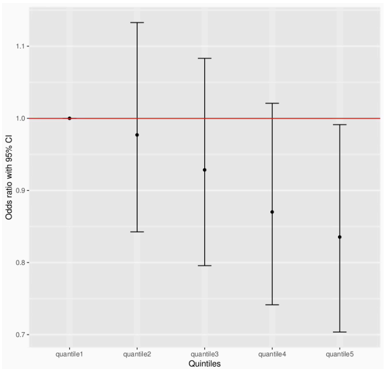

F.

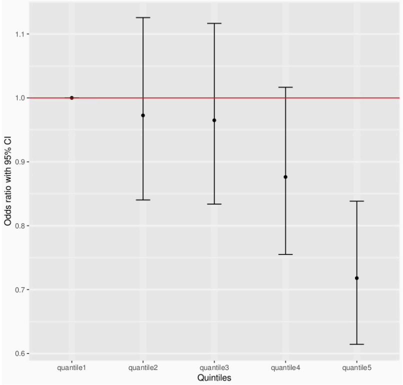

G.

Supplementary Figure 11.A-G. Quintile plots of odds ratio for ADHD+DBDs by PRS

Odds Ratio (OR) by PGS within each quintile for ADHD+DBDs compared to ADHDwoDBDs. The plots represent the seven phenotypes (detailed results on Supplementary Table 8) demonstrating significant association of PGS with ADHD+DBDs after correcting for multiple testing. The center represents the odds ratio and error bars indicate 95% confidence limits **A.** PGS for ADHD **B.** PGS for aggression in children **C.** PGS for number of educational years **D.** PGS for college completion. **E.** PGS for human intelligence **F.** PGS for verbal numerical reasoning. **G.** PGS for age at first birth.

Members of the Psychiatric Genomics Consortium - ADHD Working Group (including the iPSYCH-Broad Group) not overlapping with contributing consortium members

Esben Agerbo[1,2,3], Özgür Albayrak[4,5], Richard J.L. Anney[6,7], Alejandro Arias Vasquez[8,9,10], Philip Asherson[11], Gísli Baldursson[12], Richard Belliveau[13], Dorret I. Boomsma[14,15], Felecia Cerrato[13], Kimberly Chambert[13], Alice Charach[16], Claire Churchhouse[13,17,18], Mark J. Daly[13,17,18,19], Astrid Dempfle[20], Ashley Dumont[13], Nicholas Eriksson[21], Manuel Föcker[22], Marie-Eve Fortier[23], Christine Freitag[22], Michael J. Gandal[24,25,26,27], Joel Gelernter[28,29], Daniel H. Geschwind[24,25,30], Laura Ghirardi[31], Michael Gill[32], Jacqueline I. Goldstein[13,17,18], Katrina L. Grasby[33], Olafur O. Gudmundsson[12,34,35], Hakon Hakonarson[36], Christine S. Hansen[1,37,38], Mads Engel Hauberg[1,39,40], Beate Herpertz-Dahlmann[41], Anke Hinney[4], Mads V. Hollegaard[1,37], Peter Holmans[42], Daniel P. Howrigan[13,17], Hailiang Huang[13,17], Mara Hutz[43], Abel Ickowitz[16], Stefan Johansson[44], Sarah Kittel-Schneider[45], Henry R. Kranzler[46,47], Nanda Lambregts-Rommelse[8,48], S. Hong Lee[49,50,51], Gerd Lehmkuhl[52], Paul Lichtenstein[31], Julian B. Maller[13,53], Alicia R. Martin[13,17,18], Manuel Mattheisen[1,39,40,54,55,56], James J. McGough[57], Jobst Meyer[58], Eric Mick[59], Christel M. Middeldorp[14,60,61], Frank Middleton[62,63], Jennifer L. Moran[13], Fernando Mulas[64], Aisling Mulligan[65], Stanley F. Nelson[30], T. Trang Nguyen[66], Michael C. O'Donovan[42], Michael J. Owen[42], Jonatan Pallesen[1,39,40], Haukur Palmason[67], Duncan S. Palmer[13,17], Carsten Bøcker Pedersen[1,2,3], Marianne Giørtz Pedersen[1,2,3], Timothy Poterba[13,17], Jesper Buchhave Poulsen[1,37], Tobias J. Renner[68,69], Stephan Ripke[13,17,70], Olga Rivero[71], Elise B. Robinson[17,72], Marcel Romanos[73], Jasmin Romanos[73], Panos Roussos[74,75,76,77], Giovanni A Salum[78,79], F. Kyle Satterstrom[13,17,18], Helmut Schäfer[66], André Scherag[80,81], Benno G. Schimmelmann[82], Joseph Sergeant[83], Judith Sinzig[52,84], Pamela Sklar[74,75,76], Susan L. Smalley[85], Edmund J. S. Sonuga-Barke[86], Hreinn Stefansson[34], Kari Stefansson[34,35], Christine Stevens[13], Patrick F. Sullivan[31,87], Margaret Thompson[88], Alexandre Todorov[89], Joyce Tung[21], Patrick Turley[13,17], Susanne Walitza[90], G. Bragi Walters[34,35], Yufeng Wang[91], Andreas Warnke[73], Nigel Williams[42], Hyejung Won[24,25], Naomi R. Wray[49], Margaret J. Wright[49]

Affiliations:

1 The Lundbeck Foundation Initiative for Integrative Psychiatric Research, iPSYCH, Denmark
2 National Centre for Register-Based Research, Aarhus University, Aarhus, Denmark
3 Centre for Integrated Register-Based Research, Aarhus University, Aarhus, Denmark
4 Department of Child and Adolescent Psychiatry, Psychosomatics and Psychotherapy, University Hospital Essen, University of Duisburg-Essen, Essen Germany
5 Department of Psychosomatic Medicine and Psychotherapy, Hannover Medical School (MHH), Hannover, Germany
6 MRC Centre for Neuropsychiatric Genetics & Genomics, School of Medicine, Cardiff University, Cardiff, UK
7 Institute of Psychological Medicine and Clinical Neurosciences, School of Medicine, Cardiff University, Cardiff, UK
8 Department of Psychiatry, Donders Institute for Brain, Cognition and Behaviour, Radboud University Medical Center, Nijmegen, The Netherlands
9 Department of Human Genetics, Donders Institute for Brain, Cognition and Behaviour, Radboud University Medical Center, Nijmegen, The Netherlands
10 Department of Cognitive Neuroscience, Donders Institute for Brain, Cognition and Behavior, Radboud University Medical Center, Nijmegen, The Netherlands
11 Social, Genetic and Developmental Psychiatry Centre, Institute of Psychiatry, Psychology and Neuroscience, King's College London, London, UK

12 Department of Child and Adolescent Psychiatry, National University Hospital, Reykjavik, Iceland
13 Stanley Center for Psychiatric Research, Broad Institute of MIT and Harvard, Cambridge, MA, USA
14 Department of Biological Psychology, Neuroscience Campus Amsterdam, VU University, Amsterdam, The Netherlands
15 EMGO Institute for Health and Care Research, Amsterdam, The Netherlands
16 The Hospital for Sick Children, University of Toronto, Toronto, Ontario, Canada
17 Analytic and Translational Genetics Unit, Department of Medicine, Massachusetts General Hospital and Harvard Medical School, Boston, MA, USA
18 Program in Medical and Population Genetics, Broad Institute of MIT and Harvard, Cambridge, MA, USA
19 Institute for Molecular Medicine Finland (FIMM), Helsinki, Finland
20 Institute for Medical Informatics and Statistics, University Medical Center Schleswig-Holstein, Campus Kiel, Kiel, Germany
21 23andMe, Mountain View, CA, USA
22 Department of Child and Adolescent Psychiatry, Psychosomatics and Psychotherapy,University Hospital Frankfurt, Frankfurt am Main, Germany
23 Douglas Mental Health Univerity Institute, Douglas Mental Health Univerity Institute, Montreal, Quebec, Canada
24 Program in Neurogenetics, Department of Neurology, David Geffen School of Medicine, University of California, Los Angeles, Los Angeles, CA, USA
25 Center for Autism Research and Treatment and Center for Neurobehavioral Genetics, Semel Institute for Neuroscience and Human Behavior, University of California, Los Angeles, Los Angeles, CA, USA
26 Department of Human Genetics, David Geffen School of Medicine; University of California, Los Angeles, Los Angeles, CA, USA
27 Department of Psychiatry, Semel Institute for Neuroscience and Human Behavior, University of California, Los Angeles, Los Angeles, CA, USA
28 Department of Psychiatry, Genetics, and Neuroscience, Yale University School of Medicine, New Haven, CT, USA
29 Veterans Affairs Connecticut Healthcare Center, West Haven, CT, USA
30 Department of Human Genetics, David Geffen School of Medicine, University of California, Los Angeles, CA, USA
31 Department of Medical Epidemiology and Biostatistics, Karolinska Instituttet, Stockholm, Sweden
32 Neuropsychiatric Genetics Research Group, Department of Psychiatry and Trinity College Institute of Neuroscience, Trinity College Dublin, Dublin, Ireland
33 QIMR Berghofer Medical Research Institute, Brisbane, Queensland, Australia
34 deCODE genetics/Amgen, Reykjavík, Iceland
35 Faculty of Medicine, University of Iceland, Reykjavík, Iceland
36 The Center for Applied Genomics, The Children´s Hospital of Philadelphia, Philadelphia, PA, USA
37 Center for Neonatal Screening, Department for Congenital Disorders, Statens Serum Institut, Copenhagen, Denmark
38 Institute of Biological Psychiatry, MHC Sct. Hans, Mental Health Services Copenhagen, Roskilde, Denmark

39 Centre for Integrative Sequencing, iSEQ, Aarhus University, Aarhus, Denmark
40 Department of Biomedicine - Human Genetics, Aarhus University, Aarhus, Denmark
41 Department of Child and Adolescent Psychiatry and Psychosomatic Medicine of University Clinics, RWTH Aachen University Hospital, Aachen, Germany
42 MRC Centre for Neuropsychiatric Genetics & Genomics, Department of Psychological Medicine and Neurology, School of Medicine, Cardiff University, Cardiff, UK
43 Department of Genetics, Instituto de Biociências, Universidade Federal do Rio Grande do Sul, Porto Alegre, Brazil
44 K.G. Jebsen Centre for Neuropsychiatric Disorders, Department of Clinical Science, University of Bergen, Bergen, Norway
45 Department of Psychiatry, Psychosomatic Medicine and Psychotherapy, University Hospital Frankfurt, Frankfurt am Main, Germany
46 Department of Psychiatry, The Perelman School of Medicine, University of Pennsylvania, Philadelphia, PA, USA
47 Veterans Integrated Service Network (VISN4) Mental Illness Research, Education, and Clinical Center (MIRECC), Crescenz VA Medical Center, Philadelphia, PA, USA
48 Karakter Child and Adolescent Psychiatry University Center, Nijmegen, The Netherlands
49 Queensland Brain Institute, University of Queensland, Brisbane, Australia
50 School of Environmental and Rural Science, University of New England, Armidale, New South Wales, Australia
51 Centre for Population Health Research, School of Health Sciences and Sansom Institute of Health Research, University of South Australia, Adelaide, Australia
52 Department of Child and Adolescent Psychiatry, University of Cologne, Cologne, Germany
53 Genomics plc, Oxford, UK
54 Centre for Psychiatry Research, Department of Clinical Neuroscience, Karolinska Instituttet, Stockholm, Sweden
55 Stockholm Health Care Services, Stockholm County Council, Stockholm, Sweden
56 Department of Psychiatry, Psychosomatics and Psychotherapy, University of Wuerzburg, Wuerzburg, Germany
57 Semel Institute for Neuroscience & Human Behavior, David Geffen School of Medicine, University of California, Los Angeles, CA, USA
58 Institute of Psychobiology, Department of Neurobehavioral Genetics, University of Trier, Trier, Germany
59 Quantitative Health Sciences, University of Massachusetts Medical School, Worcester, MA, USA
60 Child Health Research Centre, University of Queensland, Brisbane, Australia
61 Child and Youth Mental Health Service, Children's Health Queensland Hospital and Health Service, Brisbane, Australia
62 Department of Neuroscience and Physiology, SUNY Upstate Medical University, Syracuse, NY, USA
63 Department of Psychiatry, SUNY Upstate Medical University, Syracuse, NY, USA
64 Instituto Valenciano de Neurologia Pediatrica (INVANEP), Valencia, Spain
65 Child and Adolescent Psychiatry, University College Dublin, Ireland
66 University of Marburg, Marburg, Germany
67 Landspitali National University Hospital, Reykjavik, Iceland
68 Division of Molecular Psychiatry, ADHD Clinical Research Unit, Department of Psychiatry, Psychosomatics and Psychotherapy, University of Wuerzburg, Wuerzburg, Germany

69 Department of Child and Adolescent Psychiatry, Universitätsklinikum Tübingen, Tübingen, Germany
70 Department of Psychiatry, Charite Universitatsmedizin Berlin Campus Benjamin Franklin, Berlin, Germany
71 Division of Molecular Psychiatry, Center of Mental Health, University of Wuerzburg, Wuerzburg, Germany
72 Department of Epidemiology, Harvard Chan School of Public Health, Boston, MA, USA
73 Department of Child and Adolescent Psychiatry, Psychosomatics and Psychotherapy, University of Wuerzburg, Wuerzburg, Germany
74 Department of Psychiatry, Icahn School of Medicine at Mount Sinai, New York, NY, USA
75 Institute for Genomics and Multiscale Biology, Department of Genetics and Genomic Sciences, Icahn School of Medicine at Mount Sinai, New York, NY, USA
76 Friedman Brain Institute, Department of Neuroscience, Icahn School of Medicine at Mount Sinai, New York, NY, USA
77 Mental Illness Research Education and Clinical Center (MIRECC), James J. Peters VA Medical Center, Bronx, NY, USA
78 Department of Psychiatry, Universidade Federal do Rio Grande do Sul, Porto Alegre, Brazil
79 Section on Negative Affect and Social Processess, Hospital de Clínicas de Porto Alegre, Porto Alegre, Brazil
80 Institute for Medical Informatics, Biometry and Epidemiology (IMIBE), University of Duisburg-Essen, Essen, Germany
81 Clinical Epidemiology, Integrated Research and Treatment Center, Center for Sepsis Control and Care (CSCC), Jena University Hospital, Jena, Germany
82 University Hospital of Child- and Adolescent Psychiatry, University of Bern, Bern, Switzerland
83 Vrije Universiteit, Amsterdam, The Netherlands
84 Department of Child and Adolescent Psychiatry and Psychotherapy, LVR – Clinic Bonn, Bonn, Germany
85 University of California, Los Angeles, CA, USA
86 Institute of Psychiatry, Psychology & Neuroscience, King's College London, London, UK
87 Departments of Genetics and Psychiatry, University of North Carolina, Chapel Hill, NC, USA
88 University of Southampton, Southampton, UK
89 Department of Psychiatry, Washington University School of Medicine, St. Louis, MO, USA
90 Department of Child and Adolescent Psychiatry, University of Zurich, Zurich, Switzerland
91 Institute of Mental Health, Peking University Sixth Hospital, Beijing, China
