# Supplementary material for: Risk variants and polygenic architecture of disruptive behavior disorders in the context of attention-deficit/hyperactivity disorder
Source: Nat Commun. 2021 Jan 25;12:576. doi: 10.1038/s41467-020-20443-2 (PMC7835232; doi:10.1038/s41467-020-20443-2)
Supplement: Supplementary file 3 — Reporting Summary [file 41467_2020_20443_MOESM3_ESM.pdf]

nature research

Corresponding author(s): Ditte Demontis

Last updated by author(s): 09/11/2020

Reporting Summary

Nature Research wishes to improve the reproducibility of the work that we publish. This form provides structure for consistency and transparency in reporting. For further information on Nature Research policies, see our Editorial Policies and the Editorial Policy Checklist.

Statistics

For all statistical analyses, confirm that the following items are present in the figure legend, table legend, main text, or Methods section.

n/a	Confirmed	
☐	☒	The exact sample size (*n*) for each experimental group/condition, given as a discrete number and unit of measurement
☐	☒	A statement on whether measurements were taken from distinct samples or whether the same sample was measured repeatedly
☐	☒	The statistical test(s) used AND whether they are one- or two-sided<br>*Only common tests should be described solely by name; describe more complex techniques in the Methods section.*
☐	☒	A description of all covariates tested
☐	☒	A description of any assumptions or corrections, such as tests of normality and adjustment for multiple comparisons
☐	☒	A full description of the statistical parameters including central tendency (e.g. means) or other basic estimates (e.g. regression coefficient) AND variation (e.g. standard deviation) or associated estimates of uncertainty (e.g. confidence intervals)
☐	☒	For null hypothesis testing, the test statistic (e.g. *F*, *t*, *r*) with confidence intervals, effect sizes, degrees of freedom and *P* value noted<br>*Give P values as exact values whenever suitable.*
☒	☐	For Bayesian analysis, information on the choice of priors and Markov chain Monte Carlo settings
☒	☐	For hierarchical and complex designs, identification of the appropriate level for tests and full reporting of outcomes
☐	☒	Estimates of effect sizes (e.g. Cohen's *d*, Pearson's *r*), indicating how they were calculated

Our web collection on statistics for biologists contains articles on many of the points above.

Software and code

Policy information about availability of computer code

Data collection	For the iPSYCH samples genotyping was performed at the Broad Institute of Harvard and MIT (Cambridge, MA, USA) using Illumina's Beadarrays (PsychChip; Illumina, CA, San Diego, USA) according to the manufacturer's protocols. Genotypes were a result of merging callsets from the calling algorithms GenCall (1.6.2.2) and Birdseed (1.6)
Data analysis	Imputation of genotypes was done using SHAPEIT v2 and IMPUTE v2 (known as IMPUTE2)<br>Quality control and association analyses were done using the Ricopili pipeline: https://github.com/Nealelab/ricopili, which include the follwoing software: Plink 1.9, Eigensoft 6.1.3, METAL 2011-03-25.<br>For gene-based and gene-set analyses we used MAGMA 1.06<br>SNP heritabilities were estimated using LD score regression (https://github.com/bulik/ldsc) and GCTA (gcta_1.91.7)<br>Genetic correlations were calculated using LD score regression (https://github.com/bulik/ldsc)<br>mtCOJO analysis was conducted using mtCOJO implemented in GCTA (gcta_1.91.7)<br>Analyses of the genetically regulated gene expression were done using MetaXcan implemented in the R-package metaxcanr (https://github.com/drveera/) and GTEx v6. perdiction models downloaded from http://predictdb.org<br>Polygenetic score analyses were done using Plink 1.9 and the R package 'BaylorEdPsych'

For manuscripts utilizing custom algorithms or software that are central to the research but not yet described in published literature, software must be made available to editors and reviewers. We strongly encourage code deposition in a community repository (e.g. GitHub). See the Nature Research guidelines for submitting code & software for further information.

Data

Policy information about availability of data

All manuscripts must include a data availability statement. This statement should provide the following information, where applicable:

- Accession codes, unique identifiers, or web links for publicly available datasets
- A list of figures that have associated raw data
- A description of any restrictions on data availability

Summary statistics with the results from the GWAS meta-analysis of ADHD+DBDs are available on the iPSYCH website (https://ipsych.dk/en/research/downloads/).

Field-specific reporting

Please select the one below that is the best fit for your research. If you are not sure, read the appropriate sections before making your selection.

☒ Life sciences ☐ Behavioural & social sciences ☐ Ecological, evolutionary & environmental sciences

For a reference copy of the document with all sections, see nature.com/documents/nr-reporting-summary-flat.pdf

Life sciences study design

All studies must disclose on these points even when the disclosure is negative.

Sample size	No sample size calculation was made. Previous studies of polygenic psychiatric disorders (e.g. schizophrenia) have demonstrated that high numbers of cases and controls are needed in order to detect genome-wide significant loci. In order to maximise power, we based our analyses on the maximal possible sample size from the iPSYCH and the Psychiatric Genetics Consortium (3,802 ADHD+DBDs cases and 31,305 controls), which should yield enough power to detect a few loci/common variants with low effect sizes.
Data exclusions	In GWASs it is important to analyze a sample of unrelated individuals and a genetic homogeneous sample, to avoid false positives caused by relatedness and population stratification. Related individuals were excluded as well as genetic outliers (identified based on identity by state analysis and principal component analysis)
Replication	In order to maximize power all cohorts were included all available European cohorts in the primary GWAS meta-analysis ADHD+DBDs. Consistency in direction of the effect sizes were evaluated over the included cohorts illustrated in the forest plots (figure 2). Replication/trans-ancestry association was evaluated in a GWAS meta-analysis of the Euroepan cohorts and a Chinese cohort.
Randomization	Allocation into groups was not random. Individuals were allocated into the case group based on having a diagnosis of ADHD+DBS. The controls in each cohort did not have a diagnosis of ADHD+DBDs.
Blinding	In iPSYCH, diagnoses are drawn from registries. These are administrative data-bases populated by data from the clinicians long before the current study. The blood samples are pulled from a biobank. Hence, the study participants and diagnosing clinicians are blinded with respect to this study. Genotyping is done on a massive scale on 85.000 individuals on 500.000 variables (which by imputation is expanded to ~10 million variables), and the data is generated without a specif goal or effect in mind except for an overall goal of investigating the genetic and environmental effects on psychiatric disorders. So although it is in principle possible for analysts in the lab to look up crude diagnostic data for a sample, it will not change the genotyping. - In the meta analysis we include data from the Psychiatric Genetics Consortium (PGC). The design was different, but analyses analogous.<br>No blinding was done in this study with respect to the GWAS as the analyst needs to know who is case and who is control in order to do the analysis.

Reporting for specific materials, systems and methods

We require information from authors about some types of materials, experimental systems and methods used in many studies. Here, indicate whether each material, system or method listed is relevant to your study. If you are not sure if a list item applies to your research, read the appropriate section before selecting a response.

Materials & experimental systems

n/a	Involved in the study	
☒	☐	Antibodies
☒	☐	Eukaryotic cell lines
☒	☐	Palaeontology and archaeology
☒	☐	Animals and other organisms
☐	☒	Human research participants
☒	☐	Clinical data
☒	☐	Dual use research of concern

Methods

n/a	Involved in the study	
☒	☐	ChIP-seq
☒	☐	Flow cytometry
☒	☐	MRI-based neuroimaging

Human research participants

Policy information about studies involving human research participants

Population characteristics	In the meta-analysis we included 7 cohorts form the Psychiatric Genetics Consortium (PGC) and the iPSYCH consortium. Additionally a Chinese cohort was included in a trans-ancestry GWAS meta-analysis. In the seperate GWASs we corrected for population stratification using relevant principal components from principal component analyses as covariates to correct for population stratification.<br>The iPSYCH sample was processed in 23 batches (genotyping, qc and imputing were done separately for theses batches) of approximately 3,500 individuals each. In order to control for potential batch effects we included "wave" as a covariate in the regression models of all downstream analyses when relevant.<br>Sex was not used as covariate, as we found no indication of sex being a confounder in our analyses (results not shown).
Recruitment	The largest cohort included in the study has been established by the iPSYCH consortium. iPSYCH samples were selected from a birth cohort comprising individuals born in Denmark between May 1, 1981, and December 31, 2005, who were residents in Denmark on their first birthday and who have a known mother (N =1,472,762). The iPSYCH cases were identified based on information in the Danish Psychiatric Central Research Register, and 30,000 randomly selected controls were identified from the same nationwide birth cohort in the Danish Civil Registration System. Subseqeuntly blood spot samples (Guthrie cards) were identified in the in the Danish Newborn Screening Biobank (DNSB) from the included individuals. All genetic analyses were performed at secured servers in Denmark at the GenomeDK high performance-computing cluster (http://genome.au.dk).
Ethics oversight	The study was approved by the Danish Data Protection Agency and the Scientific Ethics Committee in Denmark. Please see Supplementary Table 9, for list of approval agencies for the samples from the Psychiatric Genetics Consortium

Note that full information on the approval of the study protocol must also be provided in the manuscript.
