# Supplementary material for: Risk variants and polygenic architecture of disruptive behavior disorders in the context of attention-deficit/hyperactivity disorder
Source: Nat Commun. 2021 Jan 25;12:576. doi: 10.1038/s41467-020-20443-2 (PMC7835232; doi:10.1038/s41467-020-20443-2)
Supplement: Supplementary file 4 — Description of Additional Supplementary Files [file 41467_2020_20443_MOESM4_ESM.pdf]

Description of Additional Supplementary Files

Supplementary Data 1. Results for the index variant in the genome-wide significant locus on chromosome 11

Supplementary Data 2. Results for the three genome-wide significant loci

Supplementary Data 3. mtCOJO results

Supplementary Data 4. Top associated genes

Supplementary Data 5. Results from analysis of the genetically regulated gene expression

Supplementary Data 6. (A) SNP heritability (B) Test for difference in SNP heritability

Supplementary Data 7. Genetic correlations with aggression related phenotypes

Supplementary Data 8. Polygenic score results

Supplementary Data 9. Distribution of DBDs over ICD-10 codes

Supplementary Data 10. Cohorts included
