# Supplementary material for: Risk variants and polygenic architecture of disruptive behavior disorders in the context of attention-deficit/hyperactivity disorder
Source: Nat Commun. 2021 Jan 25;12:576. doi: 10.1038/s41467-020-20443-2 (PMC7835232; doi:10.1038/s41467-020-20443-2)
Supplement: Supplementary file 2 — Peer Review [file 41467_2020_20443_MOESM2_ESM.pdf]

Reviewers' comments:

Reviewer #1 (Remarks to the Author):

The authors report the largest GWAS meta-analysis of individuals with dual diagnoses of ADHD and disruptive behaviour disorder (DBD). They identify three genome-wide significant loci in this study of European individuals and attempt to dissect their importance relative to ADHD and DBD using a number of sub-analyses including [GWA] meta-analysis with a small (Han?) Chinese cohort. They claim that the results, principally from PRS analyses, show that ADHD+DBD "represents a more severe phenotype with respect to the genetic risk load than ADHD without DBDs".
The study is well described, although the English could be improved throughout, and the analyses steps are clear. However, it is not clear that the analyses that have been performed are the most appropriate to answer the stated questions, which makes it difficult to assess the validity of the claims. In particular the use of secondary GWAS and mtCOJO to dissect the relative importance of the top associated loci to ADHD and DBDs. The key question that this seems to attempt to answer is whether there is a significant difference in the effect of these loci in individuals with ADHD + DBDs versus individuals with ADHD alone. This may be better answered by analysing the full sample and fitting subtype (DBDs v no DBDs) as an interaction term. This may not be possible genome-wide using the analysis package used to analyse the GWAS (which is not stated anywhere in the text or supplementary, although may be within the individuals cohort references), but could clearly be analysed for the top loci if that is a primary interest. Consider using Plink or GCTA's --gxe flags.

Further although a GWAS of the Chinese sample was performed, no details of the QC or results are presented (i.e. genotype QC particularly with respect to MAF, as well as lambda, QQ-plot, rg with European cohorts, whether any genome-wide significant loci were observed) beyond the results of the three loci significant in the European-only meta-analysis. Although, given the small size of this cohort, any significant hits are unlikely, these data would provide confidence in the Chinese sample and improve interpretation of the meta-analysed results. Similarly LocusZoom plots, or similar, of the regions spanning the three GWAS significant loci in the meta-analysed European and separately the Chinese cohort would be informative, as would the MAF of the loci in the Chinese sample. The authors should consider applying the trans-ethnicity fine-mapping approach published by Lam et al, PMID: 31740837 to refine there top three loci.

The phrase "represent a more severe phenotype with respect to the genetic risk load" needs to be revised. The severity of any disease phenotype is independent of the underlying genetic load. Although the authors have shown a higher loading of common risk alleles associated with ADHD in the ADHD+DBDs sample, they show no evidence of a more severe phenotypes in terms of symptoms.

Similarly the evidence that the cohort of ADHD+DBDs show an enrichment for "loci which are distinct to ADHD+DBDs" is unclear. Could the authors clarify the evidence to which they refer.

Can the authors comment on whether results best fit a model where ADHD+DBDs is a subtype of ADHD or individuals co-morbid for two genetic disorders that overlap in their genetic risk factors.

Further comments (in manuscript order)
1) Throughout the English and grammar needs to be checked closely, including the supplementary information. Examples of poor sentence construction/errors include:
i) Page 3 paragraph 1: correct "of of",
replace "like" with "such as",
replace "around" with "approximately",
replace "higher mortality rates than in individuals with ADHD" with "higher mortality rates than in ADHD",
Replace "Among those with DBDs in the same Danish cohort, more than half (57%) also had a comorbid diagnosis of ADHD" with "Among those with DBDs in that same Danish cohort, more than

half (57%) had a comorbid diagnosis of ADHD."
replace "only diagnosed with ADAD" with "diagnosed with ADHD only".

ii) Page 3 paragraph 2: replace ADHD as well as DBDs" with "ADHD and DBDs".

iii) Page 4 Paragraph 3 and elsewhere: consider replacing the term "generalization", its meaning is unclear.

iv) replace "conductict" with "conduct".

v) Page 9 paragraph 2: replace "compared to the GWAS of ADHD without DBDs" with "compared to ADHD without DBDs".

vi) Page 10 paragraph 2 is missing a P= for the results from educational attainment.

vii) Page 11 paragraph 2: replace ".;" with "."

viii) Page 13 paragraph 2: replace "to mainly to" with "to mainly"

ix) Page 13 paragraph 3: replace "Supplementary Table 5" with "Supplementary Table 6"

x) Page 14 Paragraph 1: replace "Going beyond ADHD risk burden, the common variant component of DBDs could also include variants distinctly associated with aggression. This idea is supported by our finding of increased PRS for aggression in ADHD+DBDs compared to ADHD without DBDs..." with "Going beyond ADHD risk burden, the common variant component of DBDs could also include distinct variants associated with aggression only. This idea is supported by our finding of increased PRS for aggression in children in ADHD+DBDs compared to ADHD without DBDs..." However, no evidence is provided that this increase in load is not due to variants with pleiotropic effects on ADHD and aggression in children.

xi) Page 15 paragraph 1: replace "idea is supported by" with "idea is consistent with".

xii) Page 19 paragraph 2 : replace "correct out" with "adjusted for".

xiii) Supplementary figures 1, 3, 5 and 6 replace "vertical" line with "horizontal" line, when referring to the genome-wide significant threshold. And the opposite for Supplementary Figure 2A i.e. replace "vertical lines" with "horizontal bars" represent SE.

xiv) Elsewhere in the supplementary information requiring correction:
"Two-sided P-values represents tests for the difference of the genetic correlations from zero."
"Distribution DBDs over ICD-10 codes among cases with ADHD+DBDs in the iPSYCH cohort"
"..and thier association P-values (P) odds ratio (OR) and standard error (SE).."
"the 95% confidence band."

NB. This is not an exhaustive list, there may be other errors.

2) Page 7 paragraph 3: Refers to Supplementary Table 9 which states rs549845*: *This locus is genome-wide significantly associated with ADHD without DBDs, however with another index variant (rs113551349, P=3.74x10-10, OR=0.89). The table shows that the p-value for ADHD without DBDs is 8x10-8
i.e. NOT genome-wide significant. This also raises the question as to how the loci were selected for this analysis. How was a significant difference in the effect of these loci in the different groups assessed i.e. what statistical test was performed.

3) The mtCOJO analyses do not confirm or refute a significant difference in effect in ADHD versus DBDs.

4) Please give a reference for the jackknife method mentioned on Page 10 paragraph 1.

5) Consider revising the sentence "Our results suggest that the aggressive and disruptive behavioral component of the ADHD+DBDs phenotype has a biological cause..". Surely that is a fact rather than a conclusion from their data.

6) Page 17 Quality control and imputation. Please give the post imputation QC filters applied to the datasets used for the GWASs i.e Imputation quality, MAF, call rates etc. Was the Chinese sample imputed, if so, what reference panel was used.

7) Page 18 paragraph 2: include the covariates used in Supplementary Table 2.

8) PRS analyses, R2 based on the liability scale should be calculated for the binary traits i.e. psychiatric disorders.

9) Consider unifying how "ADHD without DBDs" is named. There is "ADHD without DBDs", "ADHD-only", "ADHDwoDBDs" within the manuscript.

Reviewer #2 (Remarks to the Author):

This report summarizes findings from a genome-wide association study of ADHD+disruptive behavior disorders. The analyses follow a conventional pipeline in the field of psychiatric genetics, and so although the methods are not particularly novel in themselves do provide novel information regarding the genetic architecture of ADHD+DBS.

My only concern with the manuscript is that the selection of the Chinese replication cohort needs additional justification, as it seems the authors would find better-matched cohort to the Danish discovery sample. I would find the replication effort to be more convincing with a more closely matched validation sample.

Reviewer #3 (Remarks to the Author):

This paper reports a GWA of ADHD+DBD (‘A+D’ below) for 3802 cases from 7 cohorts, which combines samples from the Danish iPsych study and the PGC. The authors claim that this is the largest GWA study for A+D, although it is much smaller than the largest GWA study of ADHD.

Three GWA-significant associations, one of which on Chromosome 11 emerged both for European and Chinese samples, but not for the other two associations. Moreover, the C-11 association was not found

in a secondary GWA analysis of 'pure' ADHD cases*. The authors conclude that this C-11 association is specific to the comorbid phenotype. However, this locus has not previously been implicated in ADHD, DBD, aggression or other psychiatric disorders.

The authors report that SNP heritability is greater for A+D than for ADHD without DBD ('A-D' below) in the iPSYCH cohort, although SNP heritability for A+D was lower in the whole sample. ** I was confused by this sentence on page 9: "Additionally, common variants explained a small fraction of the variation in the ADHD+DBDs phenotype compared to the ADHD without DBDs phenotype (h2SNP=0.08; SE=0.04, GCTA estimate) (Supplementary Table 6)."They also report high genetic correlations between A+D and aggressive behaviors (rg = .81) and antisocial behaviors (rg = .82), although A-D was also correlated genetically with aggression (rg = .74).***

The authors report polygenic score analyses indicating that polygenic scores for ADHD and for aggression correlated more highly with A+D than with A-D.****

On the basis of these findings, the authors conclude that A+D has a more severe genetic risk load than A-D and suggest that this might in part be due to A+D links with aggressive behavior. I wasn't convinced by these data that there is much merit in the A+D concept, but it is noteworthy that these suggestive results correspond with twin studies that suggest that A+D is more heritable than A-D and that A and D have only moderate genetic overlap. Also, polygenic score analyses yield higher correlations with A+D than with A alone.

Although the N of 3802 cases is the largest GWA study of A+D, isn't it the case that other disorders didn't reach genome-wide significant results until the N was much larger?

Some of my comments follow from these findings, as asterisked above, most of which have to do with avoiding false positive results. (I assume that none of these results were preregistered hypotheses.)

*I wasn't clear that this was a significant difference. (Finding that the association was significant in one analysis but not in the other does not mean that this is a significant difference.)

**Is this a significant difference in SNP heritability? The standard errors suggest that it is.

***More fundamentally, what is the genetic correlation between A+D and A-D?

****Was this a significant difference?
Most important for me is to know how predictive any of these polygenic scores are with ADHD-related behaviors in independent samples? If I understand the results on page 10, it appears that the effect sizes (OR, R2) are weak. How do these effect sizes compare to those from previous ADHD-related GWA studies. Do any of these polygenic scores (e.g., A, A+D, A-D) improve predictive power? I wasn't clear about the statement on page 10: "Only a small proportion of the variance in ADHD+DBDs (compared to ADHD without DBDs) was explained by the PRSs, with the maximum Nagelkerke's R2 = 0.36% for the ADHD PRS." Does this mean that less than 1% of the variance was explained by the A+D polygenic scores? If not, could the authors include some clear statements as to the variance explained by the A+D polygenic score (as compared to other polygenic scores) in predicting ADHD-related behaviors in independent samples? If, as it seems to me, the present GWA analyses yield polygenic scores that predict less variance than previous studies, can the authors explain this?
A general issue is that other GWA studies of ADHD-related behaviors (including aggression) are about to be published. However, I believe that these involve dimensional traits rather than dichotomous diagnoses. Are the authors aware of impending GWA publications of ADHD-related behaviors? If so, it would be good to future-proof this paper a bit so that it is not superseded by the time it is published.

The authors don't mention 'p', the current notion of genetic overlap among many disorders. Given that they are arguing for a genetic correlation between ADHD symptoms and disruptive symptoms, the

authors might wish to say something about ‘p’.

The paper is well organised and clearly and succinctly written, despite the complexity of the analyses.

Editor's comments:

Please be aware that for certain types of new data, including most types of genetic data, journal policy is that deposition in a community-endorsed, public repository is generally mandatory prior to publication. Data submission can be a lengthy process, and we strongly suggest that you begin this well in advance of potential publication to avoid delays later on. Please include a statement about data availability (specifically GWAS summary statistics) in your point-by-point letter accompanying your revisions.

We will make the results from the GWAS-meta analysis of ADHD+DBDs available for download from the iPSYCH download web-page (https://ipsych.dk/en/research/downloads/). We have added information about data availability at the end of the manuscript.

Please see decision letter above for more detailed information about data requirements and policy. If you are unable to make your data publically available for exceptional reasons, please get in touch with me now to discuss this further.

Reviewers' comments:

Reviewer #1 (Remarks to the Author):

The authors report the largest GWAS meta-analysis of individuals with dual diagnoses of ADHD and disruptive behaviour disorder (DBD). They identify three genome-wide significant loci in this study of European individuals and attempt to dissect their importance relative to ADHD and DBD using a number of sub-analyses including [GWA] meta-analysis with a small (Han?) Chinese cohort. They claim that the results, principally from PRS analyses, show that ADHD+DBD "represents a more severe phenotype with respect to the genetic risk load than ADHD without DBDs".
The study is well described, although the English could be improved throughout, and the analyses steps are clear. However, it is not clear that the analyses that have been performed are the most appropriate to answer the stated questions, which makes it difficult to assess the validity of the claims. In particular the use of secondary GWAS and mtCOJO to dissect the relative importance of the top associated loci to ADHD and DBDs. The key question that this seems to attempt to answer is whether there is a significant difference in the effect of these loci in individuals with ADHD + DBDs versus individuals with ADHD alone. This may be better answered by analysing the full sample and fitting subtype (DBDs v no DBDs) as an interaction term. This may not be possible genome-wide using the analysis package used to analyse the GWAS (which is not stated anywhere in the text or supplementary, although may be within the individual's cohort references), but could clearly be analysed for the top loci if that is a primary interest. Consider using Plink or GCTA's --gxe flags.

We appreciate the input from the reviewer regarding the use of the GxE option to identify loci with different effect sizes in ADHD+DBDs versus ADHD without DBDs. However, this option is unfortunately not possible in the data we have available. When running the GxE option in GCTA the method will constrain the main effect of the first level (ADHD/controls) to be zero and the main effect of the other level (DBDs or not) represents its difference in effect compared to the first level. However, since we only have DBDs in individuals that also have

ADHD (no individuals have only DBDs in the analysis) it is not possible for us to fit our data to this model.
In this study we have instead evaluated the findings from the primary GWAS meta-analysis of ADHD+DBDs against results from two secondary GWAS ("ADHD+DBDs vs ADHD-only" and "ADHD without DBDs"; results listed in Supplementary Table 1). Especially for the genome-wide significant locus on chromosome 11 locus, we find strong support for a specific association ADHD+DBDs and not with ADHD without ADHD. The lack of association with ADHD without DBDs is based on a well powered GWAS, which has a higher effective sample size than the primary GWAS meta-analysis (Neff ADHD without DBDs=33,613 vs Neff ADHD+DBDs meta-analysis = 13,559). In our opinion these powerful analyses are valid in order to support our conclusion of the chromosome 11 locus mainly being a risk locus for ADHD+DBDs.

We have added information about the Chinese cohort, which is Han Chinese (as assumed by the reviewer), on page 6.

We have used PLINK for the GWAS association analyses and added this information on page 18.

Further although a GWAS of the Chinese sample was performed, no details of the QC or results are presented (i.e. genotype QC particularly with respect to MAF, as well as lambda, QQ-plot, rg with European cohorts, whether any genome-wide significant loci were observed). beyond the results of the three loci significant in the European-only meta-analysis. Although, given the small size of this cohort, any significant hits are unlikely, these data would provide confidence in the Chinese sample and improve interpretation of the meta-analysed results.

We have specified on page 17 that the QC procedure described for the PGC cohorts also applies to the Chinese cohort.

We have also included additional information about the variants going into the primary GWAS meta-analysis of ADHD+DBDs and the trans-ancestry GWAS on page 18: "In the two meta-analyses only variants with MAF > 0.01 and imputation INFO score > 0.8 were included. All variants that were not supported by an effective sample size of 70% in the meta-analysis outputs were filtered out."

As expected, there are no genome-wide significant findings in the GWAS of the Chinese cohort due to the low sample size (406 cases and 917 controls). Which is also clear from the qq-plot which we have added to the supplementary information (Supplementary Figure 5, referred to in the main text on page 6). This figure also gives information about lambda (λ=1.015).

Since we do not have access to a merged data set of genotypes from the European and Chinese cohorts it is not possible for us to estimate the genetic correlation based on genotypes (which usually performs better when having small sample sizes as is the case for the Chinese cohort). Estimation of the genetic correlation based on summary statistics (e.g. using POPCORN, Brown et al. 2016) does not give reliable results due to the small sample size of the Chinese cohort. Instead we have added results from heterogeneity test across the European cohorts and the Chinese cohort (tested with Cochran's Q test and quantified with the I^2 heterogeneity

index) in Supplementary Figure 6 and 7 (Manhattan and qq-plots). The results indicate no signs of heterogeneity among cohorts. This information has been added to the main text page 6: "(no variants demonstrated significant heterogeneity across European and Chinese ancestries, Supplementary Figure 6 and 7)".
Similarly LocusZoom plots, or similar, of the regions spanning the three GWAS significant loci in the meta-analysed European and separately the Chinese cohort would be informative, as would the MAF of the loci in the Chinese sample. The authors should consider applying the trans-ethnicity fine-mapping approach published by Lam et al, PMID: 31740837 to refine there top three loci.

Locus zoom plots of the three genome-wide significant loci are already present in the Supplementary Information (Supplementary Figure 1.A-C). We have added regional association plots for the three loci in the Chinese cohort in Supplementary Figure 8.A-C, and added information about the figures on page 6: "The locus on chromosome 11 was nominally significant in the Chinese cohort (P=0.006, Supplementary Figure 8.C)."

We agree with reviewer that ancestry heterogeneity could be an issue, as some causal variants, have varying allele frequency and linkage disequilibrium (LD) patterns such that associations that can be detected in one population may not be readily detected in others. The fine mapping approach suggested by Lam et al. aims at fine map risk loci to decrease the number of potential causal variants in identified risk loci, by including information across ancestries. The premise in the method is that genetic effects are highly consistent across populations, and that the causal variants will have consistent effects across populations, whereas non-causal variants can have inconsistent effects due to population-specific LD patterns. They therefore expect causal variants to have greater statistical significance and less heterogeneity in their trans-ancestry meta-analysis compared with other alleles that are indirectly associated via LD. Lam et al. only apply their method to genome-wide significant loci because the power to fine-map signals beyond that is limited due to the sample size for Asian schizophrenia cases in their study (22,778 schizophrenia cases and 35,362).
In this study, the sample size of the Chinese cohort (406 cases and 917 controls) is too underpowered for providing any valuable information for disentangling the causal variants in the chromosome 11 locus from non-causal LD variants, using the method suggested by Lam et al. However, we have no reason to suspect that large differences in MAF and LD create heterogeneity across Europeans and Chinese ancestries at the chromosome 11 locus. When looking at the allele frequency of the affect allele of the index variant it is 0.51 in Europeans and 0.48 in the Chinese. Additionally, when inspecting the regional association plots visually (Supplementary Figure 1.C and Supplementary Figure 8.C) the LD patterns are similar across the two ethnicities with the signal covering most of *STIM1* and *RRM1*.

We have added a column in Table 1 with information about MAF in controls in the European and Chinese cohorts.

The phrase "represent a more severe phenotype with respect to the genetic risk load" needs to be revised. The severity of any disease phenotype is independent of the underlying genetic load. Although the authors have shown a higher loading of common risk alleles associated with ADHD in the ADHD+DBDs sample, they show no evidence of a more severe phenotypes in terms of symptoms.

We agree with the reviewer that an increased load of common risk variants does not give information about the severity of the phenotype. We have revised this statement in the abstract which now reads: "Our results suggest that the ADHD+DBDs phenotype have an increased genetic risk load with respect to common variants compared to ADHD without DBDs, in line with previous studies, and that the risk load to some extent can be explained by variants associated with aggressive behavior."

Similarly the evidence that the cohort of ADHD+DBDs show an enrichment for "loci which are distinct to ADHD+DBDs" is unclear. Could the authors clarify the evidence to which they refer.

This sentence refers to the combined observation of single locus and polygenic differences between ADHD+DBDs compared to ADHD without DBDs. The chromosome 11 locus seems to be specific to ADHD+DBDs as this locus is genome-wide significantly associated with ADHD+DBDs (OR=1.16, P= 8.97x10-9), but when excluding comorbid individuals in the well-powered ADHD without DBDs GWAS (13,583 cases and 22,314 controls) the signal completely disappears (OR=1.022, P=0.175). Based on these results (and additional validation when considering the results from the mtCOJO analysis and the GWAS of ADHD+DBDs vs ADHD-only) we think we have enough support to claim that the chromosome 11 locus seems to be distinct to ADHD+DBDs.
Additionally, when we test the genes most strongly associated in the primary ADHD+DBDs GWAS meta-analysis (Pgene<10^{-3}, 79 genes) in the two secondary GWASs, we find the set significantly associated with ADHD without DBDs (beta=0.312(SE=0.02), P=$9x10^{-4}$), which is also expected since the comorbid cases are diagnosed with ADHD. However, in the gene-set analysis where we test for association of the gene-set with ADHD+DBDs compared to ADHD without DBDs (thus adjusting for the effect of ADHD) we find a much stronger association (beta=1.1 (SE=0.07), P = $9.28x10^{-32}$). This very strong association must be driven by variants that associate much stronger with the comorbid phenotype rather than ADHD without DBDs.

However, we acknowledge that some of the polygenic differences between ADHD+DBDs and ADHD without DBDs could be driven by an enrichment of risk variants with pleiotropic effects in ADHD+DBDs (rather than ADHD+DBDs specific risk variants). So in order to make it clear that only a part of the polygenic differences could be due to ADHD+DBDs specific risk variants we have modified the sentence in the summary, page 15, which now reads: "Individuals with ADHD+DBDs therefore represent a phenotype with an increased genetic risk load compared to ADHD without DBDs, including variants which seem distinct to ADHD+DBDs."

Can the authors comment on whether results best fit a model where ADHD+DBDs is a subtype of ADHD or individuals co-morbid for two genetic disorders that overlap in their genetic risk factors.

This is a very interesting and relevant question. However, in order to be able to answer this it would require data from a large genotyped sample of individuals with DBDs without ADHD. Unfortunately, we do not currently have that.
We do have some results that support partly genetically distinct (but most likely highly genetic correlated) phenotypes. We find a significant SNP-heritability of ADHD+DBDs compared to ADHD without DBDs (h^2-snp=0.08) and in the PRS analyses, where we test for association of PRSs with ADHD+DBDs when compared to ADHD without DBDs, we find that ADHD+DBD cases are significantly enriched for PRSs of seven phenotypes including

aggression and phenotypes related to cognition. These results support that the ADHD+DBDs phenotype is significantly different at the polygenic level compared to ADHD without DBDs, and it could be speculated that some of this heterogeneity is driven by DBD specific risk variants. However, this is speculative since we do not have the data to finally conclude this.

Further comments (in manuscript order)
1) Throughout the English and grammar needs to be checked closely, including the supplementary information. Examples of poor sentence construction/errors include:
i) Page 3 paragraph 1: correct "of of",
replace "like" with "such as",
replace "around" with "approximately",
replace "higher mortality rates than in individuals with ADHD" with "higher mortality rates than in ADHD",
Replace "Among those with DBDs in the same Danish cohort, more than half (57%) also had a comorbid diagnosis of ADHD" with "Among those with DBDs in that same Danish cohort, more than half (57%) had a comorbid diagnosis of ADHD."
replace "only diagnosed with ADAD" with "diagnosed with ADHD only".

ii) Page 3 paragraph 2: replace ADHD as well as DBDs" with "ADHD and DBDs".

We thank the reviewer for pointing us to the errors in the manuscript and the suggested edits listed in point i-ii. Those have been corrected the in revised version of the manuscript.

iii) Page 4 Paragraph 3 and elsewhere: consider replacing the term "generalization", its meaning is unclear.

We thank the reviewer for pointing this out. We have used the word “generalization” in relation to the GWAS meta-analysis of European and Chinese cohorts at several places in the manuscript (page 4, 11 and 18). We have replaced “generalization” with “trans-ancestry” which is a more precise term.

iv) replace "conductict" with "conduct".
v) Page 9 paragraph 2: replace "compared to the GWAS of ADHD without DBDs" with "compared to ADHD without DBDs".
vi) Page 10 paragraph 2 is missing a P= for the results from educational attainment.
vii) Page 11 paragraph 2: replace ".;" with "."
viii) Page 13 paragraph 2: replace "to mainly to" with "to mainly"
ix) Page 13 paragraph 3: replace "Supplementary Table 5" with "Supplementary Table 6"

We thank the reviewer for pointing us to the errors in the manuscript and the suggested edits listed in point iv-ix. Those have been corrected in the revised version of the manuscript.

x) Page 14 Paragraph 1: replace "Going beyond ADHD risk burden, the common variant component of DBDs could also include variants distinctly associated with aggression. This idea is supported by our finding of increased PRS for aggression in ADHD+DBDs compared to ADHD without DBDs..." with "Going beyond ADHD risk burden, the common variant component of DBDs could also include distinct variants associated with aggression only. This idea is supported by

our finding of increased PRS for aggression in children in ADHD+DBDs compared to ADHD without DBDs..." However, no evidence is provided that this increase in load is not due to variants with pleiotropic effects on ADHD and aggression in children.

We thank the reviewer for the suggestion regarding the sentence on page 14, and we have revised it accordingly, but excluded the word "distinct" (see below).
We agree with the reviewer that we do not know if the increased load of aggression associated variants in ADHD+DBDs cases compared to ADHD without DBDs, could be due to an increase in variants with pleiotropic effects on ADHD and aggression. We have therefore modified the sentence on page 14, which now reads: "Going beyond ADHD risk burden, the common variant component of DBDs could also include variants mainly associated with aggression."
If it is the case that pleiotropic variants rather than distinct aggression specific variants are enriched in ADHD+DBDs (compared to ADHD without DBDs) the PRS result still supports our main conclusion that ADHD+DBDs cases have an increased load of variants associated with aggression.

xi) Page 15 paragraph 1: replace "idea is supported by" with "idea is consistent with".

xii) Page 19 paragraph 2 : replace "correct out" with "adjusted for".

xiii) Supplementary figures 1, 3, 5 and 6 replace "vertical" line with "horizontal" line, when referring to the genome-wide significant threshold. And the opposite for Supplementary Figure 2A i.e. replace "vertical lines" with "horizontal bars" represent SE.

xiv) Elsewhere in the supplementary information requiring correction:
"Two-sided P-values represents tests for the difference of the genetic correlations from zero."
"Distribution DBDs over ICD-10 codes among cases with ADHD+DBDs in the iPSYCH cohort"
"..and thier association P-values (P) odds ratio (OR) and standard error (SE).."
"the 95% confidence band."

NB. This is not an exhaustive list, there may be other errors.

We thank the reviewer for pointing us to the errors in the manuscript and the suggested edits listed in point xi-xiv. Those have been corrected in the revised version of the manuscript.

2) Page 7 paragraph 3: Refers to Supplementary Table 9 which states rs549845*: *This locus is genome-wide significantly associated with ADHD without DBDs, however with another index variant (rs113551349, P=3.74x10-10, OR=0.89). The table shows that the p-value for ADHD without DBDs is 8x10-8
i.e. NOT genome-wide significant. This also raises the question as to how the loci were selected for this analysis. How was a significant difference in the effect of these loci in the different groups assessed i.e. what statistical test was performed.

In Supplementary Table 1 (previously Supplementary Table 9), we are listing P-values from the two secondary GWASs ("ADHD+DBD vs. ADHD-only GWAS" and "ADHD without DBDs) of the three index variants that are genome-wide significant in the GWAS meta-analysis of ADHD+DBDs. This means that the only selection of variants that have occurred is

extraction of P-values in the secondary GWASs of the three index variants from the primary GWAS meta-analysis.
As a service to the reader we note below the table that the locus on chromosome 1 is genome-wide significant in the "ADHD without DBDs GWAS", however with another index variant. This result support our conclusion that the chromosome 1 locus mainly reflects association with ADHD.

We thank the reviewer for questioning how we have tested for difference effect sizes. Due to sample overlap between the GWAS meta-analysis of ADHD+DBDs and the GWAS of ADHD without DBDs (~13,500 controls from iPSYCH are included in both analyses), it is not possible to test for significance between effect sizes due to violation of the assumption of independent samples. We have deleted the word "nominal" in the sentence on page 7, which wrongly indicated that we had tested for significance. The sentence now reads: Of the significant loci from the GWAS of ADHD+DBDs, the loci on chromosomes 7 and 11 had stronger effect sizes in the ADHD+DBD vs. ADHD-only GWAS compared to the ADHD without DBDs GWAS.

3) The mtCOJO analyses do not confirm or refute a significant difference in effect in ADHD versus DBDs.

The reviewer is right about this observation. This analysis is included as a secondary analysis in order to further elucidate the findings from the primary and secondary GWASs. In the mtCOJO analysis we see a decrease in association of all three genome-wide significant loci, which is expected since the cases all have ADHD. However, we also observe that when adjusting for the effect of ADHD without DBDs the associations follow the same pattern observed from the other analyses – that the chromosome 11 locus remains the most strongly associated and the chromosome 1 locus seems mainly to be driven by ADHD.

4) Please give a reference for the jackknife method mentioned on Page 10 paragraph 1.

We have inserted the relevant reference on page 10.

5) Consider revising the sentence "Our results suggest that the aggressive and disruptive behavioral component of the ADHD+DBDs phenotype has a biological cause..". Surely that is a fact rather than a conclusion from their data.

We agree with the reviewer that the sentence is unclear. Aggression, which is a part of the clinical characterization of ADHD+DBDs, could be caused by both environmental and genetic (biological) factors. Our results suggest that individuals with ADHD+DBDs to some extent are predisposed genetically to aggression and that the aggressive part of the phenotype is influenced by genetic variants associated with aggression in the general population. We have rewritten the sentence on page 15 to make it clearer: "Our results suggest that the aggressive and disruptive behavioral component of the ADHD+DBDs phenotype has a genetic risk component, which in part include common risk variants associated with ADHD, aggressive and anti-social behavior."

6) Page 17 Quality control and imputation. Please give the post imputation QC filters applied to the

datasets used for the GWASs i.e Imputation quality, MAF, call rates etc. Was the Chinese sample imputed, if so, what reference panel was used.

We have included information about MAF and imputation INFO score on page 18: “In the two meta-analyses only variants with MAF > 0.01% and imputation INFO score > 0.8 were included. All variants that were not supported by an effective sample size of 70% in the meta-analysis output were filtered out.”

The Chinese sample was imputed using the 1000 genomes phase 3, East Asian reference genome. This information was added on page 18.

7) Page 18 paragraph 2: include the covariates used in Supplementary Table 2.

We have added the requested information to Supplementary Table 9 (Previously Supplementary Table 2).

8) PRS analyses, R2 based on the liability scale should be calculated for the binary traits i.e. psychiatric disorders.

When converting R^2 to the liability scale, it is with respect to the liability of the target phenotype (ADHD+DBDs here), which means that this can be done for both the binary and continuous traits investigated. We are aware that reporting Nagelkerkes R^2 on the observed scale does not take prevalence of the disorder and proportion of cases in the study into account. This is especially problematic in studies comparing variance explained by PRS between studies with varying case/control ratios and phenotype prevalence. In the PRS analysis in this study we have estimated variance explained (by PRS for different phenotypes) in the same set of individuals, so different case/control ratios and prevalences do not affect the estimates.
However, in order to meet the request from the reviewer we have added information about Nagelkerke´s R^2 on the liability scale. The conversion was done using a prevalence of 0.2 of ADHD+DBDs among ADHD cases and the method suggested by Lee et al. Genetic Epidemiology 2012. The results have been added to Supplementary Table 7.
We have added information about this on page 23: ”For each P-value threshold the variance in the ADHD+DBDs phenotype explained by PRS was estimated using Nagelkerke’s R^2 (R package ‘BaylorEdPsych’) and conversion to Nagelkerke’s R^2 on the liability scale was done using the method suggested by Lee et al.[84] and a prevalence of 0.2 for comorbid DBDs among ADHD cases.”

9) Consider unifying how "ADHD without DBDs" is named. There is "ADHD without DBDs", "ADHD-only", "ADHDwoDBDs" within the manuscript.

We have corrected ADHDwoDBDs to “ADHD without DBDs” where it occurred. The term ADHD-only is used to refer to the case-only GWAS of ADHD+DBDs vs ADHD cases without comorbid DBDs. We refer to this as the “ADHD+DBD vs. ADHD-only” GWAS. We will correct this to “ADHD+DBDs vs ADHD without DBDs GWAS” if the editor and reviewer prefer this name. However, we think this name is too long so we have not corrected it in the revised version of the manuscript.

Reviewer #2 (Remarks to the Author):

This report summarizes findings from a genome-wide association study of ADHD+disruptive behavior disorders. The analyses follow a conventional pipeline in the field of psychiatric genetics, and so although the methods are not particularly novel in themselves do provide novel information regarding the genetic architecture of ADHD+DBS.

My only concern with the manuscript is that the selection of the Chinese replication cohort needs additional justification, as it seems the authors would find better-matched cohort to the Danish discovery sample. I would find the replication effort to be more convincing with a more closely matched validation sample.

To our knowledge there are no large European cohorts with ADHD+DBDs data currently available for inclusion as replication in this study.
In this study we have included all available cohorts with ADHD+DBDs into the primary GWAS meta-analysis of ADHD+DBDs to maximize power. Subsequent evaluation of the distribution of effect sizes for the three genome-wide significant loci in the seven included cohorts revealed the same direction of association in all cohorts for the three index variants (Supplementary Figure 2.A-C.). For the chromosome 11 locus, which is the one most strongly associated with the comorbid phenotype, the index variant is nominally significantly associated in four out of the six cohorts. The results support that the associations of the three loci are true associations rather than spurious signals driven by strong association in one cohort.
The inclusion of the Chinese cohort is not only a replication effort but primarily for identification of trans-ancestry risk loci across European and Chinese cohorts. Additionally, it should be mentioned that we did not observe any variants demonstrating significant heterogeneity across European and Chinese cohorts (Supplementary 6 and 7).

Reviewer #3 (Remarks to the Author):

This paper reports a GWA of ADHD+DBD (‘A+D’ below) for 3802 cases from 7 cohorts, which combines samples from the Danish iPsych study and the PGC. The authors claim that this is the largest GWA study for A+D, although it is much smaller than the largest GWA study of ADHD.

Three GWA-significant associations, one of which on Chromosome 11 emerged both for European and Chinese samples, but not for the other two associations. Moreover, the C-11 association was not found in a secondary GWA analysis of ‘pure’ ADHD cases*.

(see reply to * below)

The authors conclude that this C-11 association is specific to the comorbid phenotype. However, this locus has not previously been implicated in ADHD, DBD, aggression or other psychiatric disorders.

The authors report that SNP heritability is greater for A+D than for ADHD without DBD (‘A-D’

below) in the iPSYCH cohort, although SNP heritability for A+D was lower in the whole sample. **

(see reply to ** below)

I was confused by this sentence on page 9: "Additionally, common variants explained a small fraction of the variation in the ADHD+DBDs phenotype compared to the ADHD without DBDs phenotype (h2SNP=0.08; SE=0.04, GCTA estimate) (Supplementary Table 6).

"They also report high genetic correlations between A+D and aggressive behaviors (rg = .81) and antisocial behaviors (rg = .82), although A-D was also correlated genetically with aggression (rg = .74).***

(see reply to * below)**

The authors report polygenic score analyses indicating that polygenic scores for ADHD and for aggression correlated more highly with A+D than with A-D.****

(see reply to ** below)**

On the basis of these findings, the authors conclude that A+D has a more severe genetic risk load than A-D and suggest that this might in part be due to A+D links with aggressive behavior. I wasn't convinced by these data that there is much merit in the A+D concept, but it is noteworthy that these suggestive results correspond with twin studies that suggest that A+D is more heritable than A-D and that A and D have only moderate genetic overlap. Also, polygenic score analyses yield higher correlations with A+D than with A alone.

Although the N of 3802 cases is the largest GWA study of A+D, isn't it the case that other disorders didn't reach genome-wide significant results until the N was much larger?

The comorbid phenotype analyzed here is severe and relatively well-defined and we expect the biological risk factors to be rather homogenous across cases (compared to e.g. more heterogeneity across the full ADHD spectrum), which increases power to detect associated loci.
For comparison some GWASs of psychiatric disorders have identified genome-wide significant loci at a comparable size as ours e.g. Duncan et al. American Journal of Psychiatry, 2017, identified one genome-wide significant locus for anorexia nervosa based on 3,495 cases and 10,982 controls. Additionally we have previously reported one genome-wide significant loci for cannabis use disorder (which was replicated in an independent sample) based on 2,387 cases and 48,985 controls (Demontis et al. Nature Neuroscience 2019).

Some of my comments follow from these findings, as asterisked above, most of which have to do with avoiding false positive results. (I assume that none of these results were preregistered hypotheses.)

*I wasn't clear that this was a significant difference. (Finding that the association was significant in one analysis but not in the other does not mean that this is a significant difference.)

It is not possible to test for statistical significance between the effect sizes from the GWAS of ADHD+DBDs vs the effect size form GWAS of ADHD without DBDs, since the two analyses include overlapping samples (~22,000 iPSYCH controls are included in both analyses). Instead we have evaluated our findings from the primary GWAS meta-analysis in the two secondary GWASs. When evaluating the genome-wide significant locus at chromosome 11 identified in the primary GWAS meta-analysis of ADHD+DBDs (3,802 cases and 31,305, P=8.97x10-9, OR=1.164) in the GWAS of ADHD without DBDs the locus is not associated (13,583 cases and 22,314 controls, P=0.175, OR=1.022). The secondary GWAs has great power to detect associated loci and in fact has a higher effective sample size (N_{eff}) than the primary GWAS meta-analysis (N_{eff} ADHD without DBDs=33,613 vs N_{eff} ADHD+DBDs meta-analysis = 13,559). Which supports that the chromosome 11 locus is associated with the comorbid phenotype rather than ADHD without DBDs. A conclusion that is reinforced by the findings in the analyses where we adjust for the effect of ADHD. In the ADHD+DBDs vs ADHD-only GWAs and the mtCOJO analysis the effect sizes only decrease slightly and the chromosome 11 locus remains strongly associated with ADHD+DBDs (ADHD+DBDs vs ADHD-only GWAs: OR=1.13, P=7.07x10-4; mtCOJO: OR=1.14, P=1.43x10-6).
So even though it is not possible for us to test directly for significant difference in effect sizes we provide strong evidence suggesting that the chromosome 11 locus is a risk locus for ADHD+DBDs rather than for ADHD without DBDs.

**Is this a significant difference in SNP heritability? The standard errors suggest that it is.

We thank the reviewer for pointing this out, and we agree that this information is important. In order to be able to test for difference in h^2_{SNP} between ADHD+DBDs and ADHD without DBDs we need h^2_{SNP} estimates based on independent samples (~22,000 iPSYCH controls overlap between analyses). We have therefore included GCTA analyses of independent iPSYCH samples in the revised version of the manuscript and subsequently tested for difference in h^2_{SNP}. The estimates are significantly different as expected by the reviewer (results can be found in Supplementary Table 5.B).
We have added information about this in the method section on page 22: "In order to be able to test for difference in h^2_{SNP} between ADHD+DBDs and ADHD without DBDs we re-estimated h^2_{SNP} using GCTA based on independent iPSYCH controls. For this analysis the iPSYCH controls were split randomly into two groups within each genotyping wave. One group was used as controls for estimating h^2_{SNP} of ADHD+DBDs (2,155 cases and 11,659 controls) and the other group was used to estimate h^2_{SNP} of ADHD without DBDs (13,583 cases and 11,250). The analyses using independent controls were done using prevalences of 2% and 1% for ADHD+DBDs and 3% and 4% for ADHD without DBDs. Test for difference in h^2_{SNP} between ADHD+DBDs and ADHD without DBDs was done using:

$$Z_{diff} = (h^2_{SNP(ADHD+DBDs)} - h^2_{SNP(ADHD\ without\ DBDs)}) / sqrt(SE^2_{(ADHD+DBDs)} + SE^2_{(ADHD\ without\ DBDs)})$$

where Z_{diff} is the Z score for the difference in h^2_{SNP} and SE is the standard errors for the heritabilities. We calculated two-tailed P-values in R. "

We have added the following to the result section on page 9: "The h^2_{SNP} estimate was significantly higher for ADHD+DBDs compared to ADHD without DBDs in GCTA analyses of independent iPSYCH samples, with $P_{difference}$= 5.36x10^{-6} when assuming a prevalence of 2% for ADHD+DBDs and 3% for ADHD without DBDs (Supplementary Table 5.B)."

The reviewer also note that the h^2_{SNP} estimate is lower for the GWAS meta-analysis of ADHD+DBDs compared to the estimate based only on the iPSYCH cohort. In the meta-analysis several smaller cohorts are included, in such cohorts the size of the error terms in the SNP effect estimates will be larger (compared to a large single cohort e.g. the iPSYCH cohort), which will introduce heterogeneity at the polygenic level into the results and bias the h^2_{SNP} heritability downwards.

***More fundamentally, what is the genetic correlation between A+D and A-D?

We thank the reviewer for noticing that this information is missing in the manuscript, and we agree that inclusion of this could be is informative as a sanity check of the analyses as we would expect this to be close to one as all cases in the two analyses are diagnosed with ADHD. We have added this information on page 10: Additionally, as a sanity check we estimated the genetic correlation between ADHD+DBDs and ADHD without DBDs. As we expected this correlation was close to one as all cases are diagnosed with ADHD (r_g=0.99, $2.64x10^{-45}$).

****Was this a significant difference?
Most important for me is to know how predictive any of these polygenic scores are with ADHD-related behaviors in independent samples?

All the PRSs were trained in (that is, weights obtained from) independent samples (the GWASs listed in Supplementary Table 7) and the scores generated for the individuals in the current study. That is, we investigate how PRSs trained in independent sample sets with relevant phenotypes associate with (/predict) A+D comorbidity in our case-only design.
The GWAS results of our study will provide the opportunity for testing how PRS trained in our GWAS may associate with (/predict into) other samples with ADHD-related behaviors by researchers that have access to individual level genotype data from such samples. We do not have access to genotype data from other samples and it is not within the scope of the current study. Although our study is the largest GWAS of diagnosed DBD so far, it is still relatively limited in size with a moderate polygenic signal and, accordingly, the predictive power of (A+D) PRS using our GWAS results would be expected to be relatively modest.

If I understand the results on page 10, it appears that the effect sizes (OR, R2) are weak. How do these effect sizes compare to those from previous ADHD-related GWA studies. Do any of these polygenic scores (e.g., A, A+D, A-D) improve predictive power?

Yes, the effect sizes are indeed limited. This is expected because the analysis is a case-only study contrasting two related phenotypes (A+D cases versus A-D cases). Thus, it is not directly comparable to e.g. the recent ADHD GWAS (Demontis et al, Nat Genet 2019) showing substantially larger effect sizes of ADHD-PRS association with ADHD caseness (OR = 1.56 and R^2 = 5.5%). Additionally, we would like to note that the PRSs tested are derived based on results from external GWASs of related phenotypes (listed in Supplementary Table 7) so we did not test PRS for A+D and A-D (those GWASs are still quite underpowered for as training GWASs for PRS analyses).

I wasn't clear about the statement on page 10: "Only a small proportion of the variance in ADHD+DBDs (compared to ADHD without DBDs) was explained by the PRSs, with the

maximum Nagelkerke's R2 = 0.36% for the ADHD PRS." Does this mean that less than 1% of the variance was explained by the A+D polygenic scores? If not, could the authors include some clear statements as to the variance explained by the A+D polygenic score (as compared to other polygenic scores) in predicting ADHD-related behaviors in independent samples? If, as it seems to me, the present GWA analyses yield polygenic scores that predict less variance than previous studies, can the authors explain this?

It is correct that less than 1% of the variance in A+D compared to A-D is explained by ADHD(-trained) PRS. However, to be clear, it is not the proportion explained by *A+D PRS*. In fact, we do not generate any A+D PRS in the current study.
As mentioned above, the PRS analysis is conducted in a case-only design contrasting two closely related phenotypes and the R^2 values presented are therefore lower than – and not directly comparable to – R^2 values obtained in large case-control studies.

A general issue is that other GWA studies of ADHD-related behaviors (including aggression) are about to be published. However, I believe that these involve dimensional traits rather than dichotomous diagnoses. Are the authors aware of impending GWA publications of ADHD-related behaviors? If so, it would be good to future-proof this paper a bit so that it is not superseded by the time it is published.

We are aware of GWASs of related phenotypes e.g. Zayats et al. is working on a GWAS meta-analysis of ADHD symptom scores in the general population and Dorret Boomsma and colleagues are close to publishing a GWAS of childhood aggression based on phenotype measures across different cohort (manuscript available on bioRxiv). The phenotype analyzed in this study is very different from those related phenotypes as our cases are clinically diagnosed and represents the extreme of the phenotype distribution with respect to aggression, impulsive and inattentive behaviors. The results from these studies are therefore not redundant, in contrast they help to give us a better understanding of the complex genetic architecture underlying ADHD and aggressive behaviors in the general population as well as in clinically diagnosed individuals.

The authors don't mention 'p', the current notion of genetic overlap among many disorders. Given that they are arguing for a genetic correlation between ADHD symptoms and disruptive symptoms, the authors might wish to say something about 'p'.

In this study we are not aiming at identifying the genetic correlation between ADHD symptoms and disruptive symptoms, which is not possible because we do not have GWAS results of "disruptive symptoms" derived from DBDs without ADHD. However, it might be that the reviewer is referring to the genetic correlations of ADHD (with and without DBDs) with aggression and anti-social behavior in the general population?
We agree with the reviewer that researchers recently have reconceptualized psychopathology using transdiagnostic approaches and higher-order dimensions, including externalizing and internalizing dimensions and at times a general factor ("p"), as an alternative to traditional diagnostic categories. Although this "p factor" is heritable and related to many important correlates, researchers have questioned its validity on statistical grounds. Thus, while our findings on the genetic basis of ADHD + ODD/CD reflect the higher-order externalizing dimension in children and adolescents, it remains to be seen whether they also reflect "p". We

think (even though it is an interesting topic), that it is out of the scope of this study to elucidate this point.

The paper is well organized and clearly and succinctly written, despite the complexity of the analyses.

We thank the reviewer for this positive comment on our study.

Reviewers' comments:

Reviewer #1 (Remarks to the Author):

The manuscript is improved over the previous version and many of my comments have been addressed appropriately or further explanation has clarified the previous statements. This is and interesting paper both to for the psychiatric genetics community but also for broader studies of co-morbidity.

I have no remaining issues with the analyses but three major concerns over how those results are reported.

I was concerned that some of the statements were over stated or the statistical evidence lacking. Rereading the manuscript in its current form it clear that the secondary GWAS described as "ADHD+DBDs vs ADHD-only" actually provides much of the evidence that I was seeking and that it is how these results are reported in the text makes this unclear.

ADHD+DBDs vs ADHD-only is a case-only analysis comparing ADHD cases with DBDs to ADHD cases without DBDs. This directly tests whether there is a difference in the effect of an allele in ADHD cases with and without DBDs. However it is reported as if the importance is the relative size of this effect compared to the size of the effect in ADHD without DBDs, rather than the key being the direction of the difference in effect and its significance.

For example: Page 7 paragraph 3
"Of the significant loci from the GWAS of ADHD+DBDs, the loci on chromosomes 7 and 11 had stronger effect sizes in the ADHD+DBDs vs. ADHD-only GWAS compared to the ADHD without DBDs GWAS....(Supplementary Table 1)"

It would more naturally be reported as:
Of the significant loci from the GWAS of ADHD+DBDs, the loci on chromosomes 7 and 11 show larger effect sizes in the ADHD+DBDs analyses (ORs 1.199 & 1.164 respectively)compared to the ADHD without DBDs GWAS (ORs 1.071 & 1.022 respectively), and the case-only analyses confirm that there is a significantly stronger effect of the risk allele in ADHD+DBDs cases compared to ADHD without DBDs cases (ORs 1.128 & 1.126 respectively).

This odd emphasis is repeated throughout the manuscript where the results of the case-only analyses are reported or discussed:
Page 8 paragraph 3
Page 9 Paragraph 2
Page 12 Paragraph 1
Page 13 bottom

Secondly, it would greatly aid the reader if the authors used the term "ADHD-DBDs" rather than "ADHD without DBDs". Similarly, the case-only analyses could be described as "case-only analyses of DBDs" or "ADHD+/-DBDs analyses" rather than "ADHD+DBDs compared to ADHD without DBDs". These should be used consistently including replacing "ADHD alone" and "ADHDwoDBDs"; as seen on page 8 and supplementary table 6.

Thirdly, Page 15 replace "underlying biological cause" with "underlying genetic cause". This follows my comments on the previous draft and I had hoped that this had been corrected. Could the authors also agree replace "distinct" with "specific" on Page 16?

Additional comments:

i) In the abstract the authors state that" Genetic correlatins of ADHD+DBDs with aggressive (rg=0.81) and anti-social behaviors (rg=0.82)were high....." This is true but they not significantly higher when the two iPSYCH subsamples were compared (Page 10 paragraph 1, rg =0.85 & 0.92 in ADHD+DBDs versus rg = 0.74 & 0.56). So this statement may be misleading and should be rephrased.

ii) page 6
replace "Chines" with "Chinese"
replace "chromosome 11 locus to be a risk locus" with "chromosome 11 locus is a risk locus"

iii) The gene set test is described as using 79 genes on page 8 and 80 genes on page 20. Please check. Could you also report the results for all the genes used in this gene-set test in Supplementary Table 3.

iv) On page 9, consider using "Three genes were predicted to be differentially expressed" instead of "Three genes were predicted differentially expressed". It would also be useful to specify the brain regions here, although they are reported in the Supplementary table.

v) Page 9 SNP heritability. Can the authors explain why multiple prevalences were tested for the SNP heritability and where the different values came from.

vi) Page 10 paragraph 1. It should be made clear that the ADHD without DBDs analysis reported was tested analysing only the iPSYCH cohort.

vii) Please correctly reference the jackknife method used as Shao & Wu, 1989 as implemented in LDSC. https://jstor.org/stable/2241717

viii) Page 11. I can see why the authors have reported the age at first birth results as they do, but it is more correct to say that the association is a negative association with age at first birth and then, as they do in the discussion, suggest that this likely reflects ADHD+DBDs individuals as having children at a younger age. Otherwise the stated result looks at odds with the results in Suppl Table 7 and Suppl Figure 11.

ix) Page 13 replace "calcium signaling to be" with "calcium signaling is"

x) Page 13 replace "Six genes were exome wide" with "In gene-based analyses, six genes were"

xi) Page 15 replace "to play" with "play" and replace "of getting a" with "of a"

xii) Page 15 Replace "We identified individuals with ADHD+DBDs to have increased" with We have shown that individuals with ADHD+DBDs have an increased"

xiii) Consider rephrasing:

Methods pages
"Register register"
"aggregated by PGC" perhaps "provided by the PGC"
"preferred kept" with "preferentially retained"
"GWAS GWAS"
"acrosse"

Figure 2
"Freqeuncy"

Reviewer #3 (Remarks to the Author):

The authors have assiduously addressed my comments. For example, they conducted new analyses using iPSYCH to test for SNP heritability differences between ADHD+DBD vs ADHD-DBD, which they now show are significantly different.

One remaining concern is relevant to the authors' main argument that A+D has a more severe genetic risk load than A-D and suggest that this might in part be due to A+D links with aggressive behaviour. However, both A+D and A-D are genetically correlated with aggressive behaviour (.82 vs .74, not significantly different).

For this reason, I asked to see the genetic correlation between A+D and A-D. The authors report that the genetic correlation is 0.99. The authors say this was expected because all cases are diagnosed with ADHD. But isn't the point of the paper that ADHD with DBD differs genetically from ADHD without DBD? If the genetic correlation is 0.99, doesn't this mean that there is no difference genetically?

Reviewers' comments:

Reviewer #1 (Remarks to the Author):

The manuscript is improved over the previous version and many of my comments have been addressed appropriately or further explanation has clarified the previous statements. This is and interesting paper both to for the psychiatric genetics community but also for broader studies of co-morbidity.

We thank the reviewer for this positive comment on the revised version of our manuscript.

I have no remaining issues with the analyses but three major concerns over how those results are reported.

I was concerned that some of the statements were over stated or the statistical evidence lacking. Rereading the manuscript in its current form it clear that the secondary GWAS described as "ADHD+DBDs vs ADHD-only" actually provides much of the evidence that I was seeking and that it is how these results are reported in the text makes this unclear.

ADHD+DBDs vs ADHD-only is a case-only analysis comparing ADHD cases with DBDs to ADHD cases without DBDs. This directly tests whether there is a difference in the effect of an allele in ADHD cases with and without DBDs. However it is reported as if the importance is the relative size of this effect compared to the size of the effect in ADHD without DBDs, rather than the key being the direction of the difference in effect and its significance.

For example: Page 7 paragraph 3
"Of the significant loci from the GWAS of ADHD+DBDs, the loci on chromosomes 7 and 11 had stronger effect sizes in the ADHD+DBDs vs. ADHD-only GWAS compared to the ADHD without DBDs GWAS....(Supplementary Table 1)"

It would more naturally be reported as:
Of the significant loci from the GWAS of ADHD+DBDs, the loci on chromosomes 7 and 11 show larger effect sizes in the ADHD+DBDs analyses (ORs 1.199 & 1.164 respectively)compared to the ADHD without DBDs GWAS (ORs 1.071 & 1.022 respectively), and the case-only analyses confirm that there is a significantly stronger effect of the risk allele in ADHD+DBDs cases compared to ADHD without DBDs cases (ORs 1.128 & 1.126 respectively).

When reading through our response to the comments, please note that the terms used to describe the two secondary GWASs have been changed (see reviewer point further below).

We agree with the reviewer that the results from the primary and secondary GWASs could be communicated more clearly. We have rewritten the section on page 7 that now reads: "All three genome-wide significant loci demonstrated higher effect sizes in ADHD+DBDs compared to ADHDwoDBDs (Supplementary Table 1). In particular, the effect sizes for the loci located on chromosomes 7 and 11 (OR_{chr7}= ORs 1.199; OR_{chr11}=1.164) remained strong in the ADHD+DBDs vs. ADHDwoDBDs GWAS (OR_{chr7} 1.128; OR_{chr11}= 1.126) confirming a stronger effect of the risk allele for these two loci in

ADHD+DBDs compared to ADHDwoDBDs (Supplementary Table 1). The difference was most striking for rs7118422 on chromosome 11 (ADHD+DBDs: OR=1.164, P=8.97x10^{-9}), which showed no evidence for association with ADHDwoDBDs (OR=1.022, P=0.175) versus a suggestive evidence of association with ADHD+DBDs vs. ADHDwoDBDs (OR=1.126, P=7.07 x10^{-04}).”

This odd emphasis is repeated throughout the manuscript where the results of the case-only analyses are reported or discussed:

Page 8 paragraph 3

Page 9 Paragraph 2

Page 12 Paragraph 1

Page 13 bottom

<u>Page 8, paragraph 3:</u> When reporting results for single markers we can directly compare effect sizes in the primary GWAS with effect sizes in the two secondary GWASs. However, this comparison is not possible for the gene-set analyses (reported on page 8), since these analyses include a gene-set identified based on the results from the primary GWAS which is tested in the two secondary GWASs. It is therefore not possible to rewrite the section on page 8, in the same way as we did for the single marker analyses.

<u>Page 9, paragraph 2</u>: In order to estimate how much of the phenotypic variance that can be explained by common genetic variation we estimate the SNP heritability (h^2_{SNP}) for ADHD+DBDs and ADHDwoDBDs, and the estimates for these two phenotypes are compared statistically. Then we also estimate h^2_{SNP} for ADHD+DBDs within ADHD (variance explained by common variants among cases only). This analysis is only done with the purpose to clarify how much of the variance in ADHD+DBDs among ADHD cases can be explained by common variance, and we do not compare the latter h^2_{SNP} estimate to any of the other two h^2_{SNP} estimates. So, we do not think it is relevant to rewrite this section, but if we have misunderstood the point raised by the reviewer, we will of course be willing to change the text.

<u>Page 12 Paragraph 1:</u> We have re-written the section on page 12, according to the point raised by the reviewer: “This locus seems to be specifically associated with the aggressive and disruptive component of the ADHD+DBDs phenotype, since the effect disappeared in the ADHDwoDBDs GWAS. This was further supported in the GWAS comparing comorbid ADHD+DBD to ADHDwoDBDs where the locus remained strongly associated, however not genome-wide significant (Supplementary Table 1).”

<u>Page 13 bottom:</u> We have re-written the section on page 13, according to the point raised by the reviewer: “The top-associated genes (79 genes) seem to mainly reflect association with the aggressive and disruptive component of the ADHD+DBDs phenotype. The geneset was significantly associated with ADHDwoDBDs but even more strongly associated in the ADHD+DBDs vs. ADHDwoDBDs GWAS (where the effect of ADHD is corrected out). Likewise, the most strongly associated single markers (with P<1x10^{-4}) in the GWAS meta-analysis of ADHD+DBDs showed high consistency in the

direction of association in the GWAS of ADHDwoDBDs, but even higher consistency in the ADHD+DBDs vs. ADHDwoDBDs GWAS, reinforcing the notion that the associations mainly reflect the aggressive and disruptive component of the phenotype."

Secondly, it would greatly aid the reader if the authors used the term "ADHD-DBDs" rather than "ADHD without DBDs". Similarly, the case-only analyses could be described as "case-only analyses of DBDs" or "ADHD+/-DBDs analyses" rather than "ADHD+DBDs compared to ADHD without DBDs". These should be used consistently including replacing "ADHD alone" and "ADHDwoDBDs"; as seen on page 8 and supplementary table 6.

We agree with the reviewer that it would help the reader if we shorten the names used for the two secondary GWASs. We have changed "ADHD without DBDs GWAS" to "ADHDwoDBDs GWAS" throughout the manuscript.
The term "ADHD+DBDs vs. ADHD-only GWAS" has been changed to "ADHD+DBDs vs. ADHDwoDBDs GWAS" throughout the manuscript. The modified name for the case-only GWAS is still long, but we have chosen the current version as it precisely specifies which phenotypes are included and we have included the "vs." term, to remind the reader that the analysis only include cases.
In the current version we have continued to use the term ADHD+DBDs when referring to the primary GWAS meta-analysis. One could argue that it would streamline naming if we change this to ADHDwDBDs, however this could more easily be mistaken for ADHDwoDBDs. If the editor/reviewer prefer ADHDwDBDs over ADHD+DBDs we will of course be willing to change the term.

Thirdly, Page 15 replace "underlying biological cause" with "underlying genetic cause". This follows my comments on the previous draft and I had hoped that this had been corrected. Could the authors also agree replace "distinct" with "specific" on Page 16?

We have changed "underlying biological cause" to "underlying genetic cause" on page 15, and "distinct" has been changed to "specific" on page 16.

Additional comments:

i) In the abstract the authors state that" Genetic correlatins of ADHD+DBDs with aggressive (rg=0.81) and anti-social behaviors (rg=0.82)were high....." This is true but they not significantly higher when the two iPSYCH subsamples were compared (Page 10 paragraph 1, rg =0.85 & 0.92 in ADHD+DBDs versus rg = 0.74 & 0.56). So this statement may be misleading and should be rephrased.

In the abstract we inform about the genetic correlations of ADHD+DBDs with the aggression related traits, but we do not compare these estimates to the genetic correlations found for ADHDwoDBDs, or even mention the genetic correlations of ADHDwoDBDs with aggression. We think it is informative and important to state in the abstract that the genetic correlation of ADHD+DBDs with aggression related traits is high. In our opinion the sentence in the abstract states that as it is, without implying anything else. We would therefore prefer to keep the sentence as it is now.

ii) page 6

replace "Chines" with "Chinese"

replace "chromosome 11 locus to be a risk locus" with "chromosome 11 locus is a risk locus"

We thank the reviewer for the comments which have been corrected in the revised version of the manuscript.

iii) The gene set test is described as using 79 genes on page 8 and 80 genes on page 20. Please check. Could you also report the results for all the genes used in this gene-set test in Supplementary Table 3.

We thank the reviewer for noticing the error, and we are sorry for any confusion. There are 79 genes in the gene-set, and the error has been corrected on page 20.
We have inserted the gene-based association results for the 79 genes in Supplementary Table 3.

iv) On page 9, consider using "Three genes were predicted to be differentially expressed" instead of "Three genes were predicted differentially expressed". It would also be useful to specify the brain regions here, although they are reported in the Supplementary table.

We have corrected the text on page 9 according to the suggestion from the reviewer.

v) Page 9 SNP heritability. Can the authors explain why multiple prevalences were tested for the SNP heritability and where the different values came from.

Studies have reported various estimates of the prevalence of DBDs among individuals with ADHD, as written in the introduction: "Different comorbidity rates of ADHD with DBDs have been reported, some studies have found that approximately 30-40% of children with ADHD have comorbid DBDs (ADHD+DBDs)[9-11], while a study of 1.92 million individuals from Denmark found that 17% of those with ADHD were also diagnosed with DBDs[8]. "
If we assume a prevalence of ADHD in the population of ~5% the results from studies of comorbidity with DBDs would result in a prevalence in the range of 1-2% for ADHD+DBDs. In order to make it clear to the reader where the prevalences come from we have added the following sentence on page 9: "We estimated h^2_{SNP} using a prevalence of ADHD+DBDs in the population of 1-2%, based on studies reporting that around 20-40% of individuals with ADHD have comorbid DBDs [8-11]."

vi) Page 10 paragraph 1. It should be made clear that the ADHD without DBDs analysis reported was tested analysing only the iPSYCH cohort.

We thank the reviewer for noticing that the genetic correlation analyses including results from the ADHD+DBDs GWAS could be described better. We have added information on page 10, regarding this: "In contrast, ADHDwoDBDs (analysed solely in iPSYCH) was only significantly correlated with aggression in children (r_g=0.74, P=4.6x10^{-5})."
Additionally, on page 23 we have added the following to the method section: "We also estimated the genetic correlations of ADHDwoDBDs with the two aggression related phenotypes, using the results from the ADHD+DBDs GWAS which only included individuals from the iPSYCH cohort. "

vii) Please correctly reference the jackknife method used as Shao & Wu, 1989 as implemented in LDSC. https://jstor.org/stable/2241717

We thank the reviewer for pointing us to the correct reference which has been added in the revised version.

viii) Page 11. I can see why the authors have reported the age at first birth results as they do, but it is more correct to say that the association is a negative association with age at first birth and then, as they do in the discussion, suggest that this likely reflects ADHD+DBDs individuals as having children at a younger age. Otherwise the stated result looks at odds with the results in Suppl Table 7 and Suppl Figure 11.

We have re-written the sentence on page 11 according to the reviewer´s suggestion: "Finally, PRS for having children at an older age was negatively associated with ADHD+DBDs compared to ADHDwoDBDs (Z=-4.40, P=8.4x10^{-6}, OR=0.9)."

ix) Page 13 replace "calcium signaling to be" with "calcium signaling is"

We have corrected the sentence according to the reviewer´s suggestion.

x) Page 13 replace "Six genes were exome wide" with "In gene-based analyses, six genes were"

We have corrected the sentence according to the reviewer´s suggestion.

xi) Page 15 replace "to play" with "play" and replace "of getting a" with "of a"

We have corrected the text on page 15 according to the reviewer´s suggestion.

xii) Page 15 Replace "We identified individuals with ADHD+DBDs to have increased" with We have shown that individuals with ADHD+DBDs have an increased"

We have corrected the sentence on page 15 according to the reviewer´s suggestion.

xiii) Consider rephrasing:

Methods pages

"Register register"

"aggregated by PGC" perhaps "provided by the PGC"

"preferred kept" with "preferentially retained"

"GWAS GWAS"

"acrosse"
Figure 2

"Freqeuncy"

We thank the reviewer for noticing the typos in the method section and Figure 2 legend, which have been corrected in the revised version of the manuscript.

Reviewer #3 (Remarks to the Author):

The authors have assiduously addressed my comments. For example, they conducted new analyses using iPSYCH to test for SNP heritability differences between ADHD+DBD vs ADHD-DBD, which they now show are significantly different.

One remaining concern is relevant to the authors' main argument that A+D has a more severe genetic risk load than A-D and suggest that this might in part be due to A+D links with aggressive behaviour. However, both A+D and A-D are genetically correlated with aggressive behaviour (.82 vs .74, not significantly different).

For this reason, I asked to see the genetic correlation between A+D and A-D. The authors report that the genetic correlation is 0.99. The authors say this was expected because all cases are diagnosed with ADHD. But isn't the point of the paper that ADHD with DBD differs genetically from ADHD without DBD? If the genetic correlation is 0.99, doesn't this mean that there is no difference genetically?

We have misunderstood the intention with the reviewers comment to the previous version of the manuscript. We assume that what the reviewer really would like to know is an estimation of the genetic correlation between ADHDwoDBDs and DBDs. Since we do not have DBDs without ADHD we cannot test this directly. However, we have addressed this in another way, by estimating the genetic correlation between ADHDwoDBDs and the genetic component that is specific to DBDs identified in the ADHD+DBDs vs. ADHDwoDBDs GWAS. In this analysis r_g=0.28 (SE=0.18; P=0.10), indicating that there is only a moderate overlap between ADHDwoDBDs and DBDs when adjusting for the effect of ADHD.

We have added this to the manuscript on page 10: "Additionally, we estimated the genetic correlation of ADHDwoDBDs wih the genetic component that is specific to DBDs. The latter identified in the ADHD+DBDs vs. ADHDwoDBDs GWAS, which is adjusted for the effect of ADHD. The genetic correlation was only moderate and not significantly different from zero r_g=0.29 (SE=0.18, P=0.10)."
We have added the following to the methods section on page 23: "The genetic correlation between ADHDwoDBDs and the genetic risk component specific to DBDs when correcting for the effect of ADHD, was estimated using LD score regression and the summary statistics from the ADHDwoDBDs GWAS and the ADHD+DBDs vs. ADHDwoDBDs GWAS."

<u>Other corrections not requested by the reviewers:</u>

We have corrected the term "polygenic risk score (PRS)" to "polygenic score (PGS)" throughout the manuscript. As it makes better sense to refer to a polygenic score for phenotypes such as educational attainment.

Reviewer #1 (Remarks to the Author):

The authors have responded appropriately to all my comments and corrected those that required correction. I look forward to seeing this paper published.

[Editor's note: we asked reviewer 1 to comment on reviewer 3's review. Reviewer 1's response is below]

1) Standard errors and p-value are not given for the stated rg of 0.99 which would allow a better assessment of the significance of the correlation.
2) There appears to be an under appreciation that the estimated correlation using LD score regression, as the authors did, can give point estimates for rg of greater than 1. So the value they quote is not bounded by 1. This can be caused by genotype-environment correlation. See https://www.nature.com/articles/ng.3406 Supplementary Table 4.
3) The rg should be interpreted in conjunction with the SNP h2, as low SNP h2 makes the results more difficult to interpret i.e. if only a small fraction of the h2 is captured by the GWAS summary statistics i.e. common SNPs, the meaning of the overlap of this small fraction with another trait becomes difficult to interpret. Although it is reasonable to assume that the correlation between two traits is similar for common and rare variants, this may not be the case.

I think that the authors need to provide the se and p-value on their genetic correlations and SNP h2 estimates and include them in the manuscript. This will allow the reader to assess the data in full. I don't agree with reviewer 3 that this correlation means that there is no genetic difference between A+D v A-D. Although the authors own statement that the correlation between A+D and A-D is driven by ADHD is unhelpful and not proven.

Finally the authors could add greater confidence by repeating the bivariate heritability calculation in GCTA using the individual datapoints rather than the summary data.

Reviewer #3 (Remarks to the Author):

As I said in my first review: 'Although I have raised several issues, I think this is an interesting paper, even though I am not convinced by their central point that ADHD plus Disruptive Behavior Disorder (A+D) is etiologically distinct from ADHD without Disruptive Behavior (A-D).'

The genetic correlation of 0.28 between A+D and D does not seem germane, especially given the circuitous way in which summary statistics for D were obtained.

If the point of the paper is that A+D is etiologically distinct from A-D, It seems very problematic that the genetic correlation between them is 0.99. It seems a weak argument to say that the D component of A+D 'is overridden by the high load of ADHD associated variants … in both groups'. A+D is what it is and A-D is what it is, and a genetic correlation of 0.99 indicates that they are not different genetically.

I am also not persuaded by the argument that two traits can correlate differently with polygenic scores even though their genetic correlation is 1.0 – unless the genetic correlation is poorly estimated or the different correlations with polygenic scores are sampling errors.

Surely the authors would not have expected that the genetic correlation between A+D and A-D would be near unity given their central hypothesis that A+D and A-D are genetically distinct.

REVIEWER COMMENTS

Reviewer #1 (Remarks to the Author):

The authors have responded appropriately to all my comments and corrected those that required correction. I look forward to seeing this paper published.

We thank the reviewer for valuable input and the concluding positive remark.

[Editor's note: we asked reviewer 1 to comment on reviewer 3's review. Reviewer 1's response is below]

1) Standard errors and p-value are not given for the stated rg of 0.99 which would allow a better assessment of the significance of the correlation.

In the previous version of the manuscript we had actually removed the rg estimate between ADHD+DBDs and ADHDwoDBDs, but based on the comment from reviewer 3, we have now re-inserted this estimate and removed the rg estimate of ADHD+DBDs with "ADHDwoDBDs vs ADHD+DBDs".
As requested by reviewer 1, we have included the standard error in the text on page 13: "*Finally, we estimated the genetic correlation of ADHD+DBDs vs ADHDwoDBDs (r_g=0.99, SE=0.07, P=2.64x10^{-45}).*"

2) There appears to be an under appreciation that the estimated correlation using LD score regression, as the authors did, can give point estimates for rg of greater than 1. So the value they quote is not bounded by 1. This can be caused by genotype-environment correlation. See
https://www.nature.com/articles/ng.3406 Supplementary Table 4.

We agree with the reviewer. To complement the LD score regression analysis, we have added GCTA estimate of the genetic correlation of ADHD+DBDs with ADHDwoDBDs, corroborating the LD score results (see point below).

3) The rg should be interpreted in conjunction with the SNP h2, as low SNP h2 makes the results more difficult to interpret i.e. if only a small fraction of the h2 is captured by the GWAS summary statistics i.e. common SNPs, the meaning of the overlap of this small fraction with another trait becomes difficult to interpret. Although it is reasonable to assume that the correlation between two traits is similar for common and rare variants, this may not be the case.

We agree with the reviewer that the SNP heritability should be considered when interpreting results from genetic correlation analyses. Accordingly, we have added the following to the discussion, page 18: "*It should also be noted that the SNP heritabilities of childhood aggression[33] and antisocial behavior[34] are at the low end (0.05 and 0.06, respectively), and probably downwards biased by heterogeneity in the cohorts analyzed[33]. The studies do therefore not capture the full impact of common variants in aggressive behavior, and the observed high genetic correlation of the two phenotypes with ADHD+DBDs involves variants that only explain a small proportion of variance in aggression.*"

I think that the authors need to provide the se and p-value on their genetic correlations and

SNP h2 estimates and include them in the manuscript. This will allow the reader to assess the data in full. I don't agree with reviewer 3 that this correlation means that there is no genetic difference between A+D v A-D. Although the authors own statement that the correlation between A+D and A-D is driven by ADHD is unhelpful and not proven.

Thanks for noticing this. **We have added standard errors in relation to the SNP-heritability estimates where they were missing (on page 17 in the discussion).**
Genetic correlation values and P-values are reported on page 13, where we have added the standard errors as requested.

We note that reviewer 1 "don't agree with reviewer 3 that this correlation (i.e. *ADHD+DBDs vs ADHDwoDBDs)* means that there is no genetic difference between A+D v A-D".
We clearly agree with reviewer 1 and have expanded our argumentation, including testing genetic correlations in a similar way (comorbid vs not-comorbid cases) in two disorders with known genetic differences (ADHD and autism spectrum disorder, please see response to reviewer 3, item 3)).

Finally the authors could add greater confidence by repeating the bivariate heritability calculation in GCTA using the individual datapoints rather than the summary data.

We do not have access to individual genotypes for the aggression-related phenotypes, so we cannot use GCTA to estimate genetic correlations with these phenotypes.

We have included the GCTA estimate of the genetic correlation of ADHD+DBDs vs ADHDwoDBDs and added the following to the methods section, page 26: "*The genetic correlation between ADHD+DBDs and ADHDwoDBDs was calculated using LD score regression and summary statistics from the GWAS meta-analysis of ADHD+DBDs and the GWAS of ADHDwoDBDs, the latter based on iPSYCH data only. To supplement the LD score regression analysis we used used iPSYCH genotypes and GCTA to estimate the genetic correlation between ADHD+DBDs (2,155 cases and 11,659 controls) and ADHDwoDBDs (13,583 cases and 11,250 controls), showing results consistent with LD score regression (r_g=0.97; SE=0.06), and not statistically different from one*."

Reviewer #3 (Remarks to the Author):

As I said in my first review: 'Although I have raised several issues, I think this is an interesting paper, even though I am not convinced by their central point that ADHD plus Disruptive Behavior Disorder (A+D) is etiologically distinct from ADHD without Disruptive Behavior (A-D).'

The genetic correlation of 0.28 between A+D and D does not seem germane, especially given the circuitous way in which summary statistics for D were obtained.

Based on the reviewers comment we have now excluded the rg estimate of 0.28. We replaced it with the previously included rg estimate of ADHD+DBDs vs ADHDwoDBDs (r_g=0.99, SE=0.07, P=2.64x10^{-45}), calculated using LD score regression and summary statistics from the GWAS meta-analysis of ADHD+DBDs and GWAS of ADHDwoDBDs. To supplement the LD score analysis, we also included a GCTA analysis showing similar results (r_g=0.97; SE=0.06), as written on page 26.

If the point of the paper is that A+D is etiologically distinct from A-D, It seems very problematic that the genetic correlation between them is 0.99. It seems a weak argument to say that the D component of A+D 'is overridden by the high load of ADHD associated variants … in both groups'. A+D is what it is and A-D is what it is, and a genetic correlation of 0.99 indicates that they are not different genetically.

I am also not persuaded by the argument that two traits can correlate differently with polygenic scores even though their genetic correlation is 1.0 – unless the genetic correlation is poorly estimated or the different correlations with polygenic scores are sampling errors.

Surely the authors would not have expected that the genetic correlation between A+D and A-D would be near unity given their central hypothesis that A+D and A-D are genetically distinct.

Epidemiological studies have consistently demonstrated that ADHD+DBDs is a more severe phenotype than ADHD, and twin studies have suggested that ADHD+DBDs might differ genetically from individuals only having ADHD (see also the Introduction section). Since very little is known about the genetic architecture of the comorbid phenotype, we wanted to investigate the common variant architecture of this phenotype further.
We provide strong evidence for a novel (the first-ever) locus specific to ADHD+DBDs (see 1 below) and demonstrate significant increased polygenic loads in ADHD+DBDs compared to ADHDwoDBDs (see 2 below); results which we believe are important to the field. It is not within the scope of this study to prove that ADHD+DBDs and ADHDwoDBDs are distinct at the polygenic level, and we actually do not think we have the data to do that (see 3 below).

1) **We identified three genome-wide significant loci and dissected the signals in order to evaluate which phenotype primarily drives the signals. We found strong evidence that the chromosome 11 locus is specific to ADHD+DBDs. The index variant rs7118422 was genome-wide significantly associated with ADHD+DBDs (OR=1.164, P=8.97x10^{-9}), but showed no evidence of association with ADHDwoDBDs (OR=1.022, P=0.175). In the two sensitivity analyses where we corrected out the effect of ADHD, the variant still demonstrated strong nominal association: I) in the case-only GWAS (ADHD+DBDs vs. ADHDwoDBDs, OR=1.126, P=7.07 x10^{-04} and II) in the mtCOJO ADHD+DBDs GWAS corrected for ADHDwoDBDs (OR$_{adjusted}$=1.14; P$_{adjusted}$=1.43x10^{-06}). Additionally, the locus demonstrated consistent association across the included cohorts and replicated in the Chinese cohort. Based on these results we believe we have a strong case for the chromosome 11 locus being specific to the comorbid phenotype.**

2) **Regarding the polygenic architecture; we show that individuals with ADHD+DBDs have a *significantly different* polygenic architecture compared to ADHDwoDBDs, when it comes to the polygenic load for seven phenotypes (e.g. aggression, ADHD and educational attainment). However, we agree with the reviewer that we do not know if the increased polygenic load is *specific* to DBDs (in the context of ADHD). Even though it would be very interesting to clarify whether the observed difference is caused by a distinct/specific genetic DBDs component or by a stronger polygenic load of variants that are shared with ADHDwoDBDs, the present study is the first to demonstrate that comorbid individuals differ significantly from ADHDwoDBDs at the common variant**

polygenic level for additional six phenotypes (it has previously been found that PGS-ADHD is increased in ADHD+DBDs compared to ADHDwoDBDs).

We would like to stress that in the current version of the manuscript, we do not state that the difference is caused by a distinct DBDs polygenic component. In order to stress this further we have included the following sentence in the discussion page 18: "*However, our results do not reveal whether the increased polygenic load of variants associated with aggression observed in ADHD+DBDs is caused by variants specific to DBDs or due to a general increased load of variants that are also shared with ADHD.*"

3) **Regarding the observed high genetic correlation of ADHD+DBDs vs ADHDwoDBDs; we do not think that we can exclude that ADHD+DBDs cases have a specific genetic risk component beyond what is shared with ADHD even though we observe a high genetic correlation (rg=0.99). When we estimate rg between ADHD+DBDs and ADHDwoDBDs all individuals are diagnosed with ADHD in the two case groups. Rg is the slope of Z_1xZ_2, where Z1 and Z2 are the z-scores for each variant in the two GWASs. This means that variants associated with ADHD-only and ADHD+DBDs are expected to have similar z-scores in both GWASs driving the rg estimate towards one. If only a small part of the variants are specifically associated with DBDs (and not strongly due to the relatively small number of comorbid cases), this could have such a small impact that it will not be detected by the genetic correlation analysis.**

In order to explore this further we performed a number of genetic correlation analyses mirroring the analyses of the current study but assessing different disorders, namely ADHD and autism spectrum disorder (ASD). ADHD and ASD have both shared and distinct common genetic risk variants (Demontis et al. Nature Genetics 51, 63-75, 2019; Grove et al. Nature Genetics 51, 431-444, 2019). We estimated three genetic correlations using GCTA and genotypes from individuals diagnosed with ADHD and ASD in the iPSYCH cohort. The analysis was performed on a genetic homogenous sample and we corrected the analyses using relevant covariates:

I) ADHD without ASD (ADHDwoASD) (10,233 cases, 8858 controls) vs ASD without ADHD (ASDwoADHD) (12,367 cases, 11161 controls)

II) comorbid ADHD+ASD (2,674 cases, 11161 controls) vs ADHDwoASD (10,233 cases, 8858 controls)

III) comorbid ADHD+ASD (2164 cases, 8858 controls) vs ASDwoADHD (12,367 cases, 11161 controls)

Consistent with the literature (Grove et al. Nature Genetics 51, 431-444, 2019; Cross-Disorder Group of the Psychiatric Genomics Consortium, Cell 179(7):1469-1482, 2019), the genetic correlation between ADHDwoASD and ASDwoADHD was moderate (rg=0.397, SE=0.06) (also illustrated in the figure below), but when estimating the genetic correlation of the comorbid group with each of the single disorders the genetic correlations increased remarkably (ADHD+ASD vs ADHDwoASD rg=0.767, SE=0.11; ADHD+ASD vs ASDwoADHD rg=0.865, SE=0.14) and the standard error for ADHD+ASD vs ASDwoADHD even overlapped 1.

We think this demonstrates that genetic correlations for phenotypes with distinct genetic components can approach 1 when the genetic correlation estimates are between comorbid individuals and one of the single disorders.

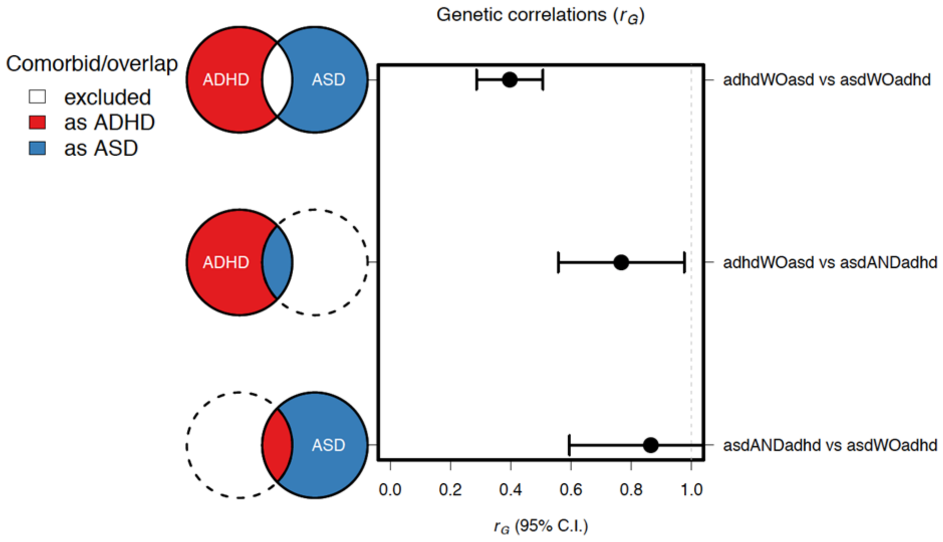

Reviewer #1 (Remarks to the Author):

The authors have gone to great lengths to satisfy the reviewers and I think that the latest additions add significantly to the clarity of their arguments.

REVIEWERS' COMMENTS

Reviewer #1 (Remarks to the Author):

The authors have gone to great lengths to satisfy the reviewers and I think that the latest additions add significantly to the clarity of their arguments.

We thank the reviewer for the positive feedback and we are happy that any doubts have been clarified.
